# Function and Evolution of DNA Methylation in *Nasonia vitripennis*

**DOI:** 10.1371/journal.pgen.1003872

**Published:** 2013-10-10

**Authors:** Xu Wang, David Wheeler, Amanda Avery, Alfredo Rago, Jeong-Hyeon Choi, John K. Colbourne, Andrew G. Clark, John H. Werren

**Affiliations:** 1 Department of Molecular Biology and Genetics, Cornell University, Ithaca, New York, United States of America; 2 Cornell Center for Comparative and Population Genomics, Cornell University, Ithaca, New York, United States of America; 3 Department of Biology, University of Rochester, Rochester, New York, United States of America; 4 School of Biosciences, The University of Birmingham, Birmingham, United Kingdom; 5 Cancer Center, Department of Biostatistics and Epidemiology, Georgia Regents University, Augusta, Georgia, United States of America; New York University, United States of America

## Abstract

The parasitoid wasp *Nasonia vitripennis* is an emerging genetic model for functional analysis of DNA methylation. Here, we characterize genome-wide methylation at a base-pair resolution, and compare these results to gene expression across five developmental stages and to methylation patterns reported in other insects. An accurate assessment of DNA methylation across the genome is accomplished using bisulfite sequencing of adult females from a highly inbred line. One-third of genes show extensive methylation over the gene body, yet methylated DNA is not found in non-coding regions and rarely in transposons. Methylated genes occur in small clusters across the genome. Methylation demarcates exon-intron boundaries, with elevated levels over exons, primarily in the 5′ regions of genes. It is also elevated near the sites of translational initiation and termination, with reduced levels in 5′ and 3′ UTRs. Methylated genes have higher median expression levels and lower expression variation across development stages than non-methylated genes. There is no difference in frequency of differential splicing between methylated and non-methylated genes, and as yet no established role for methylation in regulating alternative splicing in *Nasonia*. Phylogenetic comparisons indicate that many genes maintain methylation status across long evolutionary time scales. *Nasonia* methylated genes are more likely to be conserved in insects, but even those that are not conserved show broader expression across development than comparable non-methylated genes. Finally, examination of duplicated genes shows that those paralogs that have lost methylation in the *Nasonia* lineage following gene duplication evolve more rapidly, show decreased median expression levels, and increased specialization in expression across development. Methylation of *Nasonia* genes signals constitutive transcription across developmental stages, whereas non-methylated genes show more dynamic developmental expression patterns. We speculate that loss of methylation may result in increased developmental specialization in evolution and acquisition of methylation may lead to broader constitutive expression.

## Author Summary

Insects use methylation to modulate genome function in a different manner from vertebrates. Here, we quantified the global methylation profile in a parasitic wasp species, *Nasonia vitripennis*, a model with some advantages over ant and honeybee for functional and genetic analyses of methylation, such as short generation time, inbred lines, and inter-fertile species. Using a highly inbred line permitted us to precisely characterize DNA methylation, which is compared to gene expression variation across developmental stages, and contrasted to other insect species. DNA methylation is almost exclusively on the 5′-most 1 kbp coding exons, and ∼1/3 of protein coding genes are methylated. Methylated genes tend to occur in small clusters in the genome. Unlike many organisms, *Nasonia* leaves nearly all transposable element genes non-methylated. Methylated genes exhibit more uniform expression across developmental stages for both moderately and highly expressed genes, suggesting that DNA methylation is marking the genes for constitutive expression. Among pairs of differentially methylated duplicated genes, the paralogs that lose DNA methylation after duplication in the *Nasonia* lineage show lower expression and greater specialization of expression. Finally, by comparative analysis, we show that methylated genes are more conserved at three different time scales during evolution.

## Introduction

DNA methylation is an important epigenetic modification found in many plants and animals [1]–[5]. In mammals, DNA methylation is associated with important epigenetic processes such as genomic imprinting [6], histone modifications and X chromosome inactivation [7], [8], and plays an important role in brain development [9]. Clusters of CpG sites (CpG islands or CGIs) are often found in the 5′ regulatory regions including the promoter regions in mammals [10], [11]. Methylation at the promoter will typically result in silencing of the gene [12]. The promoters of transposable elements (TEs) are also often repressed by DNA methylation [13]. Non-CpG methylation has been observed in mammals, with high percentages in embryonic stem cells [14].

DNA methylation is also widespread in invertebrates [4], [15]–[26]. In contrast with mammals, methylation typically occurs over gene bodies, and is correlated with elevated gene expression [4], [15], [16], [18], [19], [22], [27], rather than gene inactivation. Consistent with gene activation, several studies of invertebrate methylation have reported that methylated genes tend to have “house-keeping functions”, whereas non-methylated genes are more tissue-specific [18], [28], [29].

DNA methylation is not universal among invertebrates [30], [31]. For example, the fruit fly *Drosophila melanogaster* lacks DNA methylation in adults due to the loss of two of three DNA methyltranferases (*Dnmt1 and Dnmt3*), and the reported DNA methylation found in early embryonic stages [32], [33] may be due to bisulfite conversion artifacts [31]. Nevertheless, in insects a combination of insect genome sequencing, identification of a full complement of DNMTs, and indirect or direct quantification of methylation, has uncovered genome-wide methylation in many species. A common indirect computational method for identifying genome-wide methylation is gene specific depletion of expected frequencies of CpG relative to observed (CpG O/E), which occurs in methylated genes due to mutational biases of methylated C to T [16]. This approach yielded evidence of genome-wide methylation in a number of insects, including the honeybee *Apis mellifera*, parasitoid wasp *Nasonia vitripennis*, pea aphid *Acrythiosiphon pisum*, and others [16], [26], [34]–[36]. Direct methods that have been used to quantify genome-wide methylation in insects include methylation sensitive restriction enzymes [37] and methylated DNA immunoprecipitation (MeDIP) [21]. However, to achieve single-base resolution of methylation in the genome requires bisulfite conversion coupled with high throughput sequencing, which has so far only been reported for honeybee (*Apis mellifera*) [15], [19], silkworm (*Bombyx mori*) [15], [22] and ants *Camponotus floridanus*, *Harpegnathos saltator* [18] and *Solenopsis invicta* [38].

Most of the work on arthropod DNA methylation has focused on the social insects (honeybees and ants) where alternative castes drive an interest in developmental processes that modulate caste determination [18], [28], [29]. Investigations in honeybee and ants have suggested an association between alternate splicing and methylation [18], [39]. *N. vitripennis* is a non-social haplodiploid parasitoid wasp with a well annotated reference genome [34], [40], [41]. Prior studies have revealed DNA methylation in *Nasonia* [20] and the presence of requisite DNA methyltransferases, including three members of *Dnmt1* [34]. Here, we report findings of a whole-genome bisulfite sequencing (WGBS-seq) study that provides base-pair resolution of the genome of *Nasonia vitripennis*, a non-social Hymenopteran species [34], [40], [41]. The highly inbred strain of *Nasonia* used here allows for precisely mapping of WGBS-seq reads and CpG methylation calls to the genome without the complications caused by SNP variation found within heterologous DNA samples from variable strains or populations. We analyze whole genome patterns of DNA methylation in *N. vitripennis*, including the relationship between methylation, gene expression, expression breadth, and gene length, clustering of methylated CpG sites and methylated genes in the genome, patterns of methylation among transposons, non-CpG methylation, methylome comparisons with *Apis*, and changes in gene expression correlated with changes in methylation among paralogs in the *Nasonia* lineage. The *Nasonia* methylome helps to shed light on the function(s) and evolution of DNA methylation in insects.

## Results

### A. Base-pair resolution profile of CpG DNA methylation in *Nasonia vitripennis*

To profile the *Nasonia* methylome, we performed Illumina whole-genome bisulfite sequencing (WGBS-seq) in adult female samples with 25× haploid genome coverage (Figure S1) and 16.2× average CpG coverage (Figure S2). From the control lambda DNA alignments, the bisulfite conversion efficiency was 99.7% (Table S1), indicating highly efficient conversion. Additional quality control metrics and procedures to assure the high quality of this methylome are described in Materials and Methods.

Across the 8 million CpG sites in the *Nasonia* genome covered by our data, the average percentage methylation is 1.45%, and 1.6% of sites are defined as methylated CpG sites (mCpG) based on our criteria of the site having at least 10× coverage and >10% methylation (see Materials and Methods, Table 1 and Table S2). The percentage of methylation is not uniform across mCpG sites – those with 100% methylated sites are highly enriched, and the distribution is biased toward highly methylated sites with >75% methylation (Figure S3). In other words, CpG sites tend to either be largely non-methylated or highly methylated. We established that genome-wide bisulfite sequencing correctly identifies methylated and non-methylated CpGs by sequencing multiple clones from bisulfite converted DNA from three randomly chosen methylated genes and three non-methylated genes (Figures S4 S5, S6, S7, S8, S9 and Text S1).

**Table 1:** Summary of DNA methylation status for CpG islands and methylated CpG clusters.

	CpG islands	methylated CpG clusters	Genome
Criteria	200 bp–10 kbp, GC% >50%, CpG O/E >0.6	mCpG/covered CpG >80%, average methylation% >40%	-
Counts/Average length	9265/723 bp	5440/1.2 kbp	-
Total length (% of genome)	6,701,356 (2.3%)	6,596,158 (2.2%)	295.1 Mbp
Total number of CpGs (% of genome)	609,994 (4.35%)	109,676 (0.78%)	14,024,488
CpG density (fold of genome average)	9.1% (1.90)	1.7% (0.35)	4.8%
Number of covered CpGs (% of genome)	139,484 (1.8%)	97,310 (1.2%)	7,818,889
Number of mCpGs (% of genome)	177 (0.15%)	91,803 (78.5%)	116,929
Methylation percentage (mCs/all CpG reads)	0.16% (4,405/2,814,740)	64.91% (2,205,276/3,397,307)	1.45%
In intergenic regions	3,412 (36.8%)	65 (1.20%)	-
			

mCpG: methylated CpG sites; mC: methylated cytosines.

Below we describe some of the striking patterns observed in the methylome of *Nasonia*.

#### A.1. CpG methylation occurs on gene bodies and is enriched in the 5′ coding region

**Figure 1:**
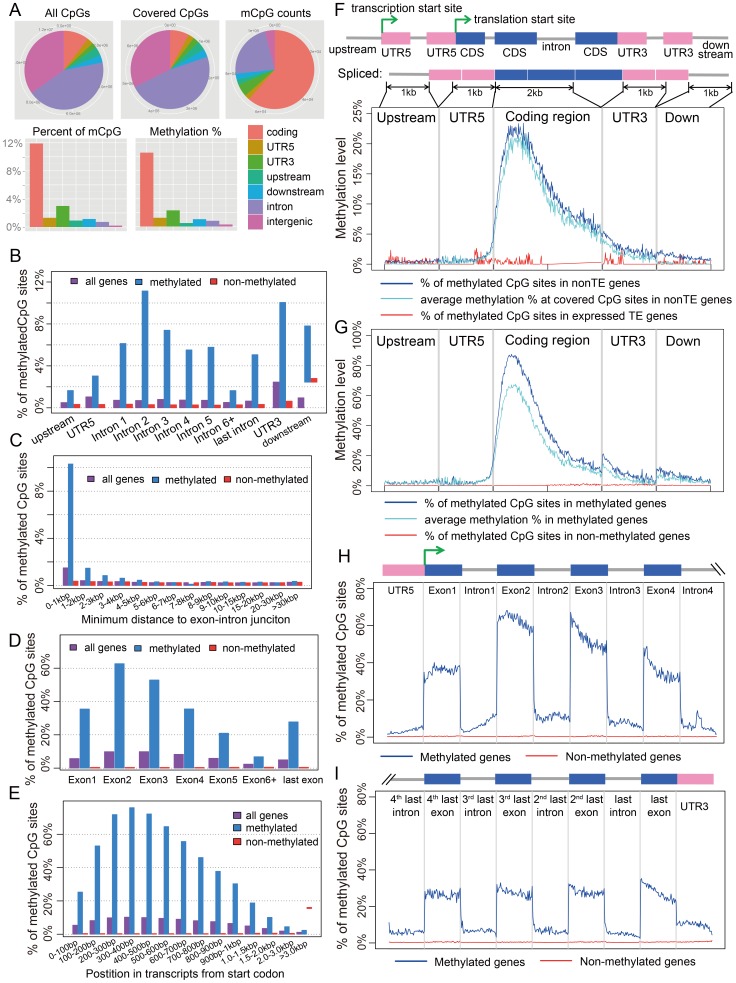
Distribution of CpG DNA methylation in the *Nasonia* genome across protein-coding genes. (A) Distributions across genomic features for all 14 million CpG sites (Top left), 8 million covered CpG sites (Top middle) and methylated CpG sites (mCpGs, Top right). Plotted in the bottom panel are the distributions for percentage of mCpGs and methylation percentage at covered CpG sites. (B) Percentage of mCpGs in the 1 kbp upstream, 1 kbp downstream, UTR and intronic regions for methylated (blue), non-methylated (red) and all genes (purple). (C) Percentage of mCpGs in introns for methylated (blue), non-methylated (red) and all genes (purple), binned by the nearest distance to the exon-intron junctions. (D) Percentage of mCpGs across exons for methylated (blue), non-methylated (red) and all genes (purple). (E) Percentage of mCpGs in the coding region starting from first codon for methylated (blue), non-methylated (red) and all genes (purple). (F) Methylation level in 1 kbp upstream, 1 kbp 5′-UTR, first 2 kbp coding, 1 kbp 3′-UTR and 1 kbp downstream regions for 1,540 expressed transposable element genes (TE genes) and 16,186 non-TE genes. Dark blue line: percentage of mCpGs for non-TE genes; light blue line: average methylation percentage across covered CpGs for non-TE genes; red line: percentage of mCpGs for TE genes. (G) Methylation level in 1 kbp upstream, 1 kbp 5′-UTR, first 2 kbp coding, 1 kbp 3′-UTR and 1 kbp downstream regions for 4,751 methylated non-TE genes and 12,975 non-methylated non-TE genes. Dark blue line: percentage of mCpGs for methylated genes; light blue line: average methylation percentage across covered CpGs for methylated genes; red line: percentage of mCpGs for non-methylated genes. (H–I) Plot of Percentage of methylated CpG sites in the 5′UTR, the first four exons and introns (H) and 3′UTR, the last four exons and introns (I) for methylated (blue) and non-methylated genes (red). All exons, introns and UTRs were rescaled to the same length.

DNA methylation in *Nasonia* predominantly occurs over gene bodies, and in particular over exons (Figure 1). While only containing 10% of 14 million CpGs, the annotated coding regions in *Nasonia* OGS2 (Official Gene Set v2; see Evidential Genes for *Nasonia vitripennis* at http://arthropods.eugenes.org/genes2/nasonia/) [42] are significantly enriched for mCpGs (61.4%, *P*-value<2.2×10^−16^, Chi-squared test; Figure 1A). Overall, 11.9% of CpGs located in exons are methylated. By contrast, the intergenic (0.2%), intronic (0.7%) and 1 kbp flanking regions of genes (1%) are depleted of methylated CpGs (Figure 1A). mCpGs are also clustered in the *Nasonia* genome, 78.5% of which are found in 5,440 clusters (Table 1 and Text S2). 98.8% of mCpG clusters are in gene regions (Table 1), which is consistent with gene body methylation. Furthermore, among the 65 mCpG clusters in “intergenic” regions, we found detectable expression in adult female RNA-seq data for 42 (Table S3). We therefore conclude that methylated CpG islands in *Nasonia* occur almost exclusively within transcribed genes.

To compare *Nasonia* mCpG clusters to mammalian-type CpG islands, we ran predictions of CpG islands in the *Nasonia* genome using the same criteria as in mammals [20] (see Materials and Methods). Of 9,265 CpG islands, 36.8% occurred outside of gene bodies and these were nearly universally not methylated (0.15% mCpGs). Methylation also shows a clear pattern of being enriched at the beginning of genes (Figure 1C,F,G). Based on this pattern, we define genes with >10% methylated CpGs in the first 1 kbp coding region as methylated genes, and genes with ≤10% methylated CpGs in the first 1 kpb as non-methylated genes (see Materials and Methods). Methylation is largely absent in genes defined as non-methylated (0.31% mCpGs) (Figure 1B–I). In methylated genes, the highest levels of methylation occur in the 5′ exons of genes classified as methylated, and decline toward the 3′ region of the gene (Figure 1G–I). Exon methylation in methylated genes peaks at exon 2 or 400–500 bp into the coding region (Figure 1D–H); intron methylation was observed around the exon-intron junctions and also peaks at intron 2 (Figure 1B,C,H). In *N. vitripennis*, 26.7% of protein-coding genes are methylated among the 17,726 genes for which we have sufficient coverage to score methylation status. Excluding 1,540 expressed transposon genes, 4,739 (29.3%) of protein-coding gene are methylated. Our genome-wide investigation also confirms an association between the ratio of observed to expected CpG (CpG O/E) and DNA methylation status, a pattern that was predicted earlier based on bisulfite sequencing of 18 individual genes [20] (Figure S10 and Text S3).

#### A.2. Transposons are rarely methylated

Among 17,726 annotated genes in OGS2 with adequate uniquely mapped read coverage, 1,540 are expressed transposable element genes (expressed TE genes). The TE genes were characterized in OGS2 with detectable expression level in at least one developmental stage [42]. In adult females, 99.8% of these TE genes are non-methylated (Figure 1F). However, because many TEs occur in multiple copies in the genome with insufficient divergence to be uniquely mapped, we also quantified the DNA methylation percentages in 839 repetitive TEs annotated in the *Nasonia* genome paper [34] that were not covered by uniquely mapped reads (see Materials and Methods). Among the 803 elements with adequate WGBS-seq coverage, only five (GYPSY, SPRINGER, SNAKEHEAD, IFAC and BLASTOPIA) have >5% methylation averaging across CpG positions, and the top three are highly expressed in adult female RNA-seq data (Table S4). Therefore, we can conclude that TEs are rarely methylated and when they are, it can be associated with activation rather than inactivation. This finding contrasts sharply with methylation patterns in plants and mammals, in which methylation of TEs is involved in transcription suppression [13], [43].

#### A.3. CpG methylation shows a strong exon/intron pattern, and “marks” the beginning and end of protein-coding regions

There is a strong exon/intron patterning to methylation, with significantly heavier methylation levels occurring over exons, and declining in adjacent introns (Figure 1H, I). For example, there is significantly higher methylation in both the leading (*P*-value<2.2×10^−16^, Wilcoxon Matched-Pairs Signed Ranks Test - WMSRT) and trailing coding exons (*P*-value<2.2×10^−16^, WMSRT) relative to the intervening intron between the first two 5′ coding exons. The pattern persists even as overall methylation level decreases toward the 3′ regions of genes (Figure 1H, I).

In addition, the protein-coding regions of methylated genes are enriched for methylation relative to flanking untranslated regions (UTRs) of the same genes. For methylated genes, only 3.0% of the covered CpGs are methylated in the 5′ UTRs (Figure 1B) compared to 35.5% in the first coding exons (Figure 1D). Levels of methylation increase following the start codon for protein-coding genes, with significantly lower levels of mCpGs within 500 bp 5′ of the start codon (1336/26350 or 5.1% mCpGs) relative to 500 bp 3′ of the start codon (30544/46513 or 65.7% mCpGs; (Figure 1G; *P*-value<2.2×10^−16^, Chi-squared test). We are confident in the UTR identifications for *Nasonia* OGS2 because they are based on extensive RNA sequencing and tiling array data (see http://arthropods.eugenes.org/genes2/nasonia/), and consistent with our own adult RNA-seq data.

For smaller genes (*e.g.* with coding region <1 kbp), the end of the protein-coding region after the stop codon is also “marked” by reduced methylation level (Figure 1H). Comparison of methylation levels 100 bp before and 100 bp after the stop codon (with ≥4 covered CpGs) shows a significant decline in methylation level of the 3′UTR in genes with protein coding regions <1 kbp (11.4% mean before, 5.7% mean after, *P*-value = 0.003, WMSRT). The same does not hold, however, for genes of greater length (1.6% mean before, 1.6% mean after, *P*-value = 0.97, WMSRT). For genes shorter than 1 kbp, the relative number of genes with higher mCpG percentage before the stop codon is also significantly greater than those with lower mCpG percentage (*P*-value = 4.2×10^−7^, Chi-squared test), but this is not the case for larger genes (*P*-value = 0.21, Chi-squared test). Implications of the apparent tagging of the protein-coding exons and start codon from methylated genes are explored in the Discussion.

#### A.4. Correlation of transcript length and methylation is driven by 5′ bias in methylation

**Figure 2:**
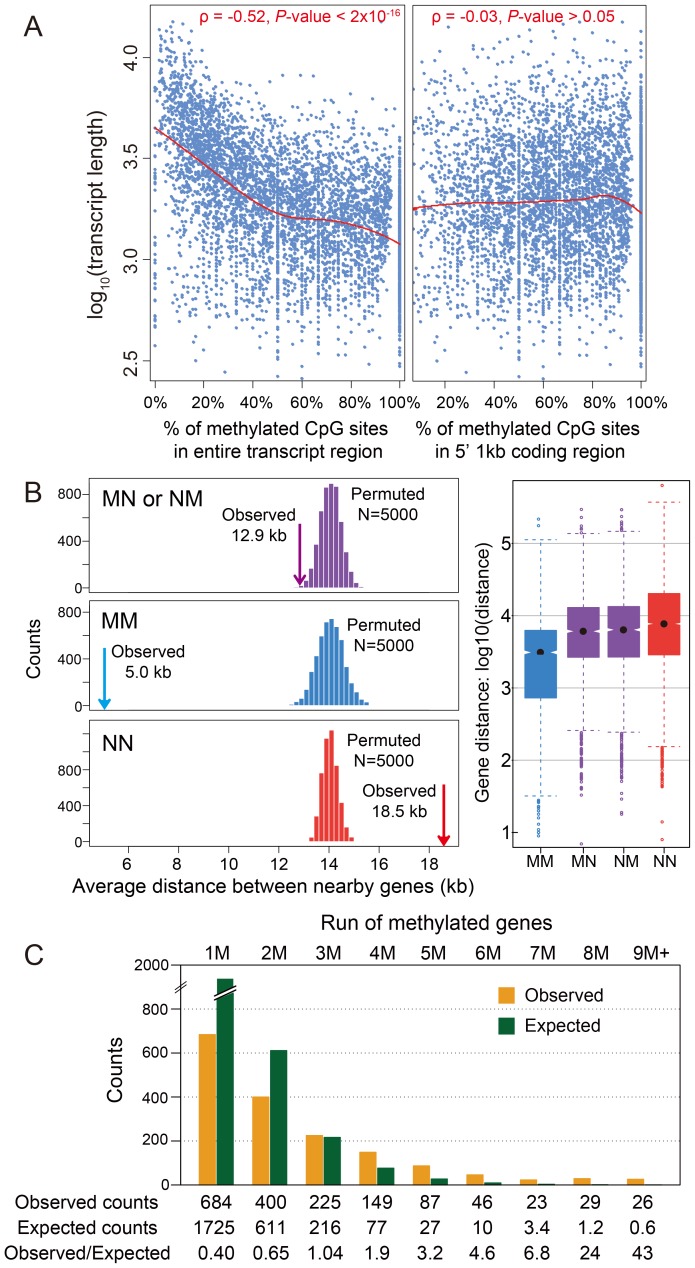
DNA methylation and gene length, exon number and gene locations. (A) Scatterplot for gene length (log_10_) and percentage of methylated CpG sites for methylation genes in the entire transcript region (left) and in 5′ 1 kbp coding region (right). The fitted lines using non-parametric local regression are shown in red. (B) Left: Distance between neighboring methylated genes (MM), non-methylated genes (NN) and methylated-non-methylated genes (MN or NM). The expected distributions for the three classes calculated by permuting the methylation status (N = 5,000) were plotted (MM: blue; NN: red; MN or NM: purple). The observed mean distance for each group was shown using arrows. Right: Distribution of the distance for the four classes (MM, NN, MN and NM). (C) Distribution of observed (orange) and expected (blue) counts for consecutive run of methylated genes. The expected counts were computed assuming the methylation status is randomly distributed.

In *Nasonia*, we initially observed a significant negative correlation (Spearman's rank correlation coefficient ρ = −0.52, *P*-value<2×10^−16^) between transcript length and the percentage of mCpGs in methylated genes, when the entire transcribed region is used (Figure 2A). However, the majority of DNA methylation is located in the first 1 kbp of the coding region (Figure 1E,G). When we examined the relationship using one kbp 5′ of coding regions (Figure 2A), the correlation disappeared (Spearman's ρ = −0.03 *P*-value>0.05). Therefore, the correlation with gene length is a byproduct of the 5′ bias to the distribution of methylation within genes.

#### A.5. Methylated genes are clustered in the *Nasonia* genome

Tandem methylated genes (MM) and non-methylated gene pairs (NN) are significantly over-represented compared to MN and NM pairs (Figure S11A; *P*-value<2.2×10^−16^, Chi-squared test), suggesting that methylated genes are clustered in the genome. The average distance between MM gene pairs (4.8 kbp) is much shorter than the expected distance under random distribution of methylated genes, and the distance for NN gene pairs is significantly longer (18.5 kbp) (Figure 2B; *P*-value<2.2×10^−16^, Mann-Whitney U Test). Moreover, consecutive runs of methylated genes (1M, 2M, 3M, etc.), are longer than expected by chance (Figure 2C; *P*-value<2.2×10^−16^, Chi-squared test), with a mean cluster size of 2.48. Neighboring genes within distances <1 kbp and coding on opposite strands (*i.e.*, in head-head and tail-tail formations) are enriched among methylated genes, and head-head formations comprise the highest fraction of methylation gene pairs (Figure S11B). This observed pattern of neighboring genes significantly sharing their methylation status (MM or NN) suggests potential co-regulation of methylation (Figure S11B). In conclusion, methylated genes tend to occur in small clusters within the genome.

#### A.6. The *Nasonia* genome lacks non-CpG DNA methylation

Non-CpG DNA methylation is rarely observed in the *Nasonia* genome; only 0.18% of Cs among the 60 million non-CpG positions with adequate read-depth are methylated (Table S5, Figure S12 and Text S4), which is less than the unconverted Cs in the lambda DNA used as a bisulfite control (Table S1). Therefore, many of these counts are likely experimental artifacts of bisulfite conversion or nucleotide mismatches in the reference genome (Table S6 and Figure S13). For example, of 28 top candidate non-CpG methylation sites with >30% unconverted Cs, eight (4 in top 10) are actually methylated at CpG sites, but were misidentified as non-CpG methylation due to sequence errors in the reference genome sequence (Table S6, Figure S13 and Text S4). Only one candidate non-CpG methylation site out of four examined was verified within the coding region of a gene (Figure S14 and Text S4).

### B. CpG methylation and gene expression

We next investigated associations between DNA methylation and gene expression, using a combination of RNA-seq data from adult females and genome-wide tiling microarray data from five different developmental stages: early embryo, late embryo, larva, pupa, and adult (Figure S15 and Dataset S1, See Materials and Methods). Here, we compare expression patterns across developmental stages, and also examine copies of duplicated genes that differ in their methylation status.

#### B.1. Methylated genes show higher median expression levels

**Figure 3:**
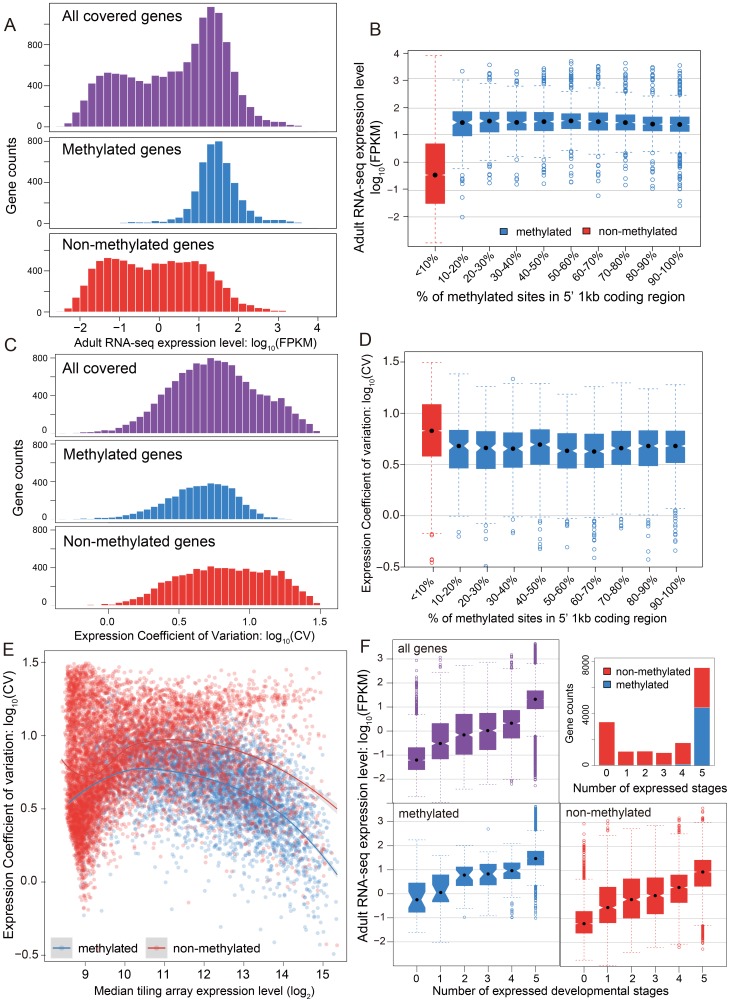
DNA methylation, gene expression and expression breadth. (A) Distribution of RNA-seq expression level (log_10_ FPKM) in adult female for methylated (blue), non-methylated (red) and all genes (purple). (B) Distribution of RNA-seq expression level (log_10_ FPKM) in adult female for groups of genes binned by percentage of methylated CpG sites in 5′ 1 kbp coding region. Red: non-methylation genes; blue: methylated genes. (C) Histograms for distribution of expression coefficient of variation (log_10_ expression CV) in five developmental stages (early embryo, late embryo, larvae, pupae and adult) for methylated (blue), non-methylated (red) and all genes (purple). (D) Distribution of expression breadth measurement (log_10_ expression CV) in six developmental stages for groups of genes binned by percentage of methylated CpG sites in 5′ 1 kbp coding region. Red: non-methylation genes; blue: methylated genes. (E) Scatterplot of expression breadth (log_2_ expression CV) on y-axis against median expression level (log_2_ signal intensity) in tiling array on x-axis, color-coded by adult female methylation status (blue: methylated genes; red: non-methylated genes). Fitted lines using non-parametric local regression are shown for methylated and non-methylated genes respectively. (F) Top right panel: Stacked barplot for expressed methylated and non-methylated genes with 0 to 6 expressed stages. Red: unmethylation genes; blue: methylated genes. Top left and bottom panel: boxplot for distribution of adult female RNA-seq expression level (log_10_ FPKM) for methylated (in blue), non-methylated (in red) and all genes (in purple) expressed in 0–5 developmental stages.

The relationship between methylation status and gene expression level was investigated using two different data sets – RNA-seq data for adult females and tiling microarray data for 5 different developmental stages (early embryo, late embryo, larva, pupa, adult). The RNA-seq results displayed a bimodal distribution of gene expression level in adult females (Figure 3A, *P*-value<2.2×10^−16^, Hartigans' dip test for unimodality) [44], [45]. Methylated genes have significantly higher expression level than non-methylated genes (*P*-value<2.2×10−^16^, Mann-Whitney U Test) and they showed markedly different patterns. The distribution of gene expression levels for methylated genes was unimodal (*P*-value = 1) and is generally composed of the higher expressed genes (Figure 3A), whereas the expression of the non-methylated genes is bimodal in distribution, with the moderately expressed set of genes overlapping with the expression levels observed from the methylated genes (Figure 3A, *P*-value = 0.03). Examination of the expression level for all genes reveals that non-methylated genes constitute the vast majority of low expressed genes. Furthermore, the non-methylated genes account for 99% of the genes that were not found to be expressed in the adult female RNA-seq data (FPKM <1).

In conclusion, DNA methylation in adult females is positively correlated with gene expression level in adult females, and most methylated genes are more highly expressed than typical for non-methylated genes (Figure S16). Nevertheless, methylation status is clearly not the only determinant for high gene expression, as many non-methylated genes also show high expression levels. The same general pattern was observed in tiling array data using median expression level across development (Figure S17). To examine whether there is a simple linear relationship between gene expression level and the percentage of mCpGs in methylated genes, we tested the difference of expression level for genes in different classes of mCpG percentage (Figure 3B). Among the methylated genes, we observed no positive correlation between methylation percentage and expression level (Spearman's ρ = −0.08). Therefore, gene expression is correlated with methylation status (methylated vs. non-methylated), but does not increase with increasing methylation level among methylated genes.

#### B.2. Methylated genes are constitutively expressed during development

Two metrics of gene expression change across development were calculated from the genome-wide tiling path microarray data: a coefficient of expression-level variation across the five developmental stages (expression CV), and the number of stages when gene expression is detected above baseline (see Methods & Materials).

While mean expression CV is lower in methylated (5.07) than non-methylated (6.07) genes, it is clear that both CV and median expression level across development covary (Figure 3C), which is confirmed in a logistic regression analysis (Text S5, Table S7 and S8). Because CV varies as a function of expression level, we examined the expression CV against the median expression level across development (Figure 3E). Excluding genes with very low expression (level<9) because there are too few methylated genes to make a proper comparison, we find that methylated genes have lower expression variation than non-methylated genes across a wide range of median expression levels. Dividing median expression level into three categories (9–11, 11–13, >13), methylated genes show significantly lower CV than do non-methylated genes for all categories (*P*-value<2.2×10^−16^ for all three categories, Mann-Whitney U Test; Table S9). The same trend is obtained when adult RNA-seq expression level is used in place of median expression across development (Figure S18, S19). Methylated genes have lower CVs across a broad range of median expression levels, indicating that they are expressed more evenly across development.

We next investigated the relationship between the methylation status of genes and the number of developmental stages with nonzero gene expression (see Materials and Methods). The majority of methylated genes (95%) are expressed broadly in all five developmental stages and less than 0.8% of the methylated genes have expression values below 9 in all five stages (Figure 3F). In contrast, only 28% of the non-methylated genes are expressed in all five stages, and 30% are absent (expression value <9) in all stages (Figure 3F). However, there is still a good proportion of non-methylated genes (3062 genes or 28%) that are expressed in all five stages, allowing us to compare expression breadth to median expression level across stages. Whereas it is not the case that all non-methylated genes are stage-specific, most methylated genes show broad expression across developmental stages, even when their median level of expression is relatively low.

The number of expressed stages is also correlated with the gene expression level. Genes present in more life stages tend to have a higher expression level (Figure S20). Taken together, these results strongly suggest that methylation is a general signal for constitutive expression of genes across development, and that this applies both to moderately expressed and highly expressed genes. Studies in *Apis* [28], [29], found that methylated genes are more broadly expressed across tissue/cell types. Here we show that methylated genes in *Nasonia* are more broadly expressed across developmental stages.

#### B.3. Methylated genes are enriched for basal cellular functions

We used blast2go (v2.6.0) to explore the enrichment of Gene Ontology (GO) term categories among methylated genes in *Nasonia*. This analysis reveals that methylated genes were generally enriched for basal cellular functions, such as translation, mRNA processing, and post-translational modifications (Table S10 and Figure S21). As the expression of methylated genes is distributed to the right of the median genome expression, we were concerned that the GO-term enrichment may be confounded by expression level differences between methylated and non-methylated genes. To adjust for this, we carried out a second analysis using gene lists restricted to low-, medium-, and high-expressed genes (See Materials and Methods). The GO-term enrichment among low-expressed methylated genes (Table S11) closely reflected those observed for all methylated genes (Table S10), however, for the medium- and high-expressed methylated genes (Table S12 and S13), cellular component terms became significantly enriched, specifically terms related to intracellular organelles. Both results are consistent with the conclusion that methylated genes in *Nasonia* are typically involved in cellular “house-keeping” functions, especially those involving translation, transcription and organelles.

#### B.4. Methylation is not required for differential splicing

**Figure 4:**
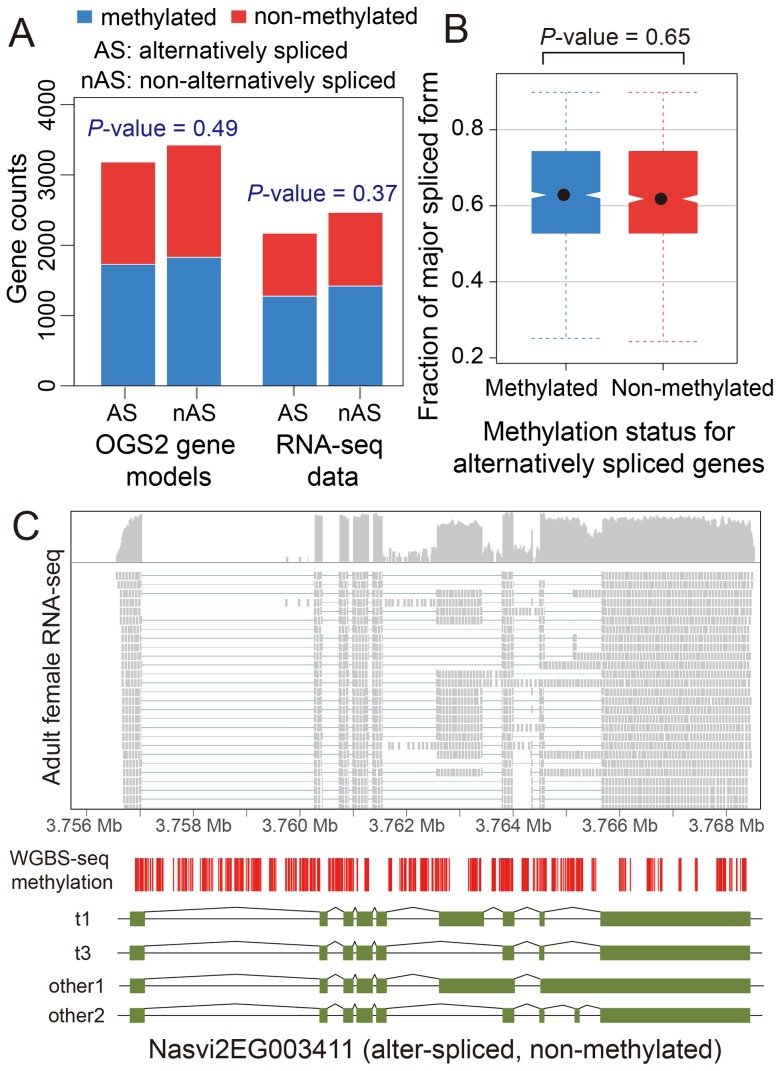
DNA methylation and alternative splicing. (A) Counts of alternatively spliced and non-alternatively spliced genes with different methylation status from OGS2 gene models (left) and RNA-seq data (right). AS: alternatively spliced; nAS: non-alternatively spliced. Methylated is shown in blue and non-methylated shown in red. (B) Distribution of fraction of major spliced forms for alternatively spliced methylated (blue) and non-methylated genes (red). (C) Gene expression, DNA methylation and alternative splicing profile for a non-methylated gene Nasvi2EG003411. Plotted at the top is the IGV browser screenshot showing adult female RNA-seq coverage (on log scale) and read alignments in the gene region. Plotted at the bottom are the CpG methylation profile at covered CpG sites from WGBS-seq data and the exon model of the alternatively spliced transcripts from OGS2 gene models. A vertical bar was drawn for each CpG at its position in the gene, color-coded by the methylation percentage in proportion to the bar length (blue: methylated Cs; red: non-methylated Cs). All 587 covered CpGs in the gene region were non-methylated. Two of the three OGS2 transcript variants, Nasvi2EG003411t1 (labelled as t1) and Nasvi2EG003411t3 (labelled as t3), were covered in the RNA-seq data with 47% and 41% of the transcript abundance, respectively. Two of the remaining minor transcript variants (other1 and other2) were also plotted.

We investigated patterns of DNA methylation in genes showing alternative splicing, to determine whether a signal of the alternative splice forms is apparent. We found no genome-wide correlation between methylation status and alternative splicing in adult females (Figure 4A–C and Figures S22 and S23, See Materials and Methods). Genes showing differential splicing are not more likely to be methylated than expected by chance (Figure 4A; *P*-value = 0.49, Chi-squared test), and there was no significant difference in the degree of alternative splicing between methylated and non-methylated genes, quantified by the fraction of major spliced forms (Figure 4B, *P*-value = 0.65, Kolmogorov-Smirnov test). In methylated genes with multiple methylated CpG clusters, we found in most cases that alternative exons within the first 1 kbp of the coding region do retain methylation (Text S6, Table S14 and Figure S24). However, as non-methylated genes also show extensive alternative splicing (Figure 4C), DNA methylation is clearly not required for differential splicing in *Nasonia*.

### C. Comparative Genomics of Methylated Genes

#### C.1. Methylated genes are more conserved in evolution

**Figure 5:**
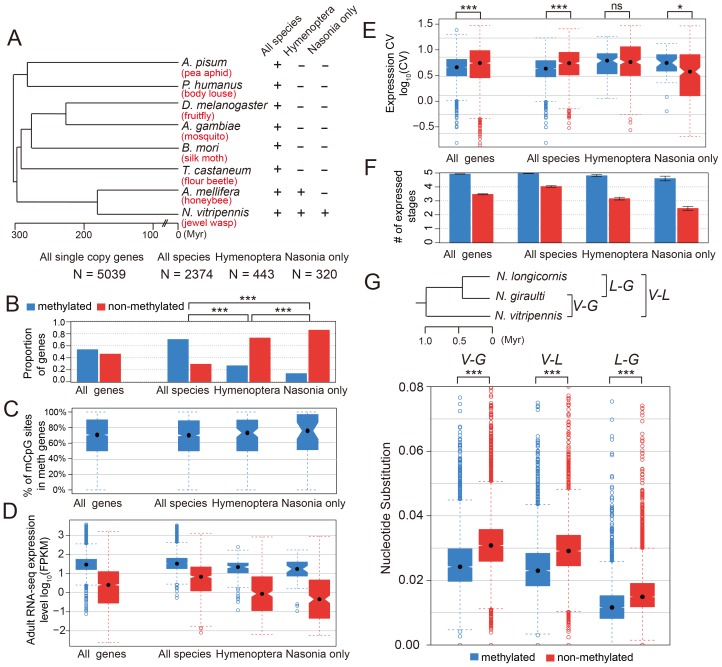
DNA methylation and gene conservation. (A) Phylogenetic tree of eight insect species: *Nasonia vitripennis*, *Apis mellifera*, *Tribolium castaneum*, *Bombyx mori*, *Anopheles gambiae*, *Drosophila melanogaster*, *Pediculus humanus* and *Acyrthosiphon pisum*. The methylation status and correlating factors were plotted in (B–F) for four groups of genes: all 5,039 *Nasonia* single-copy genes with one or zero ortholog in seven other insect species, 2,374 genes with one orthologs in all eight insect species, 443 genes with one orthologs in *Apis* and *Nasonia* but missing in other six species, and 320 genes present only in *Nasonia*. The *y*-axes plotted in (B–F) are (B): proportion of methylated (blue) and non-methylated genes (red); (C): percentage of methylated CpG sites in methylated genes; (D): adult RNA-seq expression levels (log_10_FPKM); (E): coefficient of variation of expression level in tiling array across six developmental stages; (F): number of expressed tissues. (G) Top: Phylogenetic tree of three *Nasonia* species: *N. longicornis* (L), *N. giraulti* (G) and *N. vitripennis* (V). Bottom: boxplots of nucleotide substitution rates between V–L, V–G and L–G.

To check the conservation status for methylated genes, we investigated 5,039 *Nasonia* single-copy genes covered in our WGBS-seq data that have either one or zero orthologs in each of seven other insect species (*Apis mellifera*, *Tribolium castaneum*, *Bombyx mori*, *Anopheles gambiae*, *Drosophila melanogaster*, *Pediculus humanus* and *Acyrthosiphon pisum*; see Materials and Methods). For these genes, we compared the methylation status in *Nasonia* to other factors among three gene conservation categories: genes present in single copy in all eight insect species (conserved genes), genes present in honeybee and *Nasonia* but not in other species (Hymenoptera-specific genes) and genes present only in *Nasonia* (*Nasonia*-specific genes) (Figure 5A). *Nasonia* methylated genes account for 71% of the genes present in all species, compared to 27% of Hymenoptera-specific genes and 14% of *Nasonia*-specific genes (Figure 5B). Therefore, *Nasonia* methylated genes were highly enriched in the conserved gene class (*P*-value<2×10^−16^, Chi-square test). The degree of methylation in methylated genes, measured by percentages of methylated CpG sites, was not significantly different among the three classes (*P*-value = 0.64, Kruskal-Wallis rank sum test) (Figure 5C).

Methylated genes have higher expression level (in *Nasonia*) in all three conservation classes (*P*-value<10^−10^, Mann-Whitney U Test), which is consistent with the methylation-expression correlation we observed (Figure 5D). In addition, Hymenoptera-specific and *Nasonia*-specific genes have lower expression levels compared to the conserved genes (*P*-value<2.2×10^−16^, Mann-Whitney U Test); however, the over-representation of non-methylated genes among them was not purely due to the expression difference. For all *Nasonia* single copy genes, non-methylated genes have higher expression variability across the five life stages (*P*-value<2.2×10^−16^, Mann-Whitney U Test, one-side). This is also true for the conserved genes (the “all species” category; *P*-value = 9.1×10^−16^, Mann-Whitney U Test, one-side). However, there is no significant difference in expression variability for hymenopteran-specific genes (*P*-value = 0.17, Mann-Whitney U Test, one-side), while *Nasonia*-specific genes showed the opposite pattern with (*P*-value = 0.018, Mann-Whitney U Test, one side) (Figure 5E). The reverse pattern found in *Nasonia*-specific methylated genes is relatively weak, although statistically significant.

Because expression level of non-methylated genes declines with decreasing conservation (Figure 5D) and CV co-varies with expression level (Figure 3E), CV is not the best index of expression breadth when comparing methylated and non-methylated genes of different conservation levels. We therefore examined how broadly genes are expressed across development for different conservation levels and methylation status (Figure 5F). Methylated genes are expressed more broadly than non-methylated genes for all three conservation categories. Conserved non-methylated genes (i.e. present in all species) are expressed in 4 stages on average, but the number dropped to 3.1 for hymenopteran-specific genes and further dropped to 2.5 for *Nasonia*-specific genes; methylated genes showed a much less dramatic decline, from 4.97 for all species to 4.81 for hymenoptera-specific genes and 4.60 for *Nasonia*-specific genes (Figure 5F). The median values were significantly different for all three categories (Table S15). These results show that methylated genes are more broadly expressed than non-methylated genes across conservation categories, and therefore indicate that even more recently evolved methylated genes acquire broader constitutive expression across development than comparable non-methylated genes.

#### C.2. There is significant conservation of gene methylation status between *Nasonia* and *Apis*

We next compared patterns between *Nasonia* and *Apis*, each being a representative of two major groups of Hymenoptera that have diverged approximately 180 MYA [34]. The honeybee (*Apis*) methylome data were available in the literature [15]. There were 3,206 *Nasonia*-*Apis* 1∶1 orthologous gene-pairs with methylation status called in both species. Of these, 71.9% are methylated in *Nasonia* compared to 47.7% in *Apis*. Note that the calling of methylation status is different between the *Nasonia* and *Apis*, as data on the distribution of methylated sites within genes (*i.e.* 5′ to 3′) was not available to us for *Apis* (see Materials and Methods). Despite these methodological limitations, there is a strong positive correlation in gene methylation status between *Apis* and *Nasonia* (P-value<2.2×10^−16^, Chi-squared test), with 42.2% of genes methylated in both species, compared to an expected 34.3%. Furthermore, when we calculated the % of methylated CpGs across the entire gene (the same as done for *Apis*), only 5% of the non-methylated genes changed status to methylated, and the finding of general conservation of methylation status was still found. These findings, based on genome-wide methylation criteria, are consistent with an earlier study showing conservation in gene methylation between *Nasonia* and *Apis*, based on inferred methylation from CpG O/E [20].

#### C.3. Methylated genes evolve more slowly within the *Nasonia* clade

We also examined methylation status and gene conservation at a shorter evolutionary time scale among *Nasonia* species. The nucleotide substitution rates in ∼7,000 genes were compared across three *Nasonia* species: *N. longicornis*, *N. giraulti* and *N. vitripennis* (see Materials and Methods). In all comparisons, methylated genes have lower nucleotide substitution rates (*P*-value<2.2×10^−16^, Mann-Whitney U Test) (Figure 5H).

#### C.4. *Apis* to *Nasonia* differences in methylation associate with gene ontology

To investigate whether GO-categories of methylated genes are conserved between *Apis* and *Nasonia*, we identified all 1-to-1 orthologs between *Nasonia* and *Apis* for which we had confident methylation status calls (3206 loci), and tested for enrichment of GO-terms where methylation status was either conserved or diverged between these two hymenopteran species. Once again, the most significantly enriched GO-terms for genes methylated in both *Nasonia* and *Apis* (1354 loci) are in categories associated with basal cellular processes such as metabolism and organelle function (Table S16). Next we restricted our lists to genes showing lineage-specific methylation within either *Nasonia* or *Apis*. For genes with methylation only in *Nasonia* (682 loci), ribonucleoprotein complex was enriched at the 5% FDR cutoff level (Table S17). No GO term enrichment was observed at this cutoff for genes methylated in *Apis* only (176 loci); however, processes related to sensory system were enriched at a more permissive FDR cutoff (results not shown).

#### C.5. When duplicated genes lose methylation, they evolve more quickly and become more developmentally specialized

**Figure 6:**
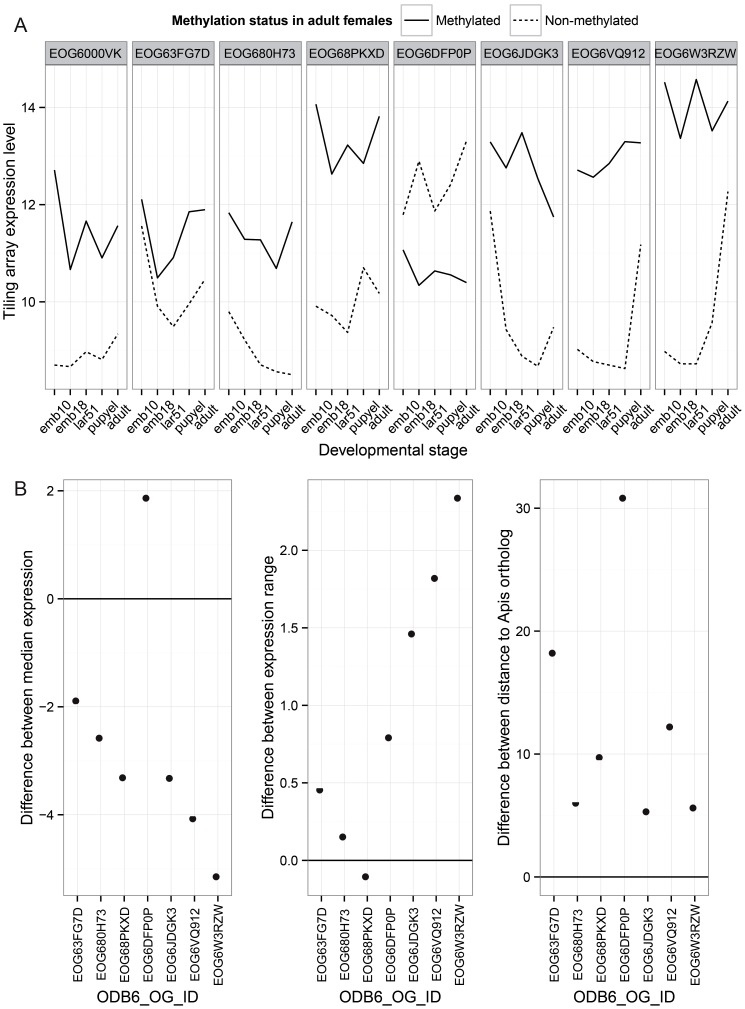
Paralog analysis. Differences between two paralogs that have changed in methylation status in the Nasonia lineage are shown. (A) Comparisons of expression pattern across developmental stages for duplicated genes in the *Nasonia* lineage where one gene is methylated (M) and the other lost methylation (N). These genes have 1∶1 orthologs in other hymenopteran species, and the ortholog is methylated in *Apis*. (B) Those paralogs that lost methylation show significant reductions in median expression level across development relative to the M paralog (N–M), significant increases in the range of expression level (N–M), and significantly greater divergence from the *Apis* ortholog (N–M).

Finally, we investigated the patterns of evolution of genes that have undergone gene duplications in the clade leading to *Nasonia*. A total of 145 orthologous gene sets were identified that are present in a single copy in all other Hymenoptera (OrthoDB, 13 taxa examined) [46], but which have undergone a gene duplication in the *Nasonia* clade. Methylation status in both *Nasonia* duplicates and the *Apis* paralog was available for 33 of these. In 9 (27%), the *Apis* ortholog and both *Nasonia* paralogs are methylated. In 8 cases, one of the *Nasonia* paralogs was non-methylated (N) whereas the other paralog and *Apis* gene was methylated (M) (Table S18). Those 8 gene pairs are present in a single copy across all Hymenoptera, with the exception of *Nasonia*. We therefore infer that they underwent a lineage-specific duplication, followed by loss of methylation in one of the paralogs. We examined gene expression and rates of divergence in each M to N conversion, using *Apis* as the outgroup. Despite the small sample size, several striking patterns are observed. First, in 7 of 8 cases, the N paralog has lower median expression across developmental stages than does the M (*P*-value = 0.016, WMSRT). In one case, the N paralog expression was close to the minimum detection level at all five developmental stages in the tiling array data, and we therefore excluded it as possible pseudogene. In the remaining 7 cases, the N paralogs showed significantly lower median expression levels (Figure 6A; *P*-value = 0.031, WMSRT). As is apparent in Figure 6, whereas the median expression is lower for the N genes, they show a greater variation of expression (*P*-value = 0.016, WMSRT in coefficient of variation) and greater maximum expression difference than do their M paralogs (*P*-value = 0.031, WMSRT), indicating that the N genes have maintained or evolved high expression within certain life stages. Finally, the N genes have significantly longer branch lengths (Figure 6B, *P*-value = 0.016, WMSRT) than their M paralogs, indicating more rapid evolution. These results suggest that loss of methylation status following gene duplication correlates with loss of constitutive expression across developmental stages, and possibly increased evolution and specialization of the duplicated gene.

## Discussion

In this study, we profiled the genome-wide methylation at base-pair resolution in *Nasonia* and found several striking features. First, 1.6% of covered CpG sites are methylated in the *Nasonia* genome, and the methylated CpGs are clustered along the genome. As found in several other invertebrates [15], [18], [19], DNA methylation is located mainly in the gene bodies in *Nasonia*, with coding genes falling into two distinct groups: around 30% of genes are methylated and show strong CpG methylation in 5′ exons, while DNA methylation is largely absent in the remaining genes. To compare the global methylation level across hymenopteran species, we calculated the percentage of methylated CpGs (mC/C) in *Nasonia*, *Apis* and ants (Text S7). Although it is difficult to compare genome-wide methylation levels due to differences in methodology, it appears that *Nasonia* (1.6%) has a higher overall methylation level than is found in honeybees (0.8%) or ants (1.05% in *Camponotus* and 0.68% in *Harpegnathos*).

Unlike mammals, where methylation is associated with suppression of transposon gene expression, with rare exceptions TEs are not methylated in *Nasonia*. The finding is in concordance with honeybee TE methylation profile [19], and suggests that DNA methylation is not required for TE repression in insects. In ants, TE methylation is at the genomic background level, but certain types of TE are hypermethylated and the pattern is species-specific [18]. In our data, we found five retrotransposon families with >5% methylation across CpG sites. The top three methylated TE types (SNAKEHEAD, GYPSY and SPRINGER) are highly expressed in the adult female RNA-seq data (Table S4), suggesting that DNA methylation may actually enhance expression of these elements. We do not know how this is accomplished, but it is possible that certain TEs may contain (or land near) sequence signals that promote DNA methylation. But globally the vast majority of TEs show no methylation in *Nasonia*.

Close examination of methylation in coding genes revealed a striking matching of methylation with the transcription unit. Methylation is low in 5′ UTR and increases rapidly near the transcription start site. Methylation is then consistently higher on exons and decreases significantly on introns, resulting in a clear delineation of exon-intron boundaries by methylation “tagging”. Finally, at least for methylated genes <1 kbp in length, methylation also declines significantly in the 3′UTR (after the stop codon). These patterns across the gene region suggest that DNA methylation provides “tags” that mark exons and targets introns for excision during transcription, but also that mark location of translational start and stop, even though translation occurs in the cytoplasm and is not directly associated with the DNA. If methylation affects the rate of transcription, then it is possible that methylation-induced transcriptional pausing at the exon-intron boundary could play a role in splicing [47]. However, how would the DNA methylation signal result in tagging of mature mRNA to demarcate translational initiation and termination? One possibility is through directing mRNA base modifications. For example, in mammals methylation of the N6 position of adenosine (m6A) has been shown to accumulate at stop codons and 3′UTR [48], suggesting a possible signal for translation termination.

It has been hypothesized that in insects DNA methylation regulates alternative splicing [19]; however, a direct causal relationship between methylation and differential splicing remains unsubstantiated. In *Nasonia*, we found no global correlation between methylation status and alternative splicing, although methylation changes across exon/intron boundaries suggested a potential link between DNA methylation and splicing. We should emphasize that DNA methylation is not required for either intron splicing or coding region demarcation, as non-methylated genes show both. Nevertheless, it is possible that methylation expedites these signals for a subset of methylated house-keeping genes, which we have shown to be expressed constitutively and at higher levels. Investigating these mechanisms is an interesting avenue for future research.

In *Nasonia*, the exon-intron pattern is augmented by a strong 5′ bias in level of methylation. The majority of DNA methylation was within the first 1 kbp coding exons and clearly drops beyond that in *Nasonia*, although an exon-intron distinction is still discernible in larger genes. A similar 5′-biased DNA methylation pattern has been observed in ants [18]. Studies in honeybee have reported a negative correlation between gene length and methylation status [49] and we observed the same pattern in *Nasonia* when the methylation percentage across the entire gene was used; however, this pattern disappears in *Nasonia* when the score of methylation level is restricted to the first 1 kbp of the coding region. We found little evidence for non-CpG methylation in *Nasonia*, but were able to confirm a single case. Therefore, non-CpG methylation is present, but it is extremely rare in *Nasonia*. Most candidate non-CpG methylation sites were located in genes nested in CpG methylation clusters. These findings suggest that non-CpG methylation may result from the inaccurate methylation at non-CpG sites by the CpG methylation machinery. It may strengthen the CpG methylation cluster, but the biological significance remains an open question.

In mammals, DNA methylation at promoter regions is often associated with suppression of gene expression [50], [51]. However, in insects, DNA methylation has been shown to be positively correlated with expression level in silkworm and ants [18], [22]. Here, we also observed a strong positive correlation between methylation and gene expression level; however, methylation is more strongly associated with constitutive expression across development independent of expression level. The distribution of expression levels for methylated genes is unimodal, matching the high expression class. Non-methylated genes show a bimodal distribution, with a mixture of both low and moderate expression, indicating DNA methylation is not the only factor affecting expression level. Other epigenetic marks such as histone modifications are likely to play a role in expression differences among non-methylated genes.

By comparing gene expression levels across five developmental stages, we found that methylated genes show more even expression across stages, and this pattern applies to both highly- and moderately-expressed methylated genes. The finding complements studies in honeybee, which found methylated genes to be expressed across multiple tissues, whereas non-methylated genes showed a more spatially restricted expression pattern [28], [29]. In both cases, methylation appears to be more prevalent in genes that are constitutively expressed across development and tissue types. GO-term analysis showed that methylated genes in *Nasonia* are enriched for genes with housekeeping functions, as observed in honeybee and ants [18], [19], [21]. Furthermore, genes methylated in *Nasonia* tend to be more evolutionarily conserved, as also found in recent studies in ants and other invertebrates [27], [52]. Housekeeping genes tend to be expressed in most tissue and cell types, which may explain the low expression variability for methylated genes across stages.

Further support for the role of methylation in constitutive expression of genes comes from the study of duplicated genes that have lost methylation relative to their paralog in the *Nasonia* lineage. Comparing non-methylated and methylated paralogs reveals both a marked median reduction in expression level, and evolution toward more developmental stage-specific expression patterns in the non-methylated genes. Functional category enrichment analysis showed that methylated genes are enriched for basic cellular functions, such as transcription and translation, as also found in honeybee and ants [18], [19], [21]. Our comparative genomic analysis also shows that many genes have maintained their methylation status across the long evolutionary time scale from *Apis* to *Nasonia*. This probably reflects the role of methylation in constitutive expression of basal housekeeping genes. We also find that methylated genes are enriched among the class of genes that are conserved among insects, while non-methylated genes are enriched among Hymenoptera-specific and *Nasonia*-specific genes. Nevertheless, methylated genes are expressed more broadly across development than are non-methylated genes for each of these conservation categories. Even the more recently evolved “*Nasonia*-specific” methylated genes show broad expression across developmental stages (median 4.60), considerably greater than for non-methylated genes (median 2.5). This suggests that broader constitutive expression is a hallmark of methylated genes whether they are conserved or recently evolved.

Bisulfite sequencing and expression profiling in our study were done on whole insects. Therefore, it could be argued that the correlation between methylation status and expression level occurs because genes that are methylated in more tissues show both higher levels of methylation and higher expression. In other words, tissue specific changes in methylation regulate tissue-specific gene expression, and this creates a correlation between methylation status and gene expression in whole animals. Although a possibility, we found that among methylated genes there is no correlation between level of methylation and level of expression (Figure 3B), which would be expected if the proportion of tissues in which the gene is methylated was driving the pattern. Future work will help resolve whether some genes are being differentially regulated by changes in methylation status. However, it appears that in general DNA methylation is a hallmark of genes that are constitutively “turned on”, at least across developmental stages.

In some eusocial organisms such as honeybee and ants, DNA methylation was shown to be related to caste determination [18], [19], [53]. In *Nasonia*, we have no evidence as yet that changes in methylation regulate specific developmental programs. In contrast, the general data reported above suggest that its primary role is in maintaining constitutive (and perhaps higher) expression of a subset of important cellular “house-keeping” genes, whereas non-methylated genes are more involved in stage-specific differences in expression.

Investigating the role of methylation in epigenetic processes (*e.g*. sexual differentiation, tissue-specific gene expression) will motivate the future study of establishment, maintenance, epigenetic reprogramming and interactors of DNA methylation in *Nasonia* and other insects. Comparison among closely related *Nasonia* also provides the opportunity to study the microevolution of DNA methylation. In addition, the ability to genetically dissect species differences in *Nasonia* through inter-fertile crosses [41], [54], [55] could provide tools for the genetic investigation of *cis*-regulatory mechanisms of DNA methylation.

## Materials and Methods

### Sample collection, genomic DNA and total RNA extraction

Genomic DNA samples were extracted from a pool of 50 24 h adult females from the standard *N. vitripennis* strain AsymCX using DNeasy Blood & Tissue Kit (Qiagen, CA). This is the same strain used for the *Nasonia* genome project [34] and is cured of the intracellular bacterium *Wolbachia*.

For RNA-seq, total RNA samples were extracted from adult females ∼24 h following eclosion from pupation, using RNeasy Plus mini kit (Qiagen, CA) following the manufacturer's protocol. The DNA, RNA concentration and the A260 nm/A280 nm absorption ratios were measured by NanoDrop ND-1000 Spectrophotometer (Thermo Scientific, DE) to assess quality. RNA integrity was checked using the Agilent 2100 Bioanalyzer (Agilent Technologies, CA). All of the samples had a RIN (RNA integrity number) in the range 9.8–10.0 (RIN_max_ = 10.0).

For tiling microarrays, total RNA was extracted from samples of 5 different life stages, 0–10 h embryos, 18–30 h embryos, 51–57 h larvae, day yellow pupae (little to no red eye pigment), and 1 day post eclosion adults. To generate the samples, mated females were first singly given two *Sarcophaga bullata* hosts for 48 h and then given one host for 6 hours, with access to the host restricted to one end for ease of embryo collection. Embryos or larvae were then collected from the hosts. Under this experimental design, females typically produce 85–95% female offspring, and these percentages were confirmed using control hosts where the offspring were permitted to complete development. Therefore, the wasps from these samples are predominantly female, although individual embryos or larvae were not sexed. For pupal collections, hosts were opened and female pupae from the “yellow pupal” stage were collected. Adult females were collected for RNA extraction ∼24 h after eclosion from the pupal stage. Six replicates per sample were used, averaging 400 individuals per replicate for embryos, 300 for larvae, 20 for pupae and 20 for adults. Samples were extracted in Trizol (Invitrogen, cat#15596-026) and then sent to the Indiana University Center for Genomics and Bioinformatics for sample preparation and tiling microarray analysis using previously published methods.

### WGBS-seq and mRNA-seq library preparation and Illumina sequencing

20 µg of female *Nasonia* genomic DNA and 5 µg non-methylated control lambda DNA (catalog #: D1521, Promega, WI) were sheared by Covaris S2 system (Covaris, MA) for 480 second with 10% duty cycle, level 5 intensity and 200 cycles per burst. The DNA fragments were purified with Zymo DNA Clean & Concentrator-5 columns (Zymo Research, CA), size-selected for 130–180 bp with E-Gel system (Life technologies, CA) and QIAquick Gel Extraction Kit (Qiagen, CA), end-repaired with NEBnext end repair module and NEBnext dA tailing module (New England Biolabs Inc., MA), ligated with Illumina methylated PE adapter oligo (part #1005560, Illumina, CA) and then purified with Agencourt AMPure XP beads (Beckman Coulter, CA). We performed bisulfite conversion on purified *Nasonia* adult DNA and lambda control DNA using Qiagen EpiTect Bisulfite kit with 2× bisulfite conversion cycles to improve the conversion efficiency and then purified the elute by AMPure XP beads. The purified converted DNA was amplified with PfuTurbo Cx Hotstart DNA Polymerase (Agilent Technologies, CA) using 15 cycles. The final libraries were purified again using AMPure XP beads and the library concentration was measured by Qubit (Life technologies, CA). The library size distribution was checked by Agilent 2100 Bioanalyzer (Agilent Technologies, CA).

We mixed 0.5% of the lambda control DNA library in the *Nasonia* DNA WGBS-seq library, and performed Illumina short-read sequencing in one 84 bp lane on Genome Analyzer IIx (GAIIx) and one 101 bp paired-end lane on HiSeq2000 instrument. Image analysis and base calling were performed by the Illumina instrument software. In total, we obtained 27,766,713 reads from the GAIIx lane and 89,739,445 reads from the HiSeq2000 lane. Illumina WGBS-seq data have been deposited in GEO under accession no. GSE43423.

The mRNA-Seq library was made from 3.5 µg total RNA samples from 24 h adult females, using TruSeq RNA Sample Preparation Kits v2 (Illumina Inc., CA). The library was sequenced on an Illumina HiSeq2000 instrument and we obtained 65,334,896 reads. IIlumina RNA-seq data in this study have been deposited in GEO under accession no. GSE43422.

### WGBS-seq and mRNA-seq read alignments and data analysis

The Illumina quality score and nucleotide distribution were checked by the FASTX toolkits (http://hannonlab.cshl.edu/fastx_toolkit/index.html). The adapter sequences were removed from the raw reads by custom scripts (0.7% in GAIIx lane and 0.9% in HiSeq lane). To include only high quality bases in our analysis, the sequence reads were trimmed to 75 bp. After trimming, the GAIIx and HiSeq (read 1 only) data gave us 8.75 Gbp of sequences or 25× coverage of the haploid genome, assuming 350 Mbp genome size.

We first aligned the reads to the plus and minus strands of non-methylated lambda genome (NCBI reference sequence NC_001416) with all Cs converted to Ts, using BWA with 4 mismatches [56]. A total of 746,736 (0.64%) reads were uniquely mapped to the lambda genome without indels, resulting 1155× coverage of lambda genome. We estimated the unconverted Cs to be 0.31% by subtracting the background T→C sequence error from the remaining unconverted Cs, therefore the final bisulfite conversion efficiency, at 99.69%, was ideal for downstream analysis. The Illumina sequencing error rates for each type of nucleotide in the GAIIx and HiSeq lane were also estimated from the lambda control alignments (Table S1).

From the *N. vitripennis* reference scaffolds [34], we built C→T converted reference genomes for both the Watson (+) and Crick (−) strand separately, with all Cs in CpGs context remains Cs (meth_genome) and all CpG Cs converted to Ts (unmeth_genome). The rest Illumina sequencing reads were aligned to the converted genomes with BWA [56] with a maximum of 4 mismatches, and summarized in a single BAM file (Figure S1). We tested 4, 6, 8 and 10 mismatches and found 4 mismatches will give the best mapping percentage without ambiguity due to reduced genome complexity after bisulfite conversion. ∼80% of the reads could be mapped to the converted *Nasonia* reference genome. To get accurate methylation estimation, we only used uniquely mapped reads without any indel (60% of total reads) for the methylation quantification. CpG methylation percentages were estimated from the proportion of remaining Cs in CpG context (Table S2). Non-CpG methylation was also quantified (Table S5).

We aligned adult female RNA-seq reads to the *Nasonia* reference scaffolds using TopHat v1.4.1 [57] with a maximum of three mismatches. 94% of the reads were uniquely mapped to the genome. Total expression level (FPKM: Fragments Per Kilobase-pair of exon Model) was calculated using Cufflinks v1.3.0 [58] based on all mapped reads from the TopHat alignments. The multiple mapped reads were weighted using the “-u” parameter in Cufflinks. The RNA-seq alignments were viewed in the IGV browser [59], [60].

### CpG methylation quantification and gene methylation analysis

Among the 14,024,488 CpG sites in *Nasonia* haploid genome, we covered >90% with 2 or more uniquely aligned reads and >55% with 10 or more reads. The average coverage at CpG sites is 16.2× (Figure S2). To obtain accurate quantification of the methylation percentages, we only included ∼8 M CpGs sites with 10 or more coverage (covered CpGs). To quantify the CpG methylation levels, we used two metrics: percentage of methylated CpGs (percentage of mCpGs) and average methylation percentage in covered CpGs (methylation percentage). Methylated CpGs (mCpGs) are defined as CpG sites with >10% methylated Cs and ≥10 coverage. This definition requires at least two unconverted C containing reads to call a site methylated, therefore a single T→C Illumina sequence error will not results a spurious methylated site.

Methylation percentage is the average methylated percentage over all CpGs in a particular region, which is the total number of unconverted Cs divided by the total number of reads at CpG sites. The methylated CpG sites were annotated using both the *Nasonia* OGS1.2 (official gene set) and OGS2 gene models [42]. OGS2 gene models incorporated both whole genome tiling expression array and RNA-seq data from multiple tissues at multiple developmental time points, proving high quality support for 5′- and 3′-UTR annotation. Among the 14,024,488 CpGs, 1,159,303 were located in overlapped gene models and were excluded from the analysis. To determine the gene methylation status, we calculated the percentage of mCpG among the covered CpG sites (depth ≥10) in both the first 1 kbp coding region and in the entire transcript region. Since the majority of the mCpGs are located in the first 1 kbp coding region and the methylation level is under the UTR level beyond 2 kbp (Figure 1G), long genes with heavy methylation at the beginning will be averaged out if the entire transcript length was used. Therefore, we inferred the gene methylation status using the percentage of mCpG in the first 1 kbp coding region. Because single or sparse mCpG could be spuriously generated by T→C sequencing error, local incomplete bisulfite conversion or alignment problems, we applied arbitrary cut-off and genes with at least four covered CpGs and >10% mCpG in the first 1 kbp coding region are classified as methylated genes; genes with ≤10% mCpG are defined as non-methylated genes.

To quantify the DNA methylation in repetitive elements and retrotransposons, we built a non-redundant repeat sequence database for the repeat library and retroid elements annotation from the *Nasonia* genome project [34]. From the 1195 sequences in the repeat library, 763 that are >100 bp in length and contain 4 or more CpGs were kept. Simple repeats and STRs were excluded from the analysis. The longest element in each of the 76 retroid families was included in repetitive elements database. We aligned the unmapped and non-uniquely mapped WGBS-seq reads to the database, and quantified the methylation percentage at CpG positions. Elements with average read depth four or more were included in the analysis.

### Characterizing the CpG islands and methylated CpG clusters in the *Nasonia* genome

To search for mammalian type CpG islands (CGIs) in the *Nasonia* genome, we ran predictions of CGIs in the *Nasonia* genome using the same criteria as in mammals [10]: GC percent >50%, CpG O/E (observed/expected CpGs) ratio >0.6, and greater than 200 bp in length. 9,265 CGIs were found in the *Nasonia* genome. We define methylated CpG clusters (mCpGCLs) as regions with >80% methylated CpGs and >40% average methylation percentage, and we found 5,440 mCpGCLs in the *Nasonia* genome.

### Analysis of clustering of methylated genes in the *Nasonia* genome

To determine whether the methylated genes are clustered or randomly distributed in the *Nasonia* genome, we analyzed the frequency and distance between neighboring gene pairs (MM: methylated-methylated; MN: methylated-nonmethylated; NM: nonmethylated-methylated; NN: nonmethylated-nonmethylated), as well as the consecutive runs of methylated genes. Scaffold rather than the chromosomal locations were used for the analysis because neighboring genes on two different scaffolds are not in proximity. To eliminate the effect of short scaffolds with few genes in them, only the top 100 largest scaffolds were included for the analysis, containing 11,683 genes with methylation status.

### Validation of methylated and non-methylated genes using cloning and sequencing method

To confirm methylation status of individual genes, DNA from 20 pooled 24–27 h virgin *Nasonia vitripennis* (strain Asymcx) females was extracted using the Qiagen DNeasy Blood and Tissue Kit (Cat No. 69504). The bisulfite conversion was performed by the Qiagen EpiTect Bisulfite Kit (Cat No. 59104) with 1.5 µg of starting DNA. Bisulfite PCR primers for six selected genes were designed using Methyl Primer Express software v1.0 (Applied Biosystems by Life Technologies, CA). The amplified PCR product was gel purified and cloned using Promega pGEM-T Easy Vector System II (Cat No. A1380). Direct PCR from the *E. coli* “white” colonies with T7 and SP6 primers was used to select colonies with the right insert size, which were then inoculated in LB broth with ampicillin and the plasmid was extracted using the QIAprep Miniprep kit (Cat No. 27104). Prism BigDye Terminator v3.1 Cycle Sequencing Ready Run Kit (Applied Biosystems) was used to prepare the products for sequencing. BigDye clean-up was completed using ABgene Dye Terminator Removal Kit (Cat No. AB-0943). Sequencing was completed at the Function Genomic Center at the University of Rochester.

### Tiling microarray sample preparation

We used NimbleGen high-density 2 (HD2) arrays for transcriptome investigations. The custom 4-array (chip) set consisted of 8.4 million isothermal long-oligonucleotide probes that are 50–60 nt in length and that span the *Nasonia* genome sequence at overlapping intervals of 33 bp, on average. Each slide contained 27,000 Markov model random probes that are not represented in the genome for setting background level thresholds. All probes were designed using NimbleGen's ArrayScribe software and the quality assurance tests of the probes were conducted using Indiana University's Centre for Genomics and Bioinformatics in-house algorithms. Signal to background ratios were determined by first calling probes that fluoresced at intensities greater than 99% of the random probes' signal intensities; therefore only 1% of fluorescing probes are likely to be false positives. The arrays reliably produced high signal to background ratios; log2 ratios of eight were observed for signal over background.

We conducted three replicates each using RNA from independent biological extractions of female early embryo (0–10 h), late embryo (18–30 h), 1st instar larvae, and pupae. Additional experiments were performed comparing transcription in testis and the female reproductive tract. Samples were prepared at 25°C as follows: Approximately 100 *N. vitripennis* (AsymCX) virgins were collected as black pupae. After eclosion, females were provided with males and allowed to mate overnight. Females were initially provisions 15–20 *Sarchophaga bullata* hosts in groups of 20 females for 24 h to induce production of eggs. The hosts were then removed and females were left overnight (∼18 h). Mated females produced 85% female progeny under the design used here, and therefore the embryo and larval collections are predominantly female offspring. To collect embryos, individual females were given access to a host at one end (to restrict the oviposition site) and allowed to lay eggs for 6–10 h before being removed. Embryos were then harvested immediately (early embryos), 18 h later (late embryos), or 51 h later (1st instar larvae). All embryos and larvae were collected in an RNase free environment. The host was cracked open and the “cap” removed to expose the embryo. Dissecting needles were used to gently scrape embryos from the surface of the host and transfer them into a 1.5 ml tube pre-chilled on dry ice. Samples were stored at −80°C. If at any time the host was punctured or embryos were exposed to host hemolymph, they were discarded. Estimates of the number of embryos per replicate (three per life stage/sex) were recorded; early embryos ranged from 300–900, late embryo 140–500, 1st instar larvae 245–520. Since sex cannot be determined at larval stage, some of the mated female hostings were allowed to mature to adulthood then males and females were counted to determine the sex ratio. Early larvae showed an average of 82.9% females and late larvae had an average of 84.2%. Pupae collections were made among the progeny of mated females provided with hosts for 48 hrs. They were sorted by sex and stage (early yellow, red-eye, half black, and black pupae). Equal numbers (*S2*0) of pupae from each stage were then pooled prior to RNA extraction. Female reproductive tracts (30 per replicate) were removed from 1–3 days post eclosion virgin females and transferred to a tube on dry ice prior to RNA extraction.

Tissue was disrupted and homogenized using Trizol reagent (Invitrogen), and extracted RNA was purified using the Qiagen RNeasy protocol with optimal, on column DNase treatment from specific tissues. Beginning with at least 0.5 µg of total RNA (for early to late embryo) or at least 1.0 µg (for other tissue types), a single round of amplification using MessageAmp II aRNA kit (Ambion) produced between 30 and 45 µg of cRNA for embryo RNA and greater than 100 µg for all other tissue types. Starting with 10 µg of cRNA, double strand cDNA synthesis was carried out using the Invitrogen SuperScript Double-Stranded cDNA Synthesis kit using random hexamer primer followed by DNA labeling using 1 O.D. CY-labeled random nonomer primer and 100 U Klenow fragment (3>5 exo) per 1 µg double-stranded cDNA. The use of random primers ensured that all transcripts hybridize to the array, which contains probes designed solely from a single strand of the DNA sequence. Both sexes for each tissue type were alternatively labelled and a dye-swap was included among the replicate experiments. Dual-color hybridization, post-hybridization washing and scanning were done according to the manufacturer's instructions. Images were acquired using a GenePix 4200A scanner with GenePix 6.0 software. The data from these arrays were extracted using the software NimbleScan 2.4 (Roche NimbleGen). The normalized tiling array data can be found in Dataset S1.

### Tiling array data analysis

The data analysis was performed using the statistical software package R (http://www.r-project.org/) and Bioconductor (http://www.bioconductor.org/) [61]. The signal distributions across chips, samples and replicates were adjusted to be equal according to the mean fluorescence of the random probes on each array. All probes including random probes were quantile normalized across replicates. Scores were assigned for each predicted OGS v2 gene, for each sample, based on the median log2 fluorescence over background intensity of probes falling within the boundaries of the largest gene transcript. The genes were deemed to be transcribed only when greater than ½ or their tiled length was expressed. On average, the 23,161 interrogated genes were tiled by 95.4±1.1 probes. Genes validated by tiling array or EST data are available online at http://www.hymenopteragenome.org/nasonia/?q=sequencing_and_analysis_consortium_datasets.

### Analysis of alternative splicing

We used two methods to obtain the alternative splicing status for *Nasonia* transcripts. First, we used the alternative splicing status from the OGS2 gene models with good intron information support. Genes with more than one OGS2 transcripts per gene were considered as alternatively spliced genes, and genes with a single form in OGS2 were considered as non-spliced genes. We also inferred the alternative splicing status from the adult female RNA-seq data using Cufflinks software. Moderately and highly expressed genes with expression level FPKM>2 were included in the study because sufficient RNA-seq coverage is needed to detect the alter-spliced forms in the RNA-seq data. Genes with the percentage of second most abundant forms greater than 10% were considered as alternatively spliced genes.

### Methylation conservation and GO-term enrichment analysis

For inference about conservation of methylation status of genes, loci were called *Nasonia*-specific if they did not have a homolog in OrthoDB BLASTp homolog (1e-5) to a database containing Human, Mouse, Xenopus, *Apis mellifera*, *Drosophila melanogaster*, and *Anopheles gambiae*. Arthropod-specific loci were those *Nasonia* sequences that had strong BLASTp hits (1e-5) to *Apis mellifera, Drosophila melanogaster, Anopheles gambiae*, but had no homology to proteins from Human, Mouse or Xenopus. GO term enrichment analysis was performed using blast2go [62] with the *Nasonia* OGS2 protein sequences and a BLASTp cut-off score of 1E-3 for assigning terms. Enrichment was determined using Fisher exact test as implemented by blast2go, with the cut-off for enrichment set to a 5% false discovery rate. The background gene set was restricted to the 17726 *Nasonia* genes with a known adult female methylation status as determined by bisulfite sequencing. For enrichment across different expression levels, genes were divided into low (9–11), medium (11–13) and high expression (13–15) based on median array expression (Table S11, S12, S13), with the background restricted to all genes with known methylation status that fell within that expression range. For GO-term analysis of genes with conserved methylation status between *Apis* and *Nasonia*, 1∶1 orthologs were selected based on their known methylation status for *Apis* (taken from [15]).

### Comparative genomic analysis of methylated genes

The orthology status for thirteen Hymenoptera insect species (*Acromyrmex echinatior, Apis florea, Apis mellifera, Atta cephalotes, Bombus impatiens, Bombus terrestris, Camponotus floridanus, Harpegnathos saltator, Linepithema humile, Megachile rotundata, Nasonia vitripennis, Pogonomyrmex barbatus, and Solenopsis invicta*) was obtained from OrthoDB [46]. The updated Official Gene Set 2.0 (OGS2) for *Nasonia vitripennis* was used in this analysis (http://arthropods.eugenes.org/genes2/nasonia/). The honeybee methylation status was from Zemach *et al.* 2010 [15]. The nucleotide substitution rates between three *Nasonia* species (*N. longicornis*, *N. giraulti* and *N. vitripennis*) were from the *Nasonia* genome project [34]. Analysis of paralogs that had undergone changes in methylation status was accomplished by first identifying all genes that had 1∶1 orthologs in thirteen sequenced hymenopteran genomes, but are duplicated in *N. vitripennis*, using the OrthoDB database [46]. These were then divided into categories based on methylation status. Rates of evolution of the *Nasonia* genes relative to the *Apis* orthologs were measured by comparing pairwise distances of protein alignments scores obtained from the AllAll tool (available at http://www.cbrg.ethz.ch/services/AllAll). Median expression level, range in expression and largest difference in expression were calculated using tiling microarray data.

### Statistical analyses

The logistic regression analysis of the effect of expression level and expression breadth on gene methylation status was performed using the LOGISTIC procedure in SAS 9.1 (Text S5). The statistical software R (version 2.13.0, www.r-project.org) was used for the rest of the statistical tests. Comparisons between matched gene samples were conducted using the Wilcoxon Matched-Pairs Signed Ranks Test (WMSRT) implemented in wilcox.test() function in the stats package. The test *P*-value of unimodality of gene expression distribution for methylated and non-methylated genes was calculated using the Hartigans' dip test for unimodality (dip package).

## Supporting Information

Dataset S1 Tiling array expression level for female developmental stages.
(XLSX)

Figure S1 Illumina WGBS-seq alignment strategies.
(TIF)

Figure S2 Illumina WGBS-seq coverage distribution and summary at CpG sites.
(TIF)

Figure S3 Distribution of methylation percentages for methylated CpG sites with methylation percentage >10%.
(TIF)

Figure S4 Validation of CpG methylation status for non-methylated gene Nasvi2EG001314 in adult females. (A) IGV browser screenshot of the WGBS-seq alignments in a 277 bp region on SCAFFOLD2, showing the CpG sites in non-methylated gene Nasvi2EG001314. All 65 covered CpGs in 5′ 1 kbp transcript region were non-methylated in the WGBS-seq data for this gene. (B) Zoom-in view for the boxed region in (A), demonstrating that all CpG were converted to TpGs in the WGBS-seq read alignments. (C) Plots of the gene model, translation start site and CpG methylation profile for Nasvi2EG001314. A vertical bar was drawn for each CpG at its position in the gene, color-coded by the methylation percentage in proportion to the bar length (blue: methylated Cs; red: non-methylated Cs). There are 143 covered CpGs in the gene region. (D) Bisulfite sequencing verification results for the 16 CpGs sites in the 201 bp amplicon at the 5′-coding region (shown in A) using the cloning method with 25 clones sequenced. The estimated methylation percentages at each CpG site from the WGBS-seq and single-gene bisulfite sequencing were shown on the top.
(TIF)

Figure S5 Validation of CpG methylation status for non-methylated gene Nasvi2EG000207 in adult females. (A) IGV browser screenshot of the WGBS-seq alignments in a 237 bp region on SCAFFOLD1 (1519410–1519646), showing the CpG sites in non-methylated gene Nasvi2EG000207. All 72 covered CpGs in 5′ 1 kbp transcript region were non-methylated in the WGBS-seq data for this gene. (B) Zoom-in view for the boxed region in (A), demonstrating that all CpG were converted to TpGs in the WGBS-seq read alignments. (C) Plots of the gene model, translation start site and CpG methylation profile for Nasvi2EG000207. A vertical bar was drawn for each CpG at its position in the gene, color-coded by the methylation percentage in proportion to the bar length (blue: methylated Cs; red: non-methylated Cs). There are 232 covered CpGs in the gene region. (D) Bisulfite sequencing verification results for the 17 CpGs sites in the 237 bp 5′-coding region (shown in A) using the cloning method with 22 clones sequenced. The estimated methylation percentages at each CpG site from the WGBS-seq and single-gene bisulfite sequencing were shown on the top. “?” stands for missing data at the end of the sequences.
(TIF)

Figure S6 Validation of CpG methylation status for non-methylated gene Nasvi2EG006064 in adult females. (A) IGV browser screenshot of the WGBS-seq alignments in a 403 bp region on SCAFFOLD9 (3192664–3193066), showing the CpG sites in non-methylated gene Nasvi2EG006064. All 75 covered CpGs in 5′ 1 kbp transcript region were non-methylated in the WGBS-seq data for this gene. (B) Zoom-in view for the boxed region in (A), demonstrating that all CpG were converted to TpGs in the WGBS-seq read alignments. (C) Plots of the gene model, translation start site and CpG methylation profile for Nasvi2EG006064. A vertical bar was drawn for each CpG at its position in the gene, color-coded by the methylation percentage in proportion to the bar length (blue: methylated Cs; red: non-methylated Cs). There are 137 covered CpGs in the gene region. (D) Bisulfite sequencing verification results for the 43 CpGs sites in the 403 bp 5′-coding region (shown in A) using the cloning method with 30 clones sequenced. The estimated methylation percentages at each CpG site from the WGBS-seq and single-gene bisulfite sequencing were shown on the top.
(TIF)

Figure S7 Validation of CpG methylation status for methylated gene Nasvi2EG002725 in adult females. (A) IGV browser screenshot of the WGBS-seq alignments in a 296 bp region on SCAFFOLD3 (3229802–3230097), showing the CpG sites in methylated gene Nasvi2EG002725. All 20 covered CpGs in 5′ 1 kbp transcript region were methylated in the WGBS-seq data for this gene. (B) Zoom-in view for the boxed region in (A), demonstrating that the C in CpG context remains a C after bisulfite conversion. (C) Plots of the gene model, translation start site and CpG methylation profile for Nasvi2EG002725. A vertical bar was drawn for each CpG at its position in the gene, color-coded by the methylation percentage in proportion to the bar length (blue: methylated Cs; red: non-methylated Cs). There are 125 covered CpGs in the gene region. (D) Bisulfite sequencing verification results for the 9 CpGs sites in the 296 bp 5′-coding region (shown in A) using the cloning method with 25 clones sequenced. The estimated methylation percentages at each CpG site from the WGBS-seq and single-gene bisulfite sequencing were shown on the top. Percentages of mCpG labeled in gray in the WGBS-seq data are the ones with less than 10 read coverage.
(TIF)

Figure S8 Validation of CpG methylation status for methylated gene Nasvi2EG000295 in adult females. (A) Plots of the gene model, translation start site and CpG methylation profile for Nasvi2EG000295. A vertical bar was drawn for each CpG at its position in the gene, color-coded by the methylation percentage in proportion to the bar length (blue: methylated Cs; red: non-methylated Cs). There are 102 covered CpGs in the gene region. (B) Bisulfite sequencing verification results for the 7 CpGs sites in the 357 bp 5′-coding region using the cloning method with 20 clones sequenced. The estimated methylation percentages at each CpG site from the WGBS-seq and single-gene bisulfite sequencing were shown on the top. Percentages of mCpG labeled in gray in the WGBS-seq data are the ones with less than 10 read coverage.
(TIF)

Figure S9 Validation of CpG methylation status for methylated gene Nasvi2EG003593 in adult females. (A) IGV browser screenshot of the WGBS-seq alignments in a 283 bp region on SCAFFOLD4 (5219843–5220125), showing the CpG sites in methylated gene Nasvi2EG003593. All 19 covered CpGs in 5′ 1 kbp transcript region were methylated in the WGBS-seq data for this gene. (B) Zoom-in view for the boxed region in (A), demonstrating that the C in CpG context remains a C after bisulfite conversion. (C) Plots of the gene model, translation start site and CpG methylation profile for Nasvi2EG003593. A vertical bar was drawn for each CpG at its position in the gene, color-coded by the methylation percentage in proportion to the bar length (blue: methylated Cs; red: non-methylated Cs). There are 48 covered CpGs in the gene region. (D) Bisulfite sequencing verification results for the 8 CpGs sites in the 283 bp 5′-coding region (shown in A) using the cloning method with 14 clones sequenced. The estimated methylation percentages at each CpG site from the WGBS-seq and single-gene bisulfite sequencing were shown on the top. Percentages of mCpG labeled in gray in the WGBS-seq data are the ones with less than 10 read coverage.
(TIF)

Figure S10 DNA methylation and observed/expected CpG ratios (CpG O/E). (A) Histograms for distribution of CpG O/E ratios in the 5′ 1 kbp coding region for methylated (blue), non-methylated (red) and all genes (purple). (B) Distribution of CpG O/E ratios in classes of genes with different percentage of methylated CpG sites in 5′ 1 kbp coding region. Red: non-methylated genes; blue: methylated genes. (C) Top: Stacked barplot GC content in methylated (blue) and non-methylated genes (red). Middle: scatterplot of GC percent and CpG O/E ratios in methylated genes. Bottom: scatterplot of GC percent and CpG O/E ratios in non-methylated genes.
(TIF)

Figure S11 Clustering of methylated genes in *Nasonia* genome. (A) Fourfold plot of the neighboring methylated-methylated genes (MM), non-methylated-non-methylated genes (NN) and methylated-non-methylated genes (MN) and non-methylated-methylated (NM). (B) Middle panel: Counts of non-overlapping close neighboring genes (<1 kb distance) in four orientation categories (Head-Head, Tail-Tail, Head-Tail and Tail-Head). Top panel: Percentage of methylated genes for the first gene (orange) and second gene (green) gene in the four categories (HH, TT, HT and TH). The red horizontal line is the genome average. Bottom panel: barplot of methylation status for HH, TT and HT/TH groups. The expected percentages for each category were plotted as a block dot.
(TIF)

Figure S12 Distribution of percentages of unconverted Cs at non-CpG sites.
(TIF)

Figure S13 Eight candidate non-CpG methylation sites in which the methylation is actually in CpG context due to reference sequence error or paralogous sequences in the genome. The IGV browser screenshot was shown for each candidate non-CpG methylation sites. The unconverted Cs were in CpG context instead of non-CpG context. (A) A spurious non-CpG methylation site due to reference genome sequencing error. (B–H) seven examples of spurious non-CpG methylation sites due to paralogous sequences in the genome.
(TIF)

Figure S14 Validation of non-CpG methylation site in gene Nasvi2EG004247 in adult females. (A) IGV browser screenshot of the WGBS-seq alignments (top) and RNA-seq coverage (bottom) for Nasvi2EG004247 gene region on SCAFFOLD6 (1765528–1768213), showing the CpG sites methylation in this gene. The candidate non-CpG methylation site at position 1767201 is labeled in the red box. (B) Zoom-in view for the boxed region in (A), demonstrating that the non-CpG methylation in CAT context on the minus strand, with 42% methylated Cs estimated from the WGBS-seq reads. (C) Plots of the gene model, translation start site and CpG methylation profile for Nasvi2EG004247. A vertical bar was drawn for each CpG at its position in the gene, color-coded by the methylation percentage in proportion to the bar length (blue: methylated Cs in CpGs; red: unmethylated Cs in CpGs). There are 44 covered CpGs in the gene region. The 252 bp target region for bisulfite sequencing validation of the non-CpG methylation is labeled at the bottom. (D) Bisulfite sequencing verification results at the candidate non-CpG methylation site (site #14). The estimated methylation percentages at all C positions from the WGBS-seq and single-gene bisulfite sequencing were shown on the top. There are one CpG C (site #20) and 27 non-CpG Cs in this region. 10/19 (53%) clones have a C at the CpG C position, which is consistent with the methylation status in WGBS-seq data. Among the rest of the 27 non-CpG Cs, only the candidate non-CpG site has unconverted C in more than one clone. The non-CpG methylation at site #14 was confirmed and 3/19 (16%) clones have unconverted Cs.
(TIF)

Figure S15 Distribution of the normalized tiling array expression values in five developmental stages. Plotted on the *x*-axis is the normalized tiling array expression value (log_2_). The *y*-axis is the gene count for each stage. The median expression value for each stage is labeled with the red vertical line.
(TIF)

Figure S16 Stacked barplot for expressed methylated and non-methylated genes. Stacked barplot of methylated and non-methylated genes with adult RNA-seq expression level FPKM ≥1, binned by different expression level categories. Red: non-methylated genes; blue: methylated genes.
(TIF)

Figure S17 DNA methylation status and tiling array median expression level. Distribution of median tiling array expression level (log_2_) for methylated (blue), non-methylated (red) and all genes (purple).
(TIF)

Figure S18 Expression breadth and the adult female RNA-seq expression level for methylation and non-methylated genes. Plotted on the *y*-axis is the log_10_ coefficient of variation (CV) for tiling array expression values in five developmental stages. On the *x*-axis is the RNA-seq expression level in adult female samples (log_10_ FPKM). The methylated genes were represented with blue dot and non-methylated genes with red dot. The fitted curve and confidence interval using non-parametric local regression for methylated and non-methylated genes were plotted in blue and red curve, respectively.
(TIF)

Figure S19 Expression breadth and the adult female tiling array expression level for methylated and non-methylated genes. Scatterplot of expression breadth (log2 expression CV) on *y*-axis against adult female gene expression level (log2 signal intensity) in tiling array on *x*-axis, color-coded by adult female methylation status (blue: methylated genes; red: non-methylated genes). Fitted lines using non-parametric local regression are shown for methylated and non-methylated genes respectively.
(TIF)

Figure S20 DNA methylation status and gene expression level, expression breadth and number of expressed tissues. Relationship between DNA methylation status, gene expression level, expression CV and number of expressed stages. Plotted on the *y*-axis is the average expression CV, and on the *x*-axis is the average gene expression level. Methylated (in blue) and non-methylated genes (in red) present in 0–5 developmental stages are plotted as separate round dot. The size of the area is in proportion to the number of genes in each category.
(TIF)

Figure S21 Enriched Gene Ontology categories for methylated gene in *Nasonia* genome.
(TIF)

Figure S22 Distribution of RNA-seq expression level for the four methylation-alternative splicing classes. Plotted here is the distribution of adult female RNA-seq expression level (log_10_ FPKM) for alternatively spliced methylated, alternatively spliced non-methylated, non-alternatively spliced methylated, non-alternatively spliced non-methylated genes (from left to right). For methylated genes, the expression levels of alternatively spliced genes were not significantly higher than the non-alternatively spliced ones (*P*-value = 0.67, Kolmogorov-Smirnov test, one side). For non-methylated genes, the expression levels of alternatively spliced genes were significantly higher than the non-alternatively spliced ones (*P*-value<2.2×10^−16^, Kolmogorov-Smirnov test, one side).
(TIF)

Figure S23 Correlation between percentage of mCpGs and fraction of major spliced form in alternatively spliced methylated genes. Scatterplot for percentage of methylated CpGs and fraction of major spliced form in alternatively spliced methylated genes. The fitted lines using non-parametric local regression are shown in red.
(TIF)

Figure S24 Gene expression, DNA methylation and alternative splicing profile for three methylated genes. (A) Nasvi2EG000107 showing differential 5′-exon usage. (B) Nasvi2EG013697 showing differential middle exon usage. (C) Nasvi2EG022273 showing intron retention. For each panel, plotted at the top is the IGV browser screenshot showing adult female RNA-seq coverage (on log scale) and read alignments in the gene region. Plotted at the bottom are the CpG methylation profile at covered CpG sites from WGBS-seq data and the exon model of the alternatively spliced transcripts from OGS2 gene models. The locations of methylated CpG clusters were shown as blue horizontal boxes in (A). A vertical bar was drawn for each CpG at its position in the gene, color-coded by the methylation percentage in proportion to the bar length (blue: methylated Cs; red: non-methylated Cs). OGS2 transcript variants detected in the RNA-seq data with high abundance were plotted at the bottom. The remaining minor forms were not shown in this figure.
(TIF)

Table S1 Summary of Illumina sequencing error rates estimated from lambda control DNA alignments.
(DOC)

Table S2 Summary for methylated and non-methylated CpGs in *Nasonia* genome.
(DOC)

Table S3 Methylated CpG clusters in genic region in *Nasonia* OGS1.2 but absent in OGS2.
(DOC)

Table S4 Top five methylated repetitive TE families and the adult female RNA-seq coverage.
(DOC)

Table S5 Summary for unconverted Cs in non-CpG context in WGBS-seq data.
(DOC)

Table S6 Candidate non-CpG methylation sites with >30% unconverted Cs in non-CpG context.
(DOC)

Table S7 Logistic regression analysis results for methylation status with gene expression and expression breadth as predictors.
(DOC)

Table S8 Logistic regression analysis for methylation status with gene expression or expression breadth as single predictor.
(DOC)

Table S9 Mean/median expression CV for methylated and non-methylated genes in median array expression level categories.
(DOC)

Table S10 Twenty most significant GO terms enriched amongst all methylated *Nasonia* genes.
(DOC)

Table S11 Enriched GO terms amongst methylated genes with median array expression levels 9–11 (low expression).
(DOC)

Table S12 Enriched GO terms amongst methylated genes with median array expression levels 11–13 (medium expression).
(DOC)

Table S13 Enriched GO terms amongst methylated genes with median array expression levels 13–15 (high expression).
(DOC)

Table S14 Methylated genes containing two or more methylated CpG clusters (mCGCLs) with differential 5′exon usage.
(DOC)

Table S15 Statistical significance of expression breadth difference between methylated and non-methylated genes in three conservation categories.
(DOC)

Table S16 Ten most significant enriched GO terms for conserved genes methylated in both *Nasonia* and *Apis*.
(DOC)

Table S17 Enriched GO terms amongst *Nasonia* methylated genes that are conserved but not methylated in *Apis*.
(DOC)

Table S18 Duplicated genes in *Nasonia* and their methylation status.
(DOC)

Text S1 Validation of methylation status estimated from WGBS-seq with single-gene bisulfite sequencing using cloning method.
(DOC)

Text S2 mCpGs are organized in methylated CpG clusters while non-coding and CpG islands are non-methylated.
(DOC)

Text S3 DNA methylation and observed/expected CpG ratios.
(DOC)

Text S4 Quantification and validation of non-CpG methylation in the *Nasonia* genome.
(DOC)

Text S5 Logistic regression analysis of the effect of expression and expression breadth on gene methylation status.
(DOC)

Text S6 Potential links between DNA methylation and alternative splicing.
(DOC)

Text S7 Calculation of global methylation (mC/C) in *Nasonia*, *Apis* and ants.
(DOC)
