# Supplementary figures and images for: Function and Evolution of DNA Methylation in Nasonia vitripennis
Source: PLoS Genet. 2013 Oct 10;9(10):e1003872. doi: 10.1371/journal.pgen.1003872 (PMC3794928; doi:10.1371/journal.pgen.1003872)

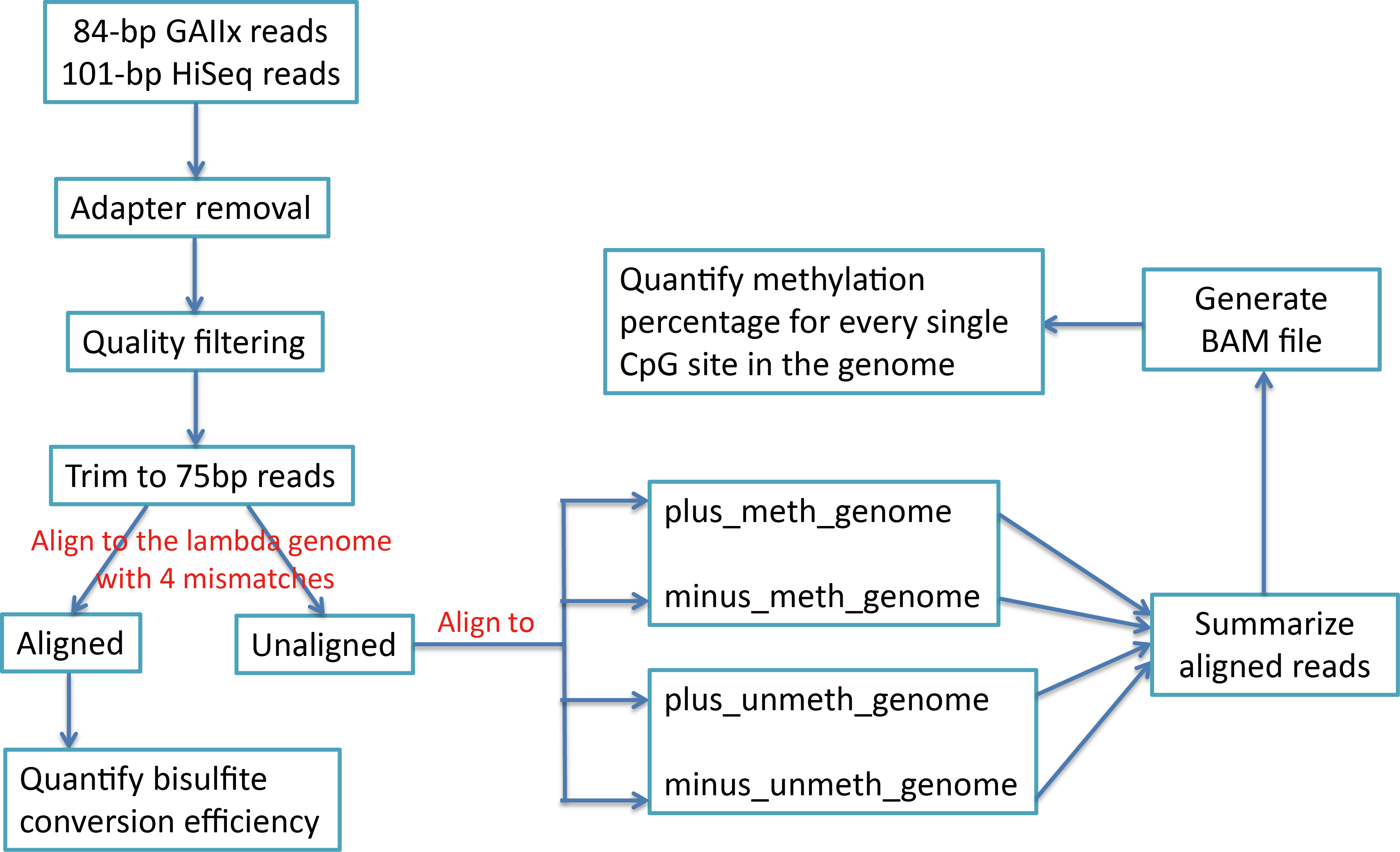

Supplement: Figure S1 — Illumina WGBS-seq alignment strategies. (TIF) [file pgen.1003872.s002.tif]

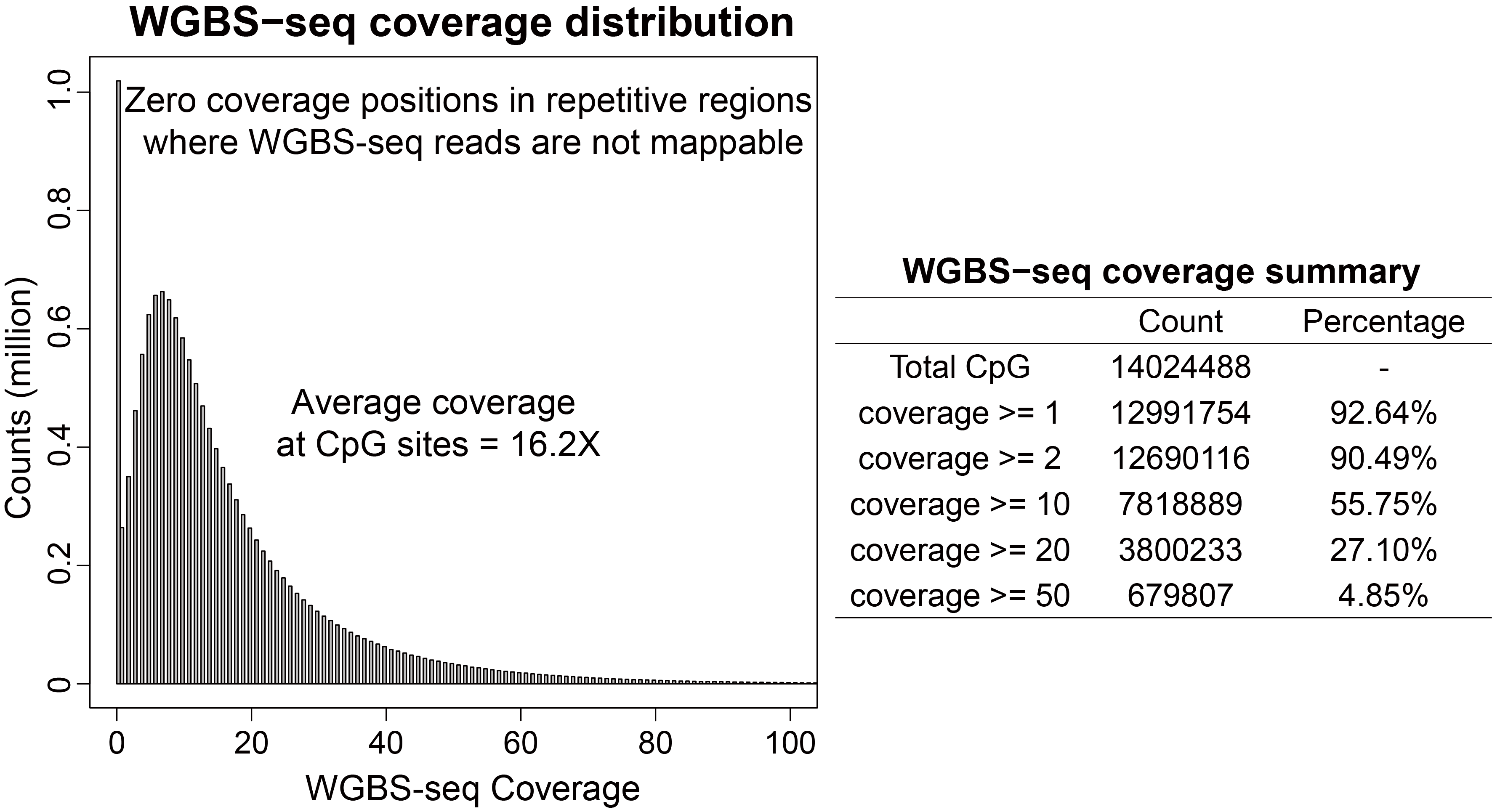

Supplement: Figure S2 — Illumina WGBS-seq coverage distribution and summary at CpG sites. (TIF) [file pgen.1003872.s003.tif]

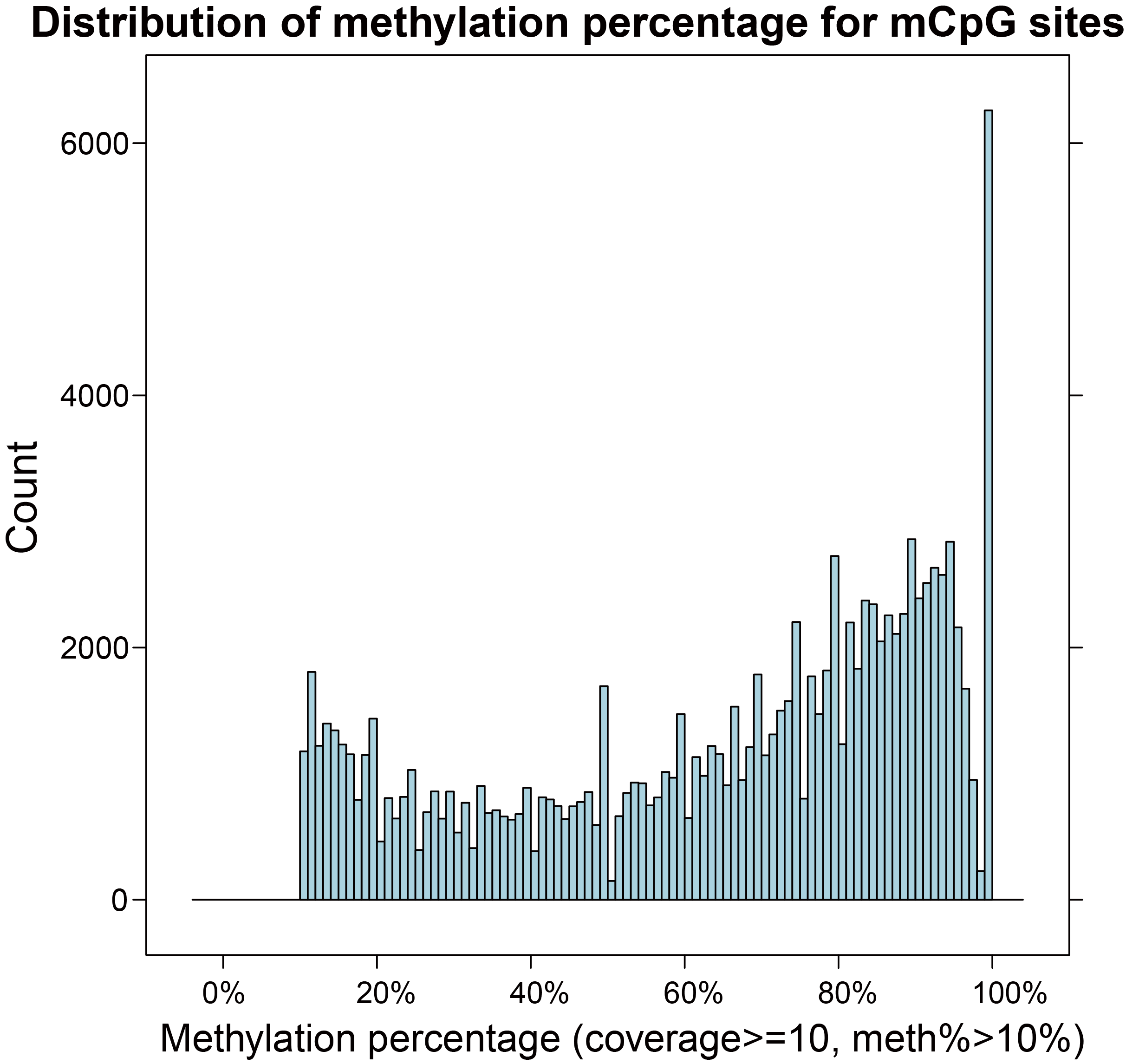

Supplement: Figure S3 — Distribution of methylation percentages for methylated CpG sites with methylation percentage >10%. (TIF) [file pgen.1003872.s004.tif]

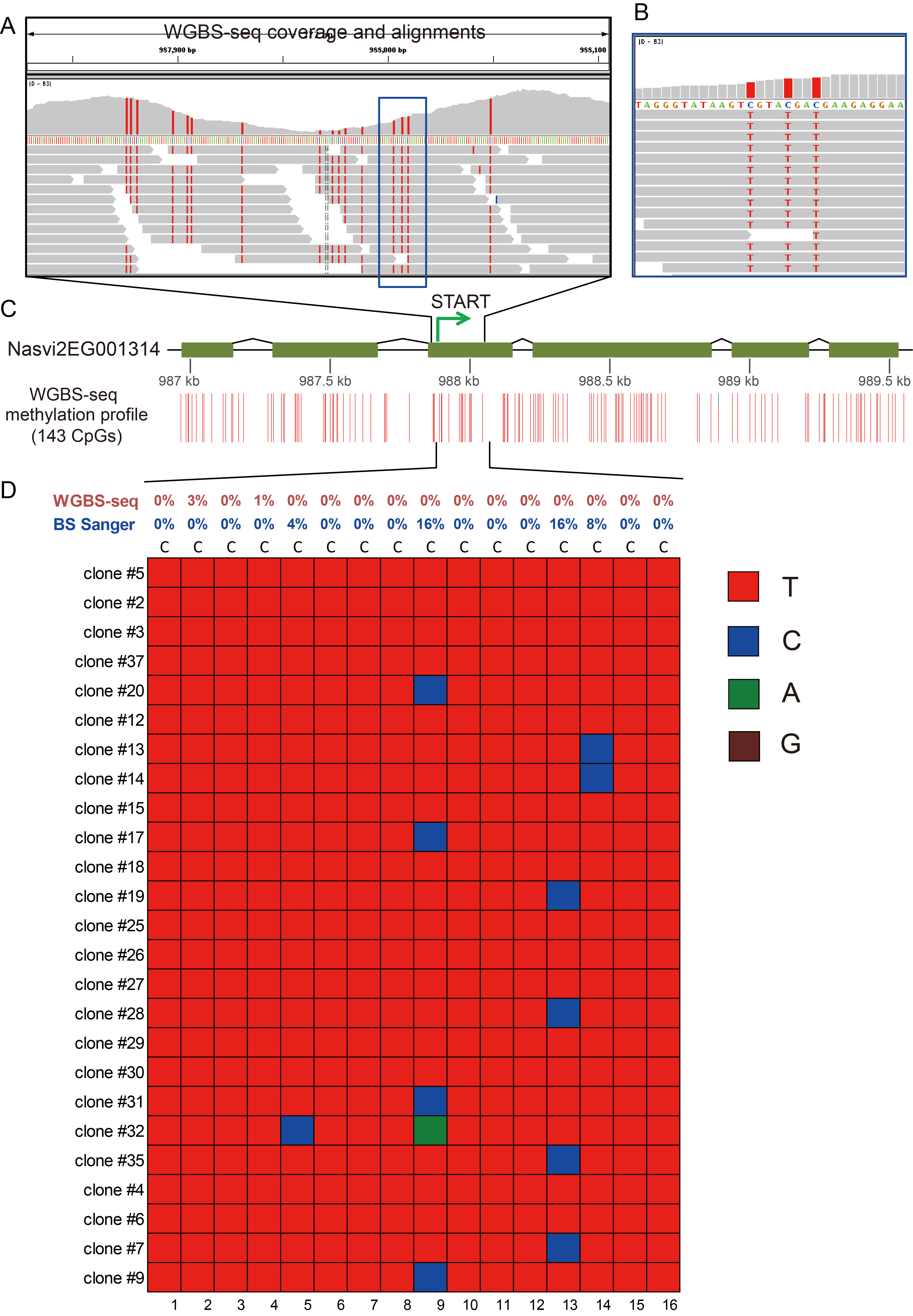

Supplement: Figure S4 — Validation of CpG methylation status for non-methylated gene Nasvi2EG001314 in adult females. (A) IGV browser screenshot of the WGBS-seq alignments in a 277 bp region on SCAFFOLD2, showing the CpG sites in non-methylated gene Nasvi2EG001314. All 65 covered CpGs in 5′ 1 kbp transcript region were non-methylated in the WGBS-seq data for this gene. (B) Zoom-in view for the boxed region in (A), demonstrating that all CpG were converted to TpGs in the WGBS-seq read alignments. (C) Plots of the gene model, translation start site and CpG methylation profile for Nasvi2EG001314. A vertical bar was drawn for each CpG at its position in the gene, color-coded by the methylation percentage in proportion to the bar length (blue: methylated Cs; red: non-methylated Cs). There are 143 covered CpGs in the gene region. (D) Bisulfite sequencing verification results for the 16 CpGs sites in the 201 bp amplicon at the 5′-coding region (shown in A) using the cloning method with 25 clones sequenced. The estimated methylation percentages at each CpG site from the WGBS-seq and single-gene bisulfite sequencing were shown on the top. (TIF) [file pgen.1003872.s005.tif]

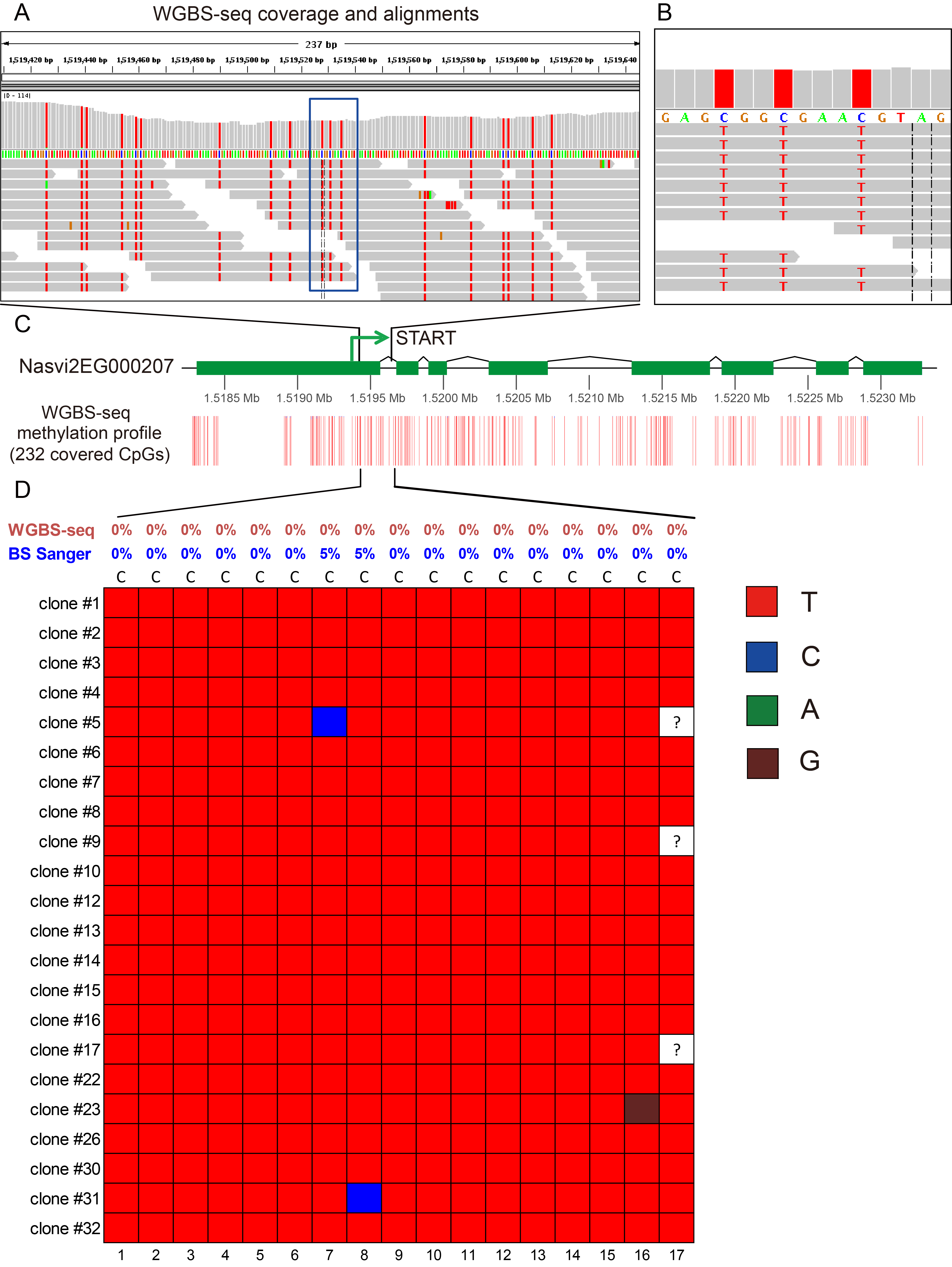

Supplement: Figure S5 — Validation of CpG methylation status for non-methylated gene Nasvi2EG000207 in adult females. (A) IGV browser screenshot of the WGBS-seq alignments in a 237 bp region on SCAFFOLD1 (1519410–1519646), showing the CpG sites in non-methylated gene Nasvi2EG000207. All 72 covered CpGs in 5′ 1 kbp transcript region were non-methylated in the WGBS-seq data for this gene. (B) Zoom-in view for the boxed region in (A), demonstrating that all CpG were converted to TpGs in the WGBS-seq read alignments. (C) Plots of the gene model, translation start site and CpG methylation profile for Nasvi2EG000207. A vertical bar was drawn for each CpG at its position in the gene, color-coded by the methylation percentage in proportion to the bar length (blue: methylated Cs; red: non-methylated Cs). There are 232 covered CpGs in the gene region. (D) Bisulfite sequencing verification results for the 17 CpGs sites in the 237 bp 5′-coding region (shown in A) using the cloning method with 22 clones sequenced. The estimated methylation percentages at each CpG site from the WGBS-seq and single-gene bisulfite sequencing were shown on the top. “?” stands for missing data at the end of the sequences. (TIF) [file pgen.1003872.s006.tif]

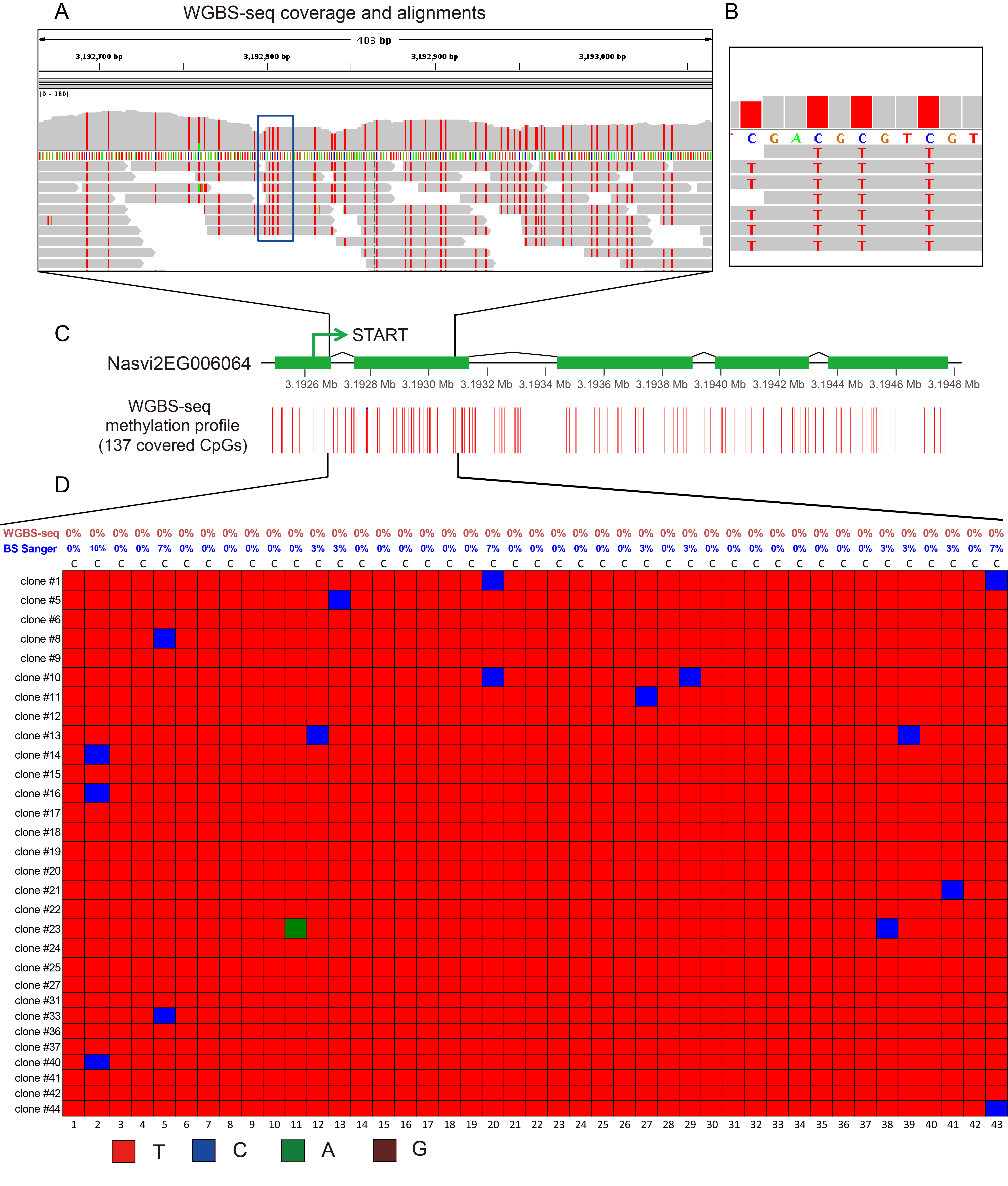

Supplement: Figure S6 — Validation of CpG methylation status for non-methylated gene Nasvi2EG006064 in adult females. (A) IGV browser screenshot of the WGBS-seq alignments in a 403 bp region on SCAFFOLD9 (3192664–3193066), showing the CpG sites in non-methylated gene Nasvi2EG006064. All 75 covered CpGs in 5′ 1 kbp transcript region were non-methylated in the WGBS-seq data for this gene. (B) Zoom-in view for the boxed region in (A), demonstrating that all CpG were converted to TpGs in the WGBS-seq read alignments. (C) Plots of the gene model, translation start site and CpG methylation profile for Nasvi2EG006064. A vertical bar was drawn for each CpG at its position in the gene, color-coded by the methylation percentage in proportion to the bar length (blue: methylated Cs; red: non-methylated Cs). There are 137 covered CpGs in the gene region. (D) Bisulfite sequencing verification results for the 43 CpGs sites in the 403 bp 5′-coding region (shown in A) using the cloning method with 30 clones sequenced. The estimated methylation percentages at each CpG site from the WGBS-seq and single-gene bisulfite sequencing were shown on the top. (TIF) [file pgen.1003872.s007.tif]

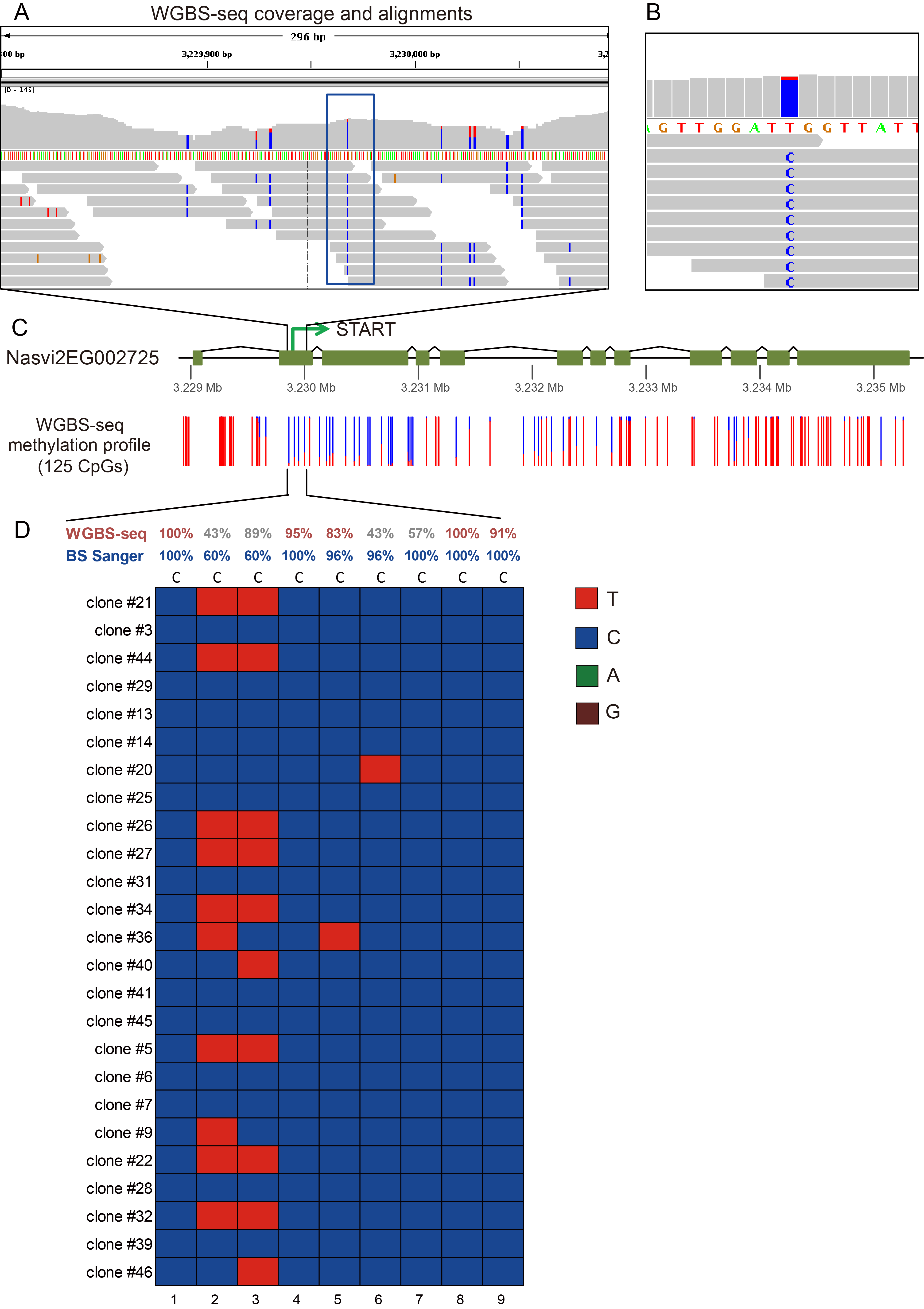

Supplement: Figure S7 — Validation of CpG methylation status for methylated gene Nasvi2EG002725 in adult females. (A) IGV browser screenshot of the WGBS-seq alignments in a 296 bp region on SCAFFOLD3 (3229802–3230097), showing the CpG sites in methylated gene Nasvi2EG002725. All 20 covered CpGs in 5′ 1 kbp transcript region were methylated in the WGBS-seq data for this gene. (B) Zoom-in view for the boxed region in (A), demonstrating that the C in CpG context remains a C after bisulfite conversion. (C) Plots of the gene model, translation start site and CpG methylation profile for Nasvi2EG002725. A vertical bar was drawn for each CpG at its position in the gene, color-coded by the methylation percentage in proportion to the bar length (blue: methylated Cs; red: non-methylated Cs). There are 125 covered CpGs in the gene region. (D) Bisulfite sequencing verification results for the 9 CpGs sites in the 296 bp 5′-coding region (shown in A) using the cloning method with 25 clones sequenced. The estimated methylation percentages at each CpG site from the WGBS-seq and single-gene bisulfite sequencing were shown on the top. Percentages of mCpG labeled in gray in the WGBS-seq data are the ones with less than 10 read coverage. (TIF) [file pgen.1003872.s008.tif]

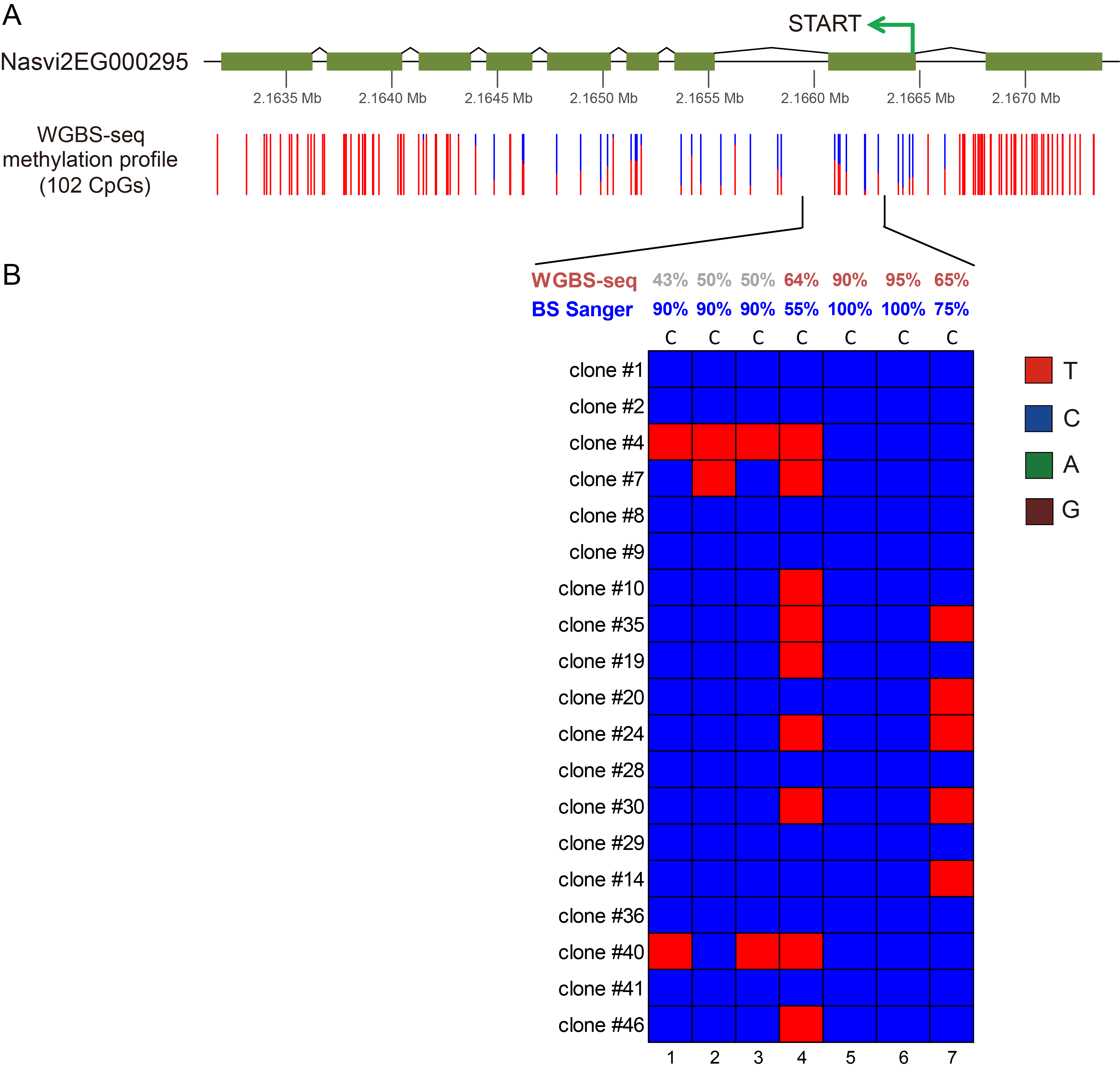

Supplement: Figure S8 — Validation of CpG methylation status for methylated gene Nasvi2EG000295 in adult females. (A) Plots of the gene model, translation start site and CpG methylation profile for Nasvi2EG000295. A vertical bar was drawn for each CpG at its position in the gene, color-coded by the methylation percentage in proportion to the bar length (blue: methylated Cs; red: non-methylated Cs). There are 102 covered CpGs in the gene region. (B) Bisulfite sequencing verification results for the 7 CpGs sites in the 357 bp 5′-coding region using the cloning method with 20 clones sequenced. The estimated methylation percentages at each CpG site from the WGBS-seq and single-gene bisulfite sequencing were shown on the top. Percentages of mCpG labeled in gray in the WGBS-seq data are the ones with less than 10 read coverage. (TIF) [file pgen.1003872.s009.tif]

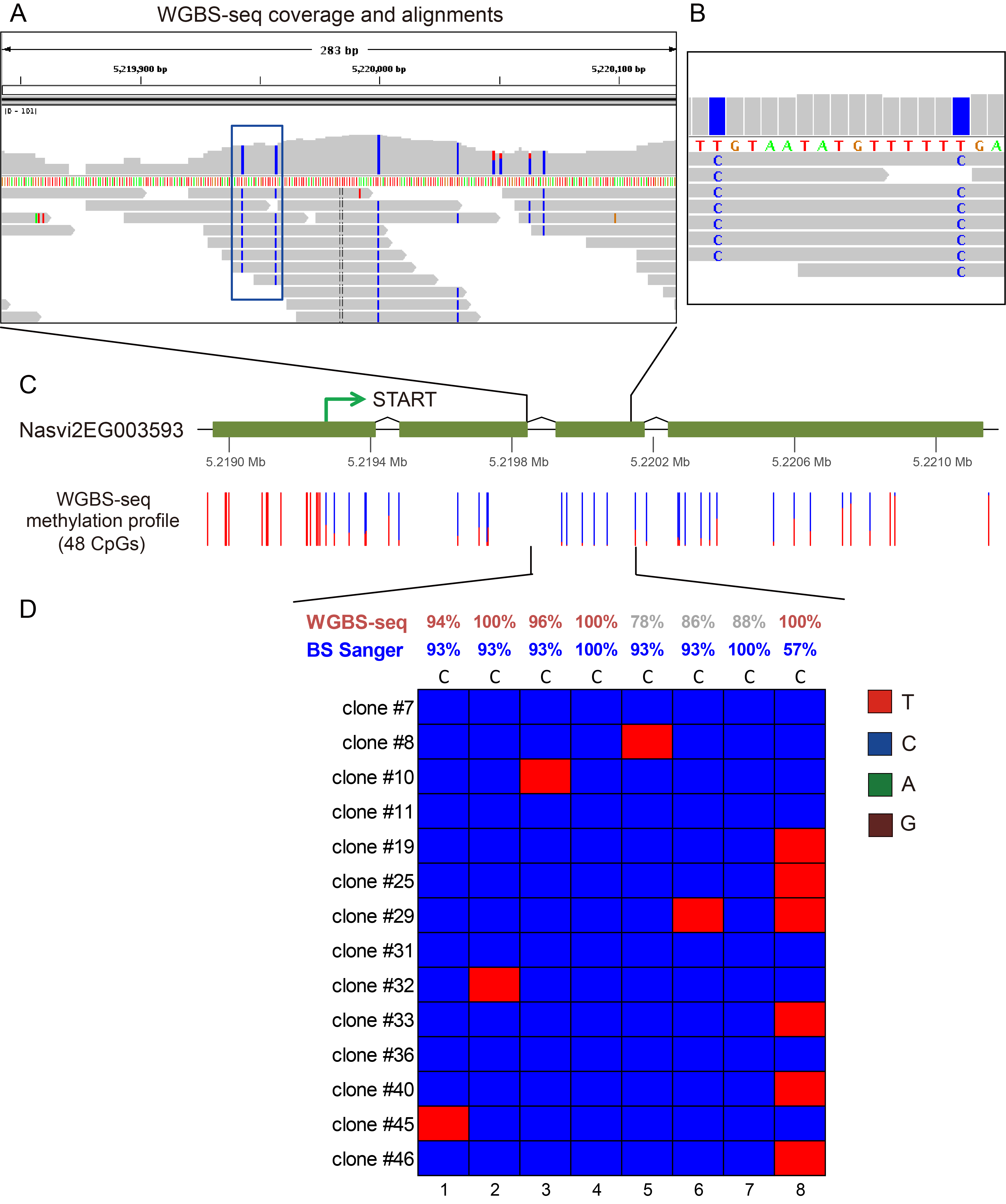

Supplement: Figure S9 — Validation of CpG methylation status for methylated gene Nasvi2EG003593 in adult females. (A) IGV browser screenshot of the WGBS-seq alignments in a 283 bp region on SCAFFOLD4 (5219843–5220125), showing the CpG sites in methylated gene Nasvi2EG003593. All 19 covered CpGs in 5′ 1 kbp transcript region were methylated in the WGBS-seq data for this gene. (B) Zoom-in view for the boxed region in (A), demonstrating that the C in CpG context remains a C after bisulfite conversion. (C) Plots of the gene model, translation start site and CpG methylation profile for Nasvi2EG003593. A vertical bar was drawn for each CpG at its position in the gene, color-coded by the methylation percentage in proportion to the bar length (blue: methylated Cs; red: non-methylated Cs). There are 48 covered CpGs in the gene region. (D) Bisulfite sequencing verification results for the 8 CpGs sites in the 283 bp 5′-coding region (shown in A) using the cloning method with 14 clones sequenced. The estimated methylation percentages at each CpG site from the WGBS-seq and single-gene bisulfite sequencing were shown on the top. Percentages of mCpG labeled in gray in the WGBS-seq data are the ones with less than 10 read coverage. (TIF) [file pgen.1003872.s010.tif]

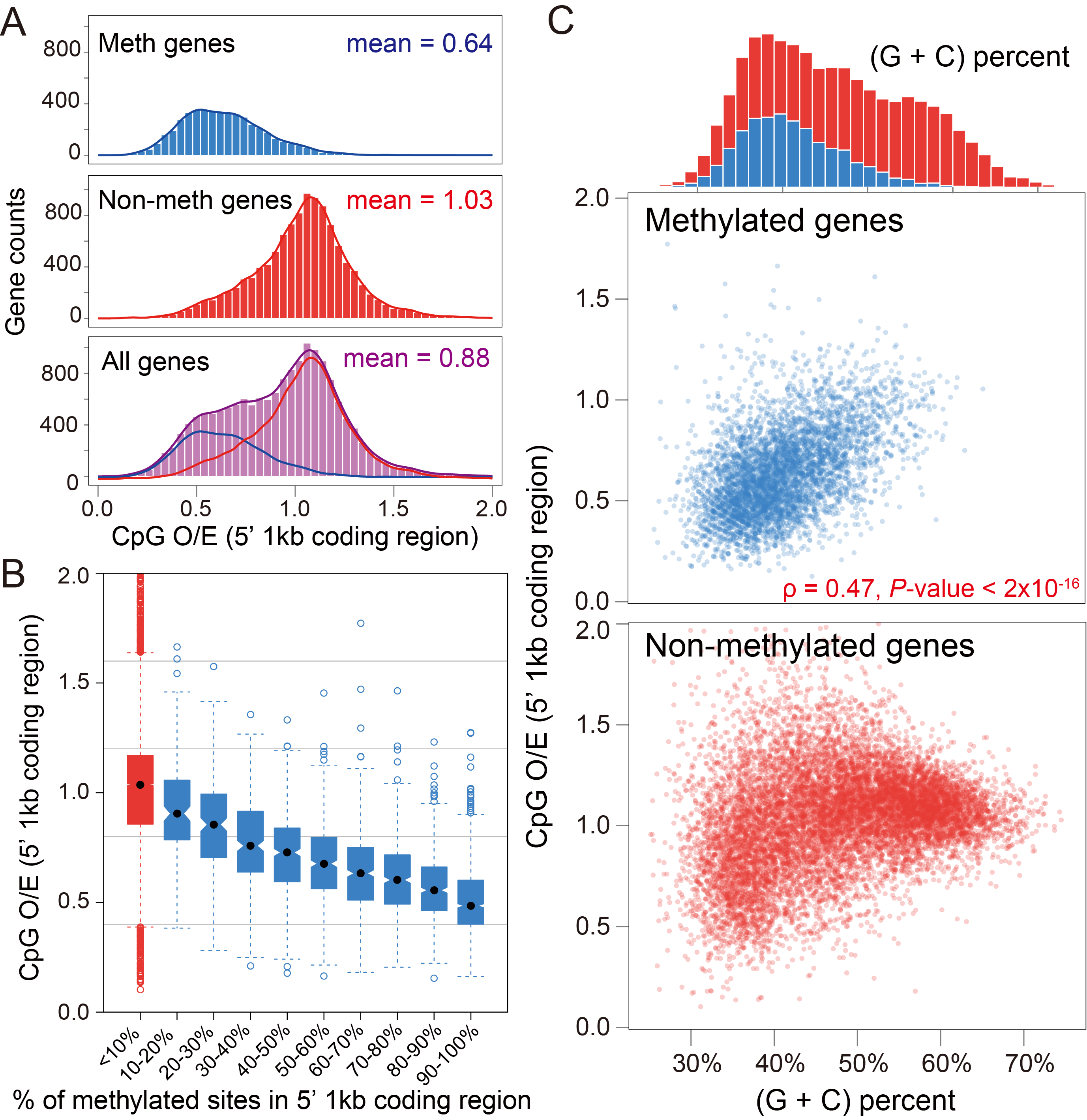

Supplement: Figure S10 — DNA methylation and observed/expected CpG ratios (CpG O/E). (A) Histograms for distribution of CpG O/E ratios in the 5′ 1 kbp coding region for methylated (blue), non-methylated (red) and all genes (purple). (B) Distribution of CpG O/E ratios in classes of genes with different percentage of methylated CpG sites in 5′ 1 kbp coding region. Red: non-methylated genes; blue: methylated genes. (C) Top: Stacked barplot GC content in methylated (blue) and non-methylated genes (red). Middle: scatterplot of GC percent and CpG O/E ratios in methylated genes. Bottom: scatterplot of GC percent and CpG O/E ratios in non-methylated genes. (TIF) [file pgen.1003872.s011.tif]

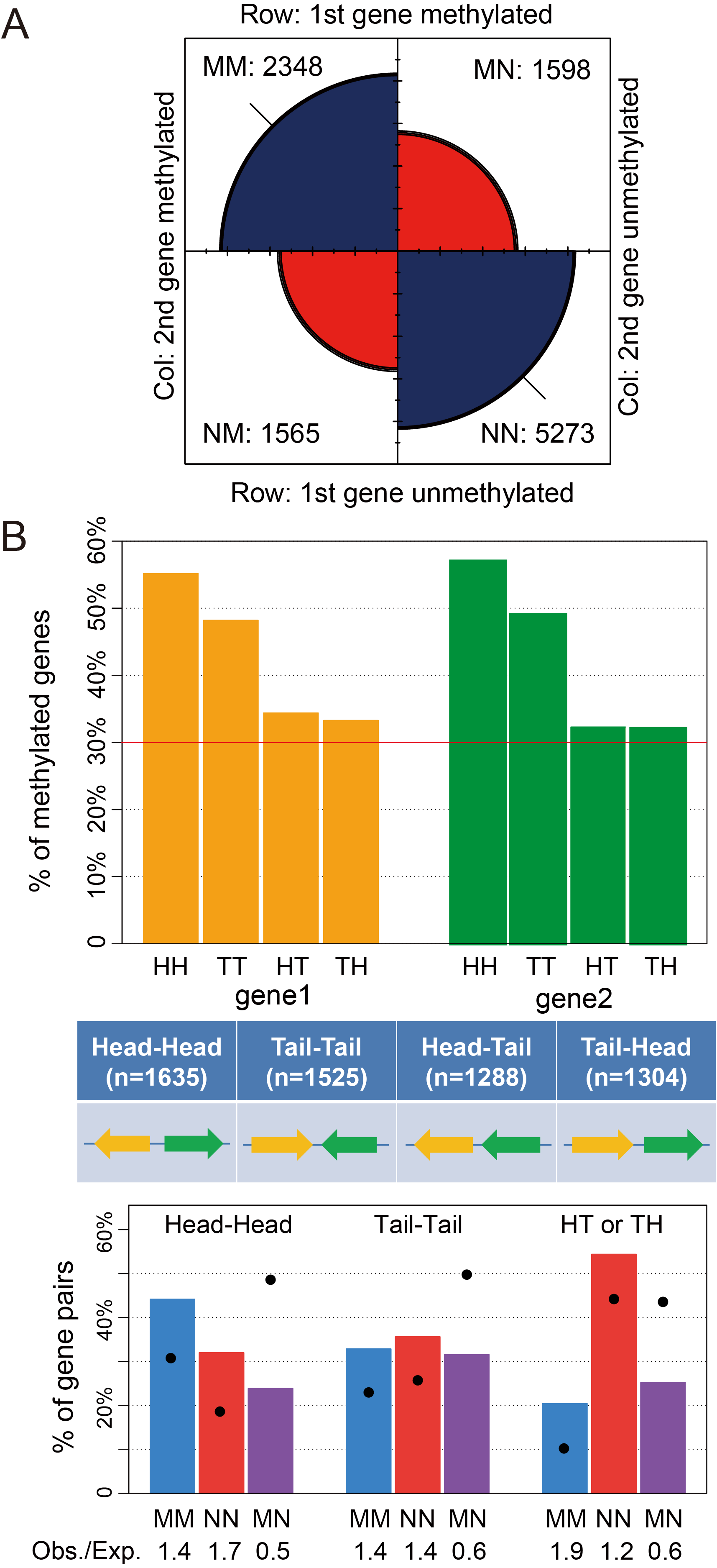

Supplement: Figure S11 — Clustering of methylated genes in Nasonia genome. (A) Fourfold plot of the neighboring methylated-methylated genes (MM), non-methylated-non-methylated genes (NN) and methylated-non-methylated genes (MN) and non-methylated-methylated (NM). (B) Middle panel: Counts of non-overlapping close neighboring genes (<1 kb distance) in four orientation categories (Head-Head, Tail-Tail, Head-Tail and Tail-Head). Top panel: Percentage of methylated genes for the first gene (orange) and second gene (green) gene in the four categories (HH, TT, HT and TH). The red horizontal line is the genome average. Bottom panel: barplot of methylation status for HH, TT and HT/TH groups. The expected percentages for each category were plotted as a block dot. (TIF) [file pgen.1003872.s012.tif]

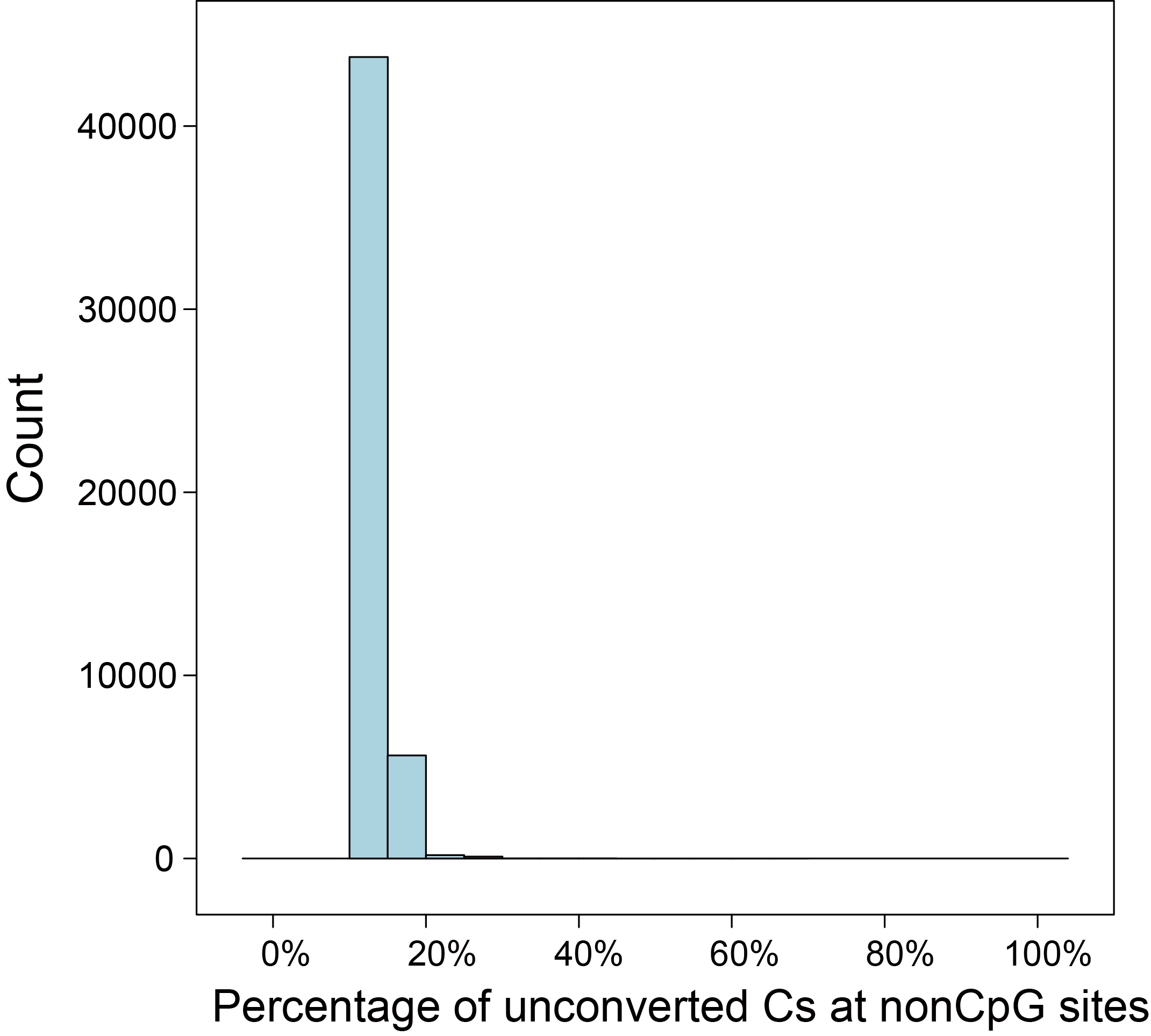

Supplement: Figure S12 — Distribution of percentages of unconverted Cs at non-CpG sites. (TIF) [file pgen.1003872.s013.tif]

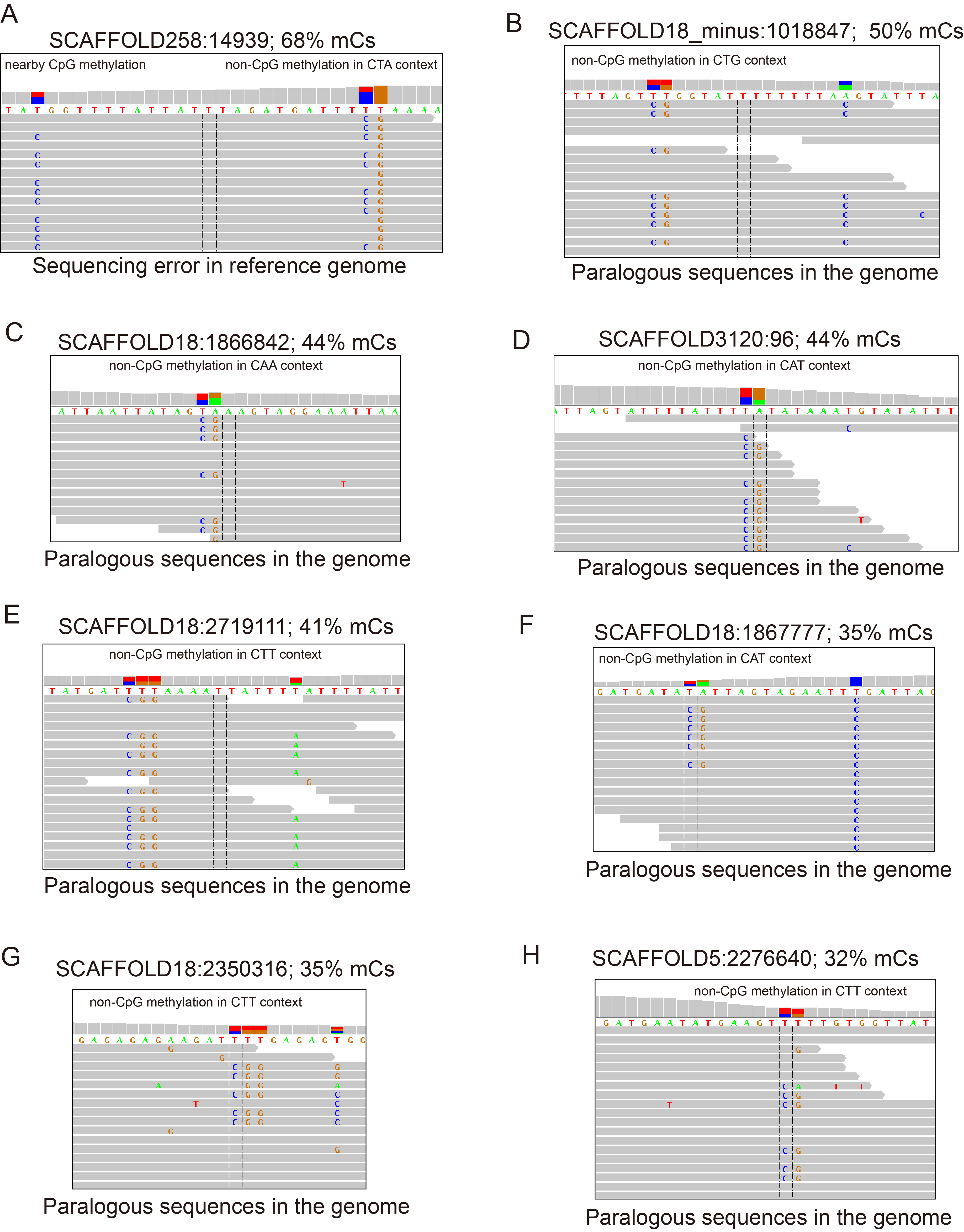

Supplement: Figure S13 — Eight candidate non-CpG methylation sites in which the methylation is actually in CpG context due to reference sequence error or paralogous sequences in the genome. The IGV browser screenshot was shown for each candidate non-CpG methylation sites. The unconverted Cs were in CpG context instead of non-CpG context. (A) A spurious non-CpG methylation site due to reference genome sequencing error. (B–H) seven examples of spurious non-CpG methylation sites due to paralogous sequences in the genome. (TIF) [file pgen.1003872.s014.tif]

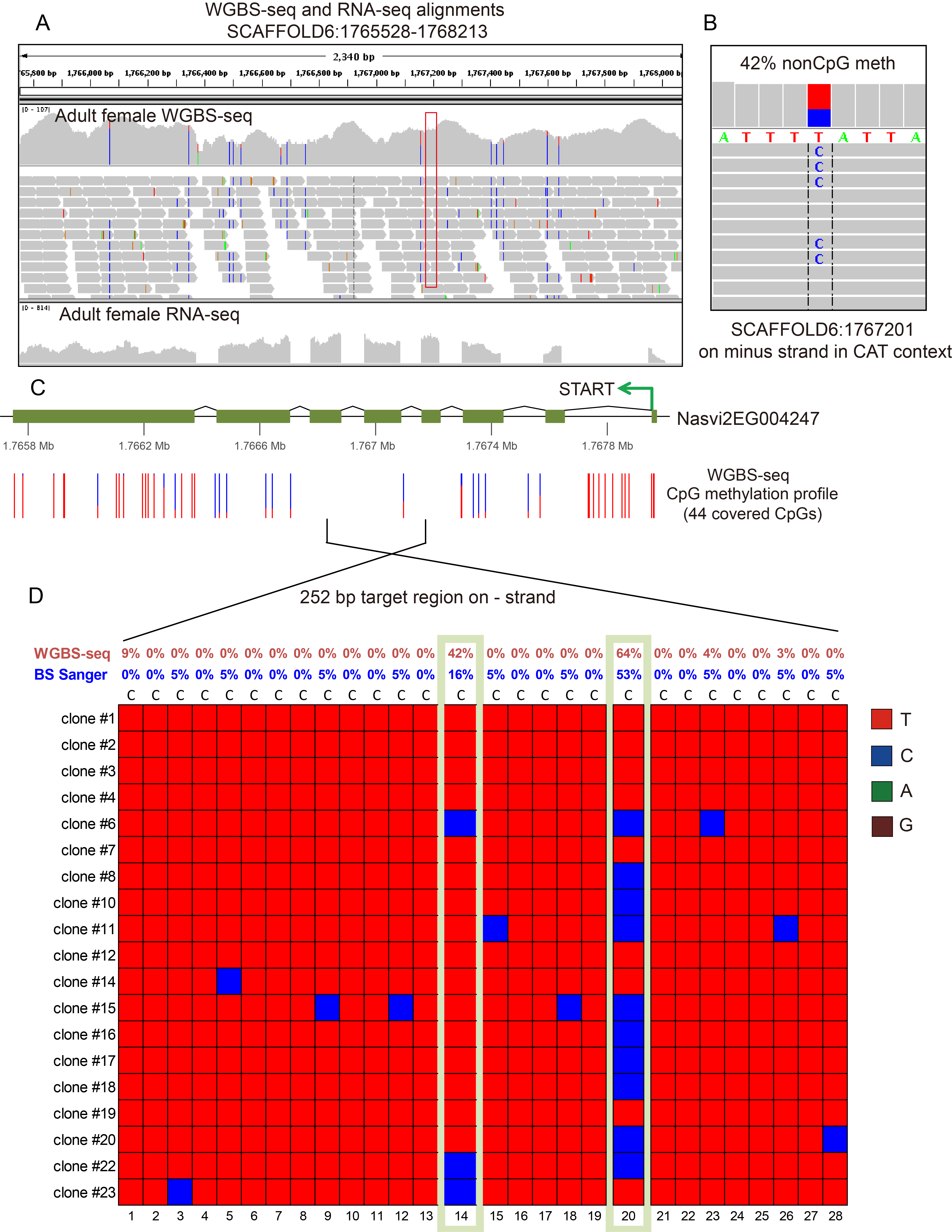

Supplement: Figure S14 — Validation of non-CpG methylation site in gene Nasvi2EG004247 in adult females. (A) IGV browser screenshot of the WGBS-seq alignments (top) and RNA-seq coverage (bottom) for Nasvi2EG004247 gene region on SCAFFOLD6 (1765528–1768213), showing the CpG sites methylation in this gene. The candidate non-CpG methylation site at position 1767201 is labeled in the red box. (B) Zoom-in view for the boxed region in (A), demonstrating that the non-CpG methylation in CAT context on the minus strand, with 42% methylated Cs estimated from the WGBS-seq reads. (C) Plots of the gene model, translation start site and CpG methylation profile for Nasvi2EG004247. A vertical bar was drawn for each CpG at its position in the gene, color-coded by the methylation percentage in proportion to the bar length (blue: methylated Cs in CpGs; red: unmethylated Cs in CpGs). There are 44 covered CpGs in the gene region. The 252 bp target region for bisulfite sequencing validation of the non-CpG methylation is labeled at the bottom. (D) Bisulfite sequencing verification results at the candidate non-CpG methylation site (site #14). The estimated methylation percentages at all C positions from the WGBS-seq and single-gene bisulfite sequencing were shown on the top. There are one CpG C (site #20) and 27 non-CpG Cs in this region. 10/19 (53%) clones have a C at the CpG C position, which is consistent with the methylation status in WGBS-seq data. Among the rest of the 27 non-CpG Cs, only the candidate non-CpG site has unconverted C in more than one clone. The non-CpG methylation at site #14 was confirmed and 3/19 (16%) clones have unconverted Cs. (TIF) [file pgen.1003872.s015.tif]

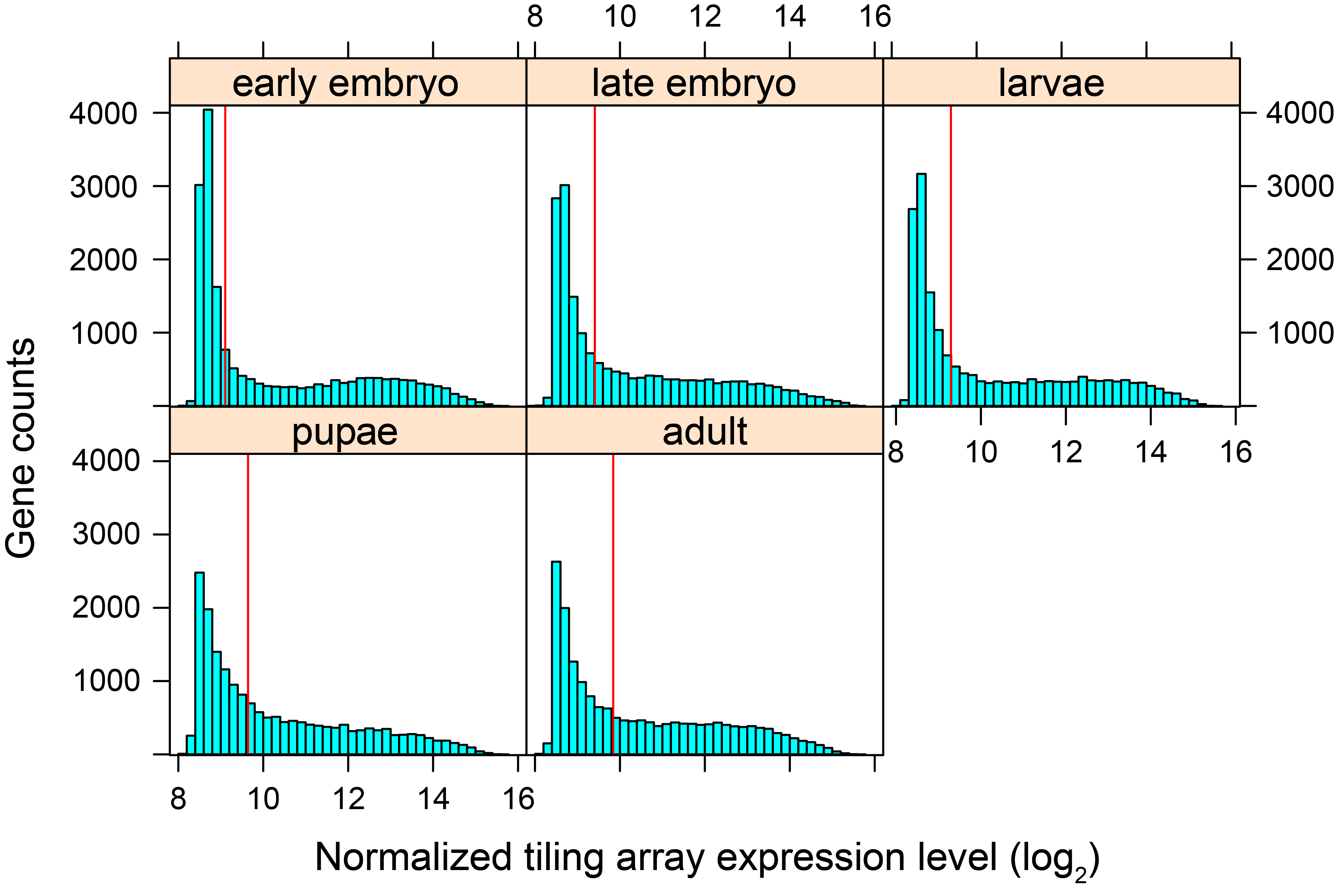

Supplement: Figure S15 — Distribution of the normalized tiling array expression values in five developmental stages. Plotted on the x-axis is the normalized tiling array expression value (log2). The y-axis is the gene count for each stage. The median expression value for each stage is labeled with the red vertical line. (TIF) [file pgen.1003872.s016.tif]

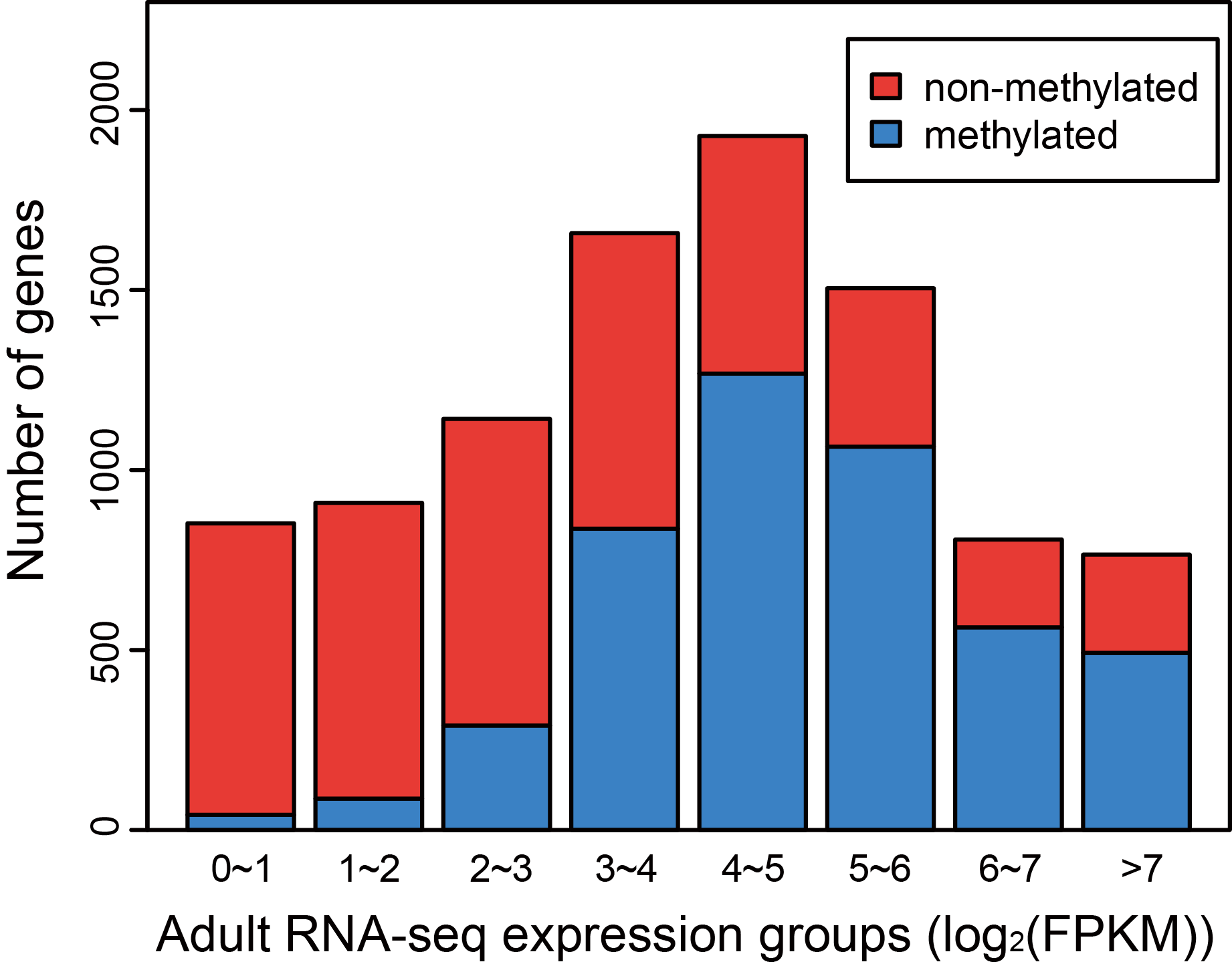

Supplement: Figure S16 — Stacked barplot for expressed methylated and non-methylated genes. Stacked barplot of methylated and non-methylated genes with adult RNA-seq expression level FPKM ≥1, binned by different expression level categories. Red: non-methylated genes; blue: methylated genes. (TIF) [file pgen.1003872.s017.tif]

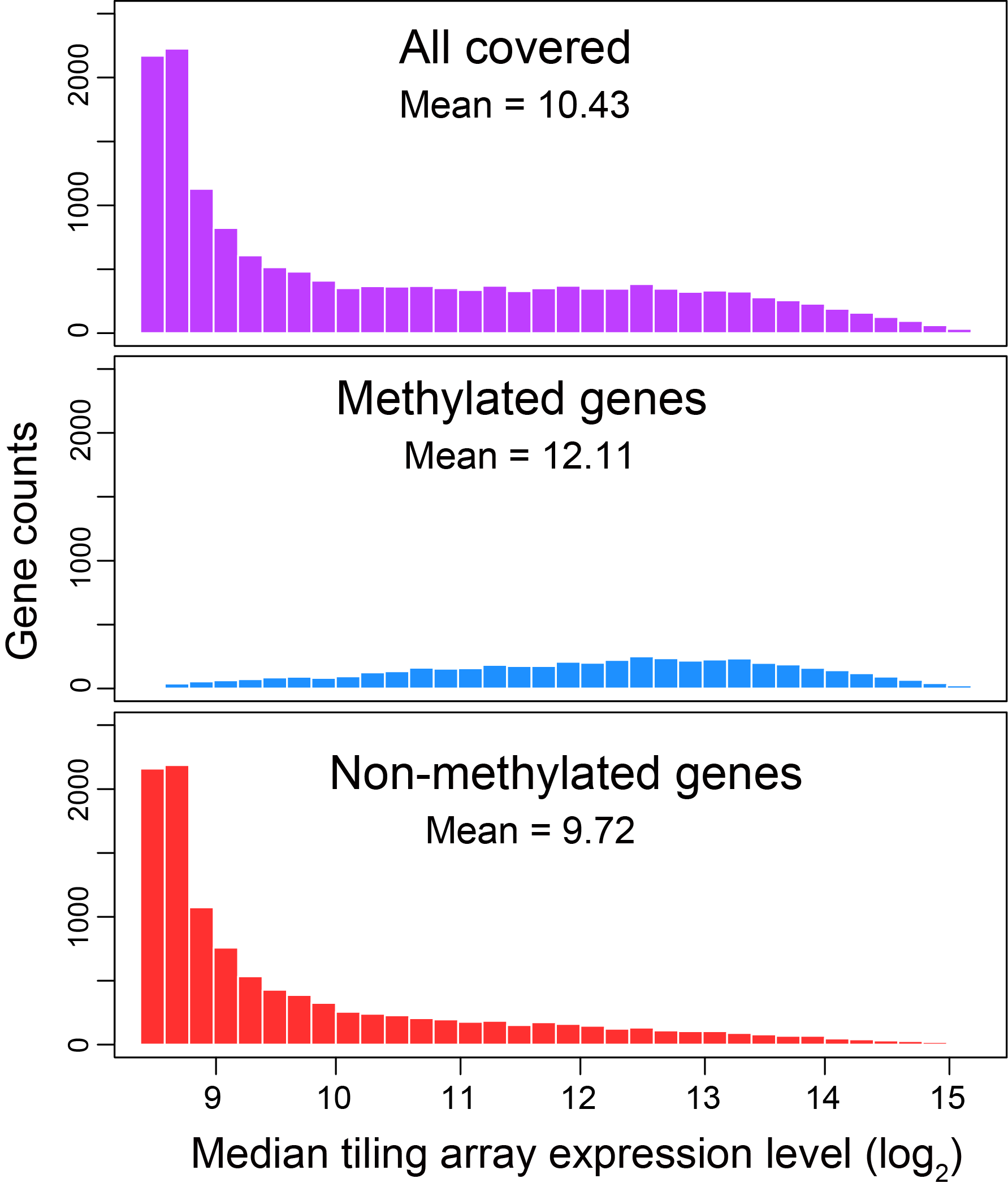

Supplement: Figure S17 — DNA methylation status and tiling array median expression level. Distribution of median tiling array expression level (log2) for methylated (blue), non-methylated (red) and all genes (purple). (TIF) [file pgen.1003872.s018.tif]

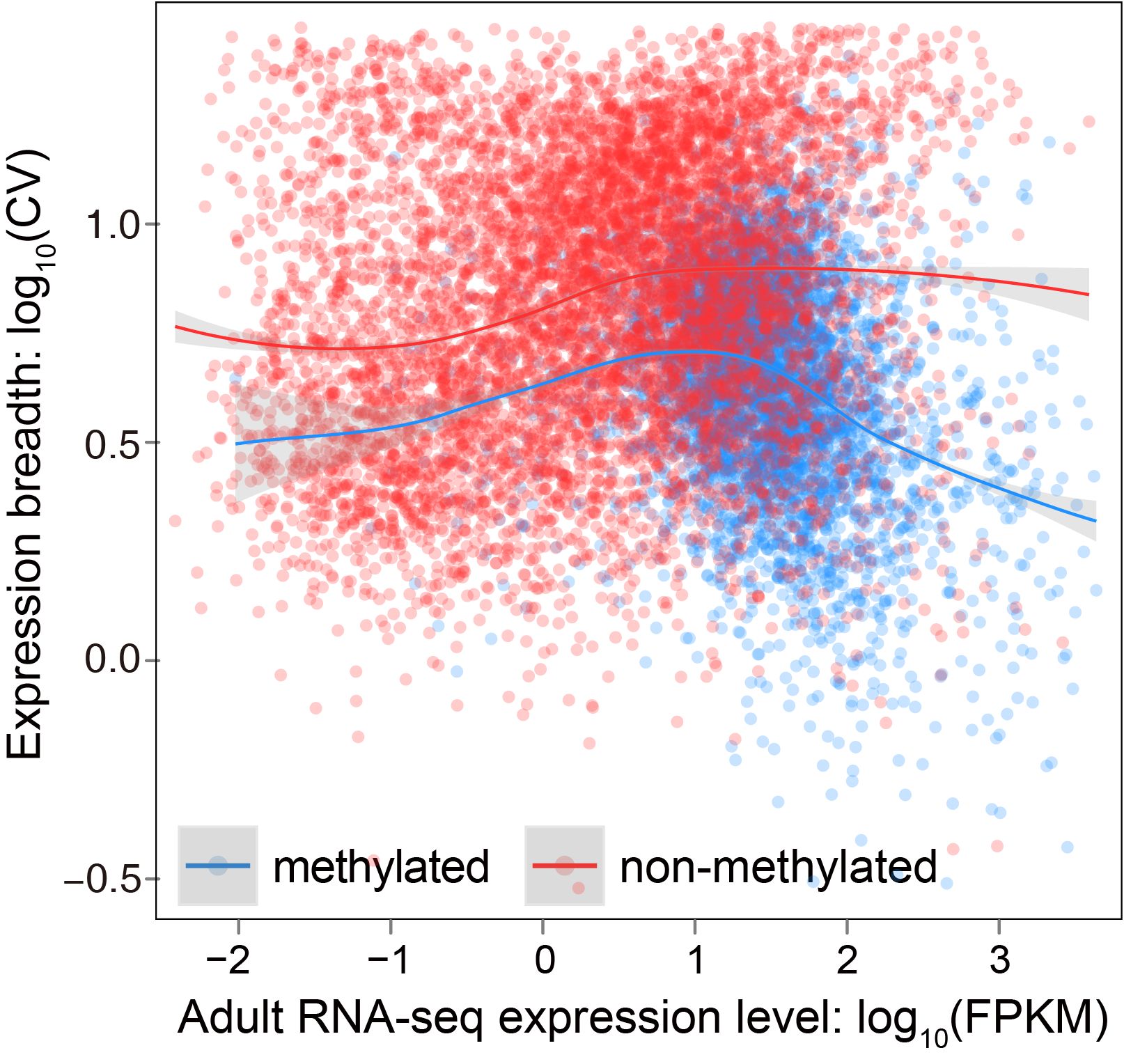

Supplement: Figure S18 — Expression breadth and the adult female RNA-seq expression level for methylation and non-methylated genes. Plotted on the y-axis is the log10 coefficient of variation (CV) for tiling array expression values in five developmental stages. On the x-axis is the RNA-seq expression level in adult female samples (log10 FPKM). The methylated genes were represented with blue dot and non-methylated genes with red dot. The fitted curve and confidence interval using non-parametric local regression for methylated and non-methylated genes were plotted in blue and red curve, respectively. (TIF) [file pgen.1003872.s019.tif]

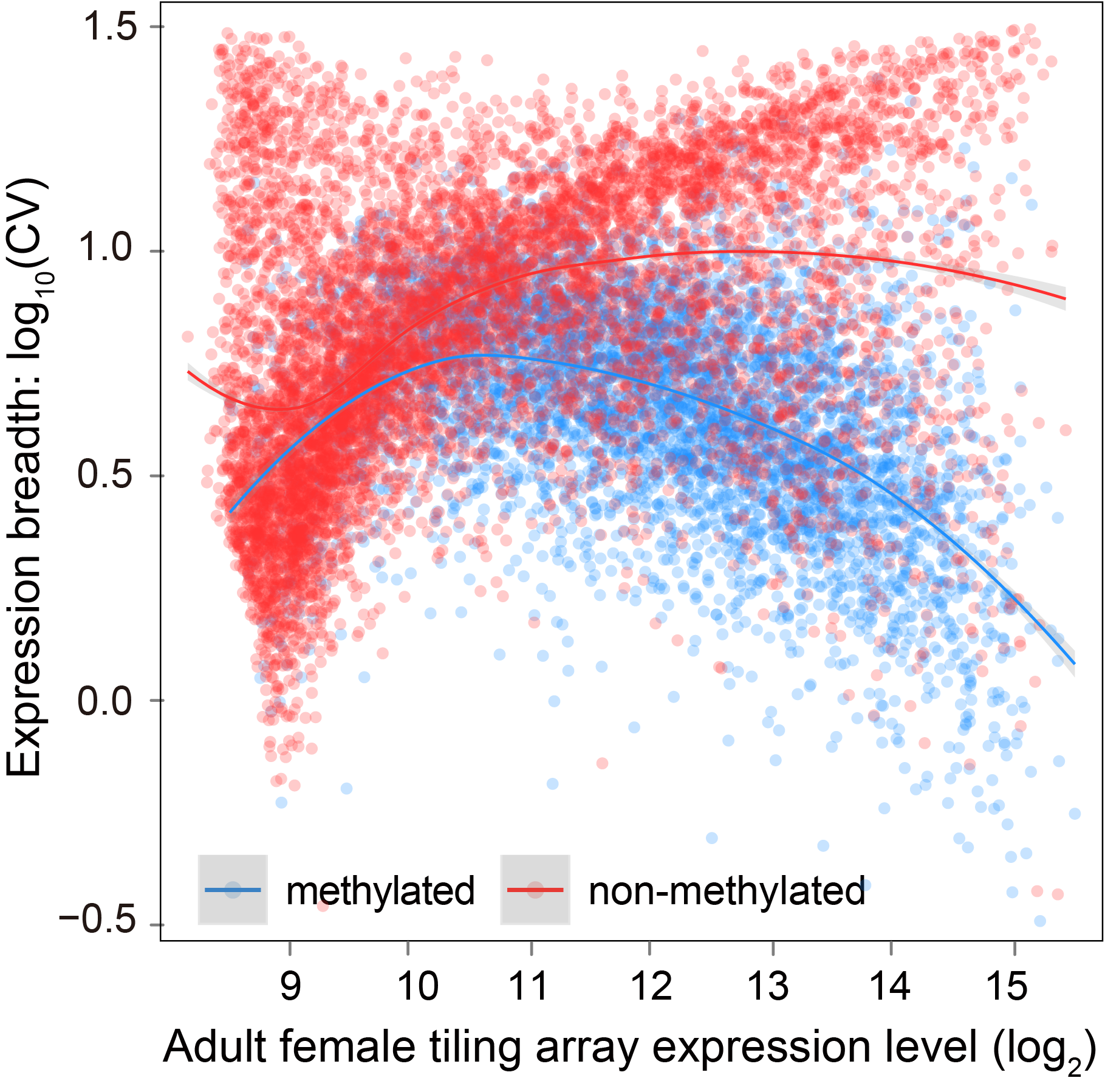

Supplement: Figure S19 — Expression breadth and the adult female tiling array expression level for methylated and non-methylated genes. Scatterplot of expression breadth (log2 expression CV) on y-axis against adult female gene expression level (log2 signal intensity) in tiling array on x-axis, color-coded by adult female methylation status (blue: methylated genes; red: non-methylated genes). Fitted lines using non-parametric local regression are shown for methylated and non-methylated genes respectively. (TIF) [file pgen.1003872.s020.tif]

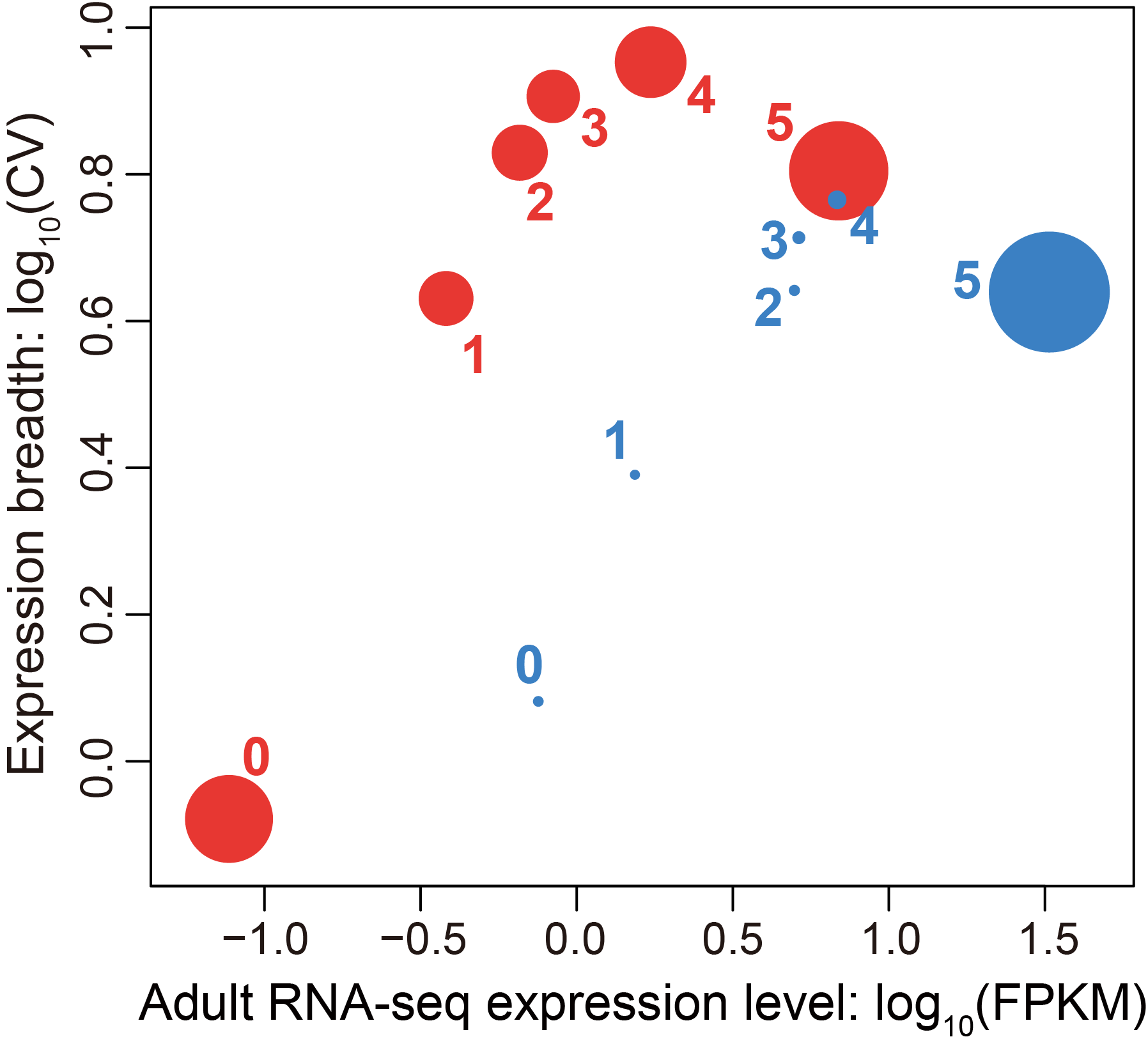

Supplement: Figure S20 — DNA methylation status and gene expression level, expression breadth and number of expressed tissues. Relationship between DNA methylation status, gene expression level, expression CV and number of expressed stages. Plotted on the y-axis is the average expression CV, and on the x-axis is the average gene expression level. Methylated (in blue) and non-methylated genes (in red) present in 0–5 developmental stages are plotted as separate round dot. The size of the area is in proportion to the number of genes in each category. (TIF) [file pgen.1003872.s021.tif]

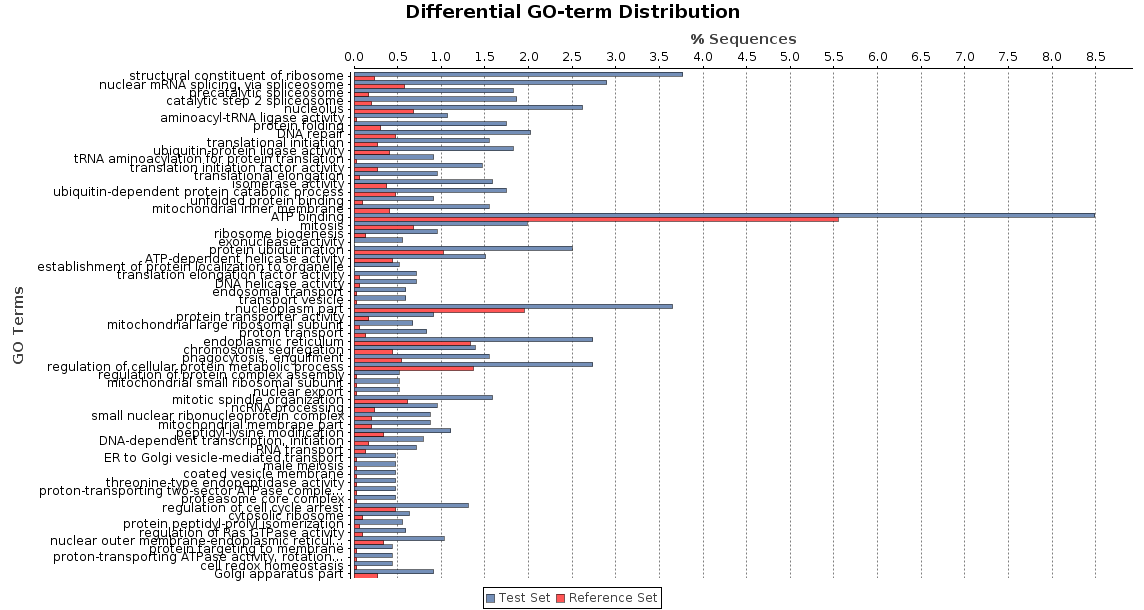

Supplement: Figure S21 — Enriched Gene Ontology categories for methylated gene in Nasonia genome. (TIF) [file pgen.1003872.s022.tif]

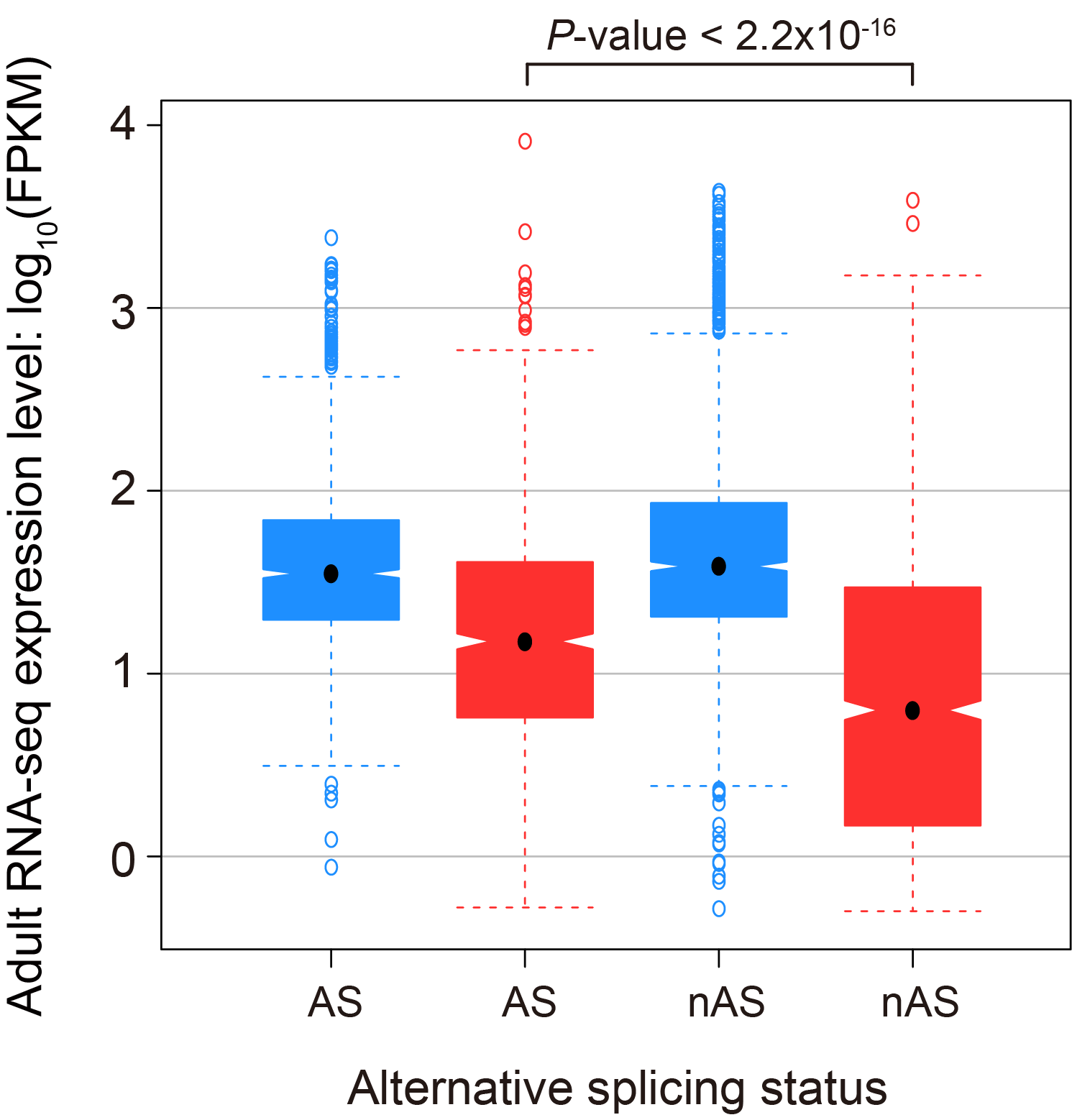

Supplement: Figure S22 — Distribution of RNA-seq expression level for the four methylation-alternative splicing classes. Plotted here is the distribution of adult female RNA-seq expression level (log10 FPKM) for alternatively spliced methylated, alternatively spliced non-methylated, non-alternatively spliced methylated, non-alternatively spliced non-methylated genes (from left to right). For methylated genes, the expression levels of alternatively spliced genes were not significantly higher than the non-alternatively spliced ones (P-value = 0.67, Kolmogorov-Smirnov test, one side). For non-methylated genes, the expression levels of alternatively spliced genes were significantly higher than the non-alternatively spliced ones (P-value<2.2×10^−16^, Kolmogorov-Smirnov test, one side). (TIF) [file pgen.1003872.s023.tif]

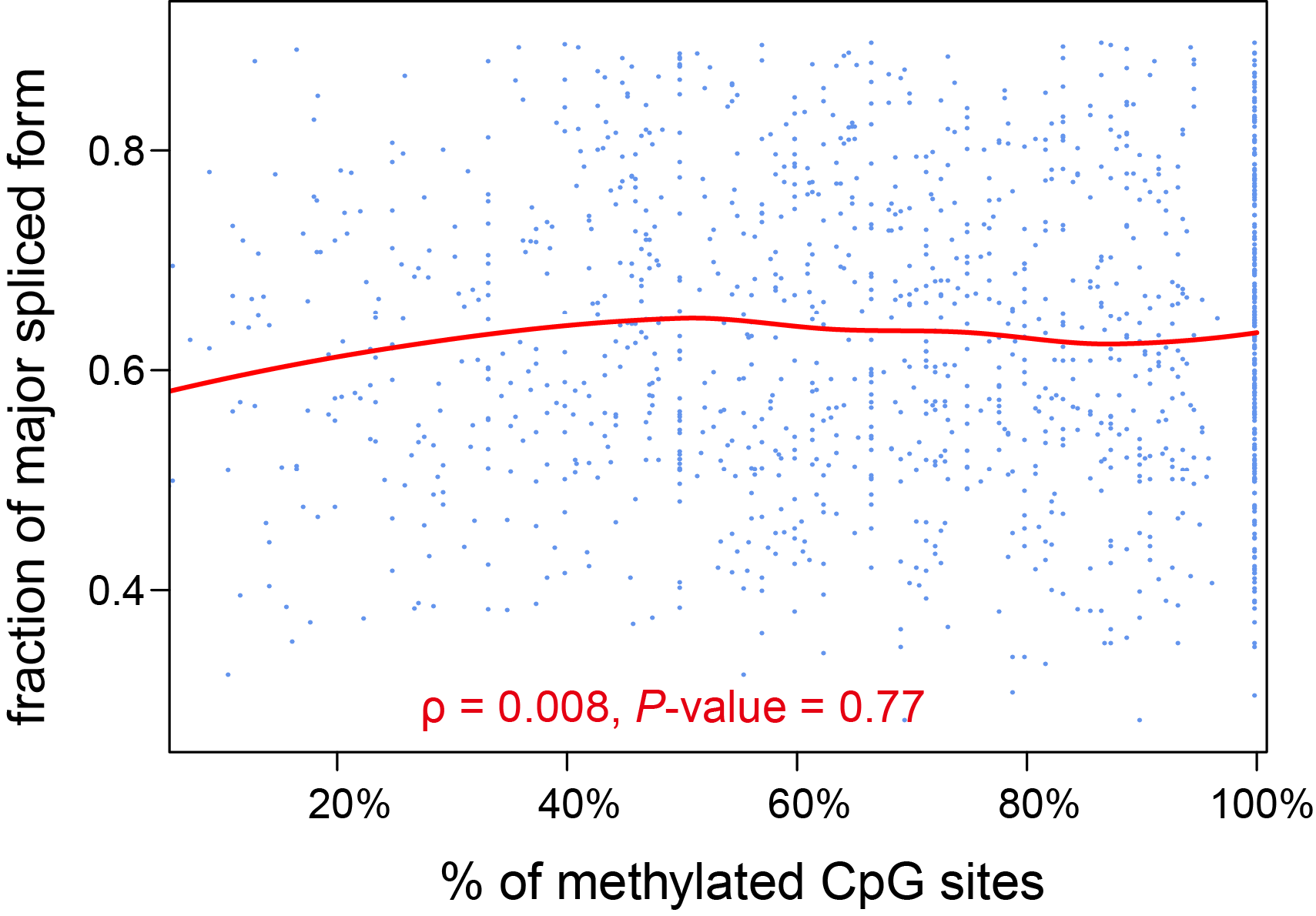

Supplement: Figure S23 — Correlation between percentage of mCpGs and fraction of major spliced form in alternatively spliced methylated genes. Scatterplot for percentage of methylated CpGs and fraction of major spliced form in alternatively spliced methylated genes. The fitted lines using non-parametric local regression are shown in red. (TIF) [file pgen.1003872.s024.tif]

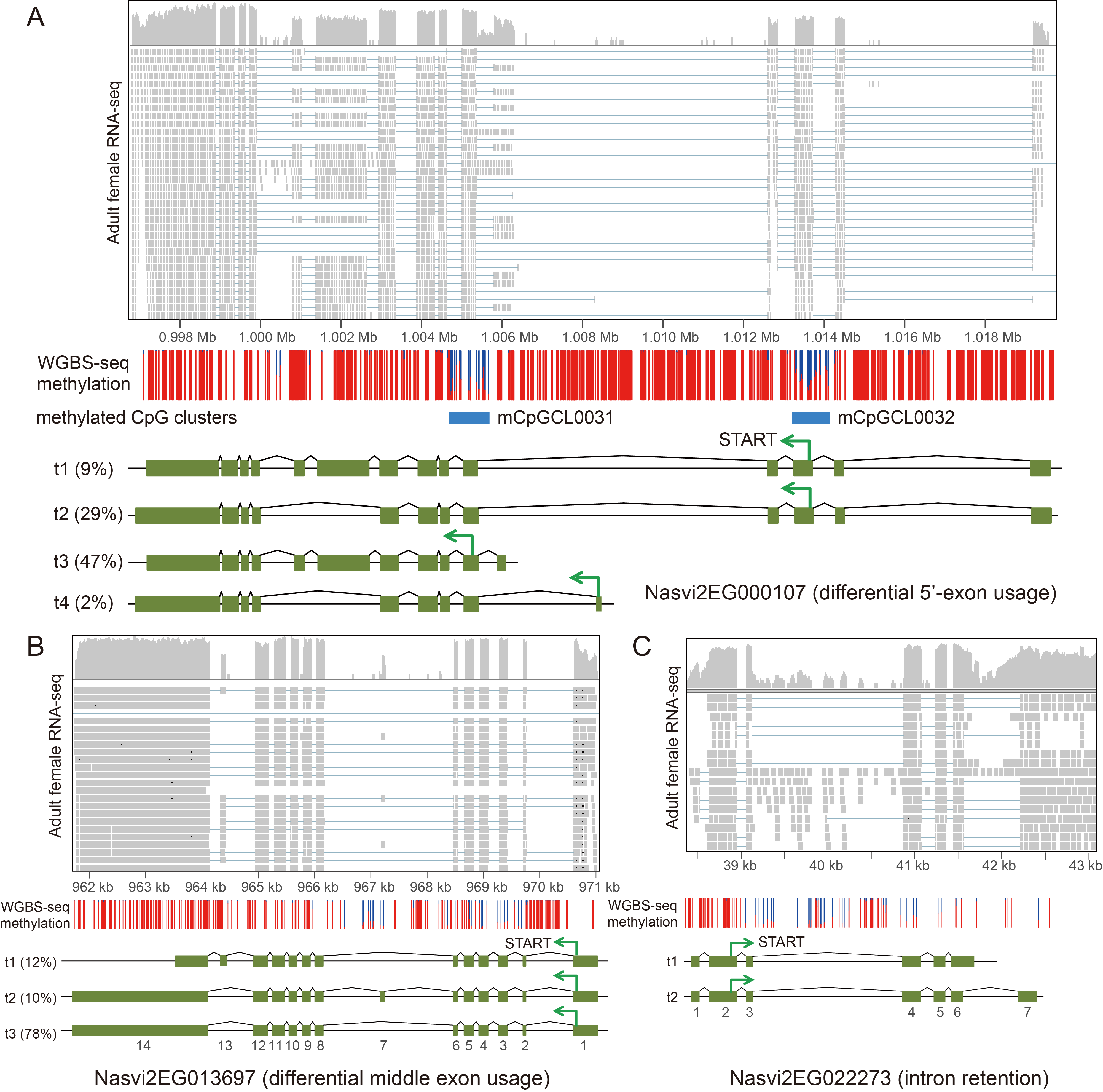

Supplement: Figure S24 — Gene expression, DNA methylation and alternative splicing profile for three methylated genes. (A) Nasvi2EG000107 showing differential 5′-exon usage. (B) Nasvi2EG013697 showing differential middle exon usage. (C) Nasvi2EG022273 showing intron retention. For each panel, plotted at the top is the IGV browser screenshot showing adult female RNA-seq coverage (on log scale) and read alignments in the gene region. Plotted at the bottom are the CpG methylation profile at covered CpG sites from WGBS-seq data and the exon model of the alternatively spliced transcripts from OGS2 gene models. The locations of methylated CpG clusters were shown as blue horizontal boxes in (A). A vertical bar was drawn for each CpG at its position in the gene, color-coded by the methylation percentage in proportion to the bar length (blue: methylated Cs; red: non-methylated Cs). OGS2 transcript variants detected in the RNA-seq data with high abundance were plotted at the bottom. The remaining minor forms were not shown in this figure. (TIF) [file pgen.1003872.s025.tif]
