# Supplementary material for: Function and Evolution of DNA Methylation in Nasonia vitripennis
Source: PLoS Genet. 2013 Oct 10;9(10):e1003872. doi: 10.1371/journal.pgen.1003872 (PMC3794928; doi:10.1371/journal.pgen.1003872)
Supplement: Table S1 — Summary of Illumina sequencing error rates estimated from lambda control DNA alignments. (DOC) [file pgen.1003872.s026.doc]

**Table S1. Summary of Illumina sequencing error rates estimated from lambda control DNA alignments.**
