# Supplementary material for: Function and Evolution of DNA Methylation in Nasonia vitripennis
Source: PLoS Genet. 2013 Oct 10;9(10):e1003872. doi: 10.1371/journal.pgen.1003872 (PMC3794928; doi:10.1371/journal.pgen.1003872)
Supplement: Table S2 — Summary for methylated and non-methylated CpGs in Nasonia genome. (DOC) [file pgen.1003872.s027.doc]

**Table S2. Summary for methylated and non-methylated CpGs in *Nasonia* genome.**

| CpG sites coverage | Counts for covered Cs | Counts for methylated Cs | # of covered CpG sites | Methylated sites | Non-methylated sites |
| --- | --- | --- | --- | --- | --- |
| >=1 | 226,856,322 | 2,972,201 (1.31%) | 12,991,754 | - | - |
| >=10 | 198,607,965 | 2,871,623 (1.45%) | 7,818,889 | 124,018 (1.59%) | 7,694,871 (98.41%) |
| >=20 | 143,174,310 | 2,492,050 (1.74%) | 3,800,233 | 84,893  (2.23%) | 3,715,340 (97.77%) |
| >=50 | 50,214,449 | 1,064,122 (2.12%) | 679,807 | 23,152  (3.41%) | 656,655  (96.59%) |
