# Supplementary material for: Function and Evolution of DNA Methylation in Nasonia vitripennis
Source: PLoS Genet. 2013 Oct 10;9(10):e1003872. doi: 10.1371/journal.pgen.1003872 (PMC3794928; doi:10.1371/journal.pgen.1003872)
Supplement: Table S3 — Methylated CpG clusters in genic region in Nasonia OGS1.2 but absent in OGS2. (DOC) [file pgen.1003872.s028.doc]

**Table S3. Methylated CpG clusters in genic region in *Nasonia* OGS1.2 but absent in OGS2.**

| mCGCL_ID | scaffold | start | end | length (bp) | percentage of mCpG | OGS1.2_ID |
| --- | --- | --- | --- | --- | --- | --- |
| mCpGCL1199 | SCAFFOLD6 | 1450502 | 1451359 | 858 | 100% | NV31640 |
| mCpGCL3087 | SCAFFOLD22 | 2101324 | 2102111 | 788 | 100% | NV21469 |
| mCpGCL3314 | SCAFFOLD26 | 1459319 | 1461389 | 2071 | 100% | NV23891 |
| mCpGCL3785 | SCAFFOLD35 | 1405659 | 1407000 | 1342 | 79% | NV16191 |
| mCpGCL4602 | SCAFFOLD101 | 504252 | 504702 | 451 | 89% | NV50511 |
| mCpGCL4603 | SCAFFOLD101 | 505231 | 505864 | 634 | 100% | NV50511 |
| mCpGCL4604 | SCAFFOLD101 | 506775 | 508928 | 2154 | 83% | NV50511 |
| mCpGCL4605 | SCAFFOLD101 | 509096 | 512651 | 3556 | 92% | NV50511 |
| mCpGCL4908 | SCAFFOLD152 | 208718 | 210498 | 1781 | 93% | NV18219 |
| mCpGCL5288 | SCAFFOLD595 | 25954 | 30792 | 4839 | 93% | NV18909 |
