# Supplementary material for: Function and Evolution of DNA Methylation in Nasonia vitripennis
Source: PLoS Genet. 2013 Oct 10;9(10):e1003872. doi: 10.1371/journal.pgen.1003872 (PMC3794928; doi:10.1371/journal.pgen.1003872)
Supplement: Table S4 — Top five methylated repetitive TE families and the adult female RNA-seq coverage. (DOC) [file pgen.1003872.s029.doc]

**Table S4. Top five methylated repetitive TE families and the adult female RNA-seq coverage.**

| TE family | No. of elements | Average Methylation level | Methylation rank | Average RNAseq coverage | Expression rank* |
| --- | --- | --- | --- | --- | --- |
| GYPSY | 12 | 44.89% | 1 | 80.5 | 51 |
| SPRINGER | 27 | 41.03% | 2 | 25.2 | 108 |
| SNAKEHEAD | 7 | 8.22% | 3 | 5902 | 1 |
| IFAC | 108 | 6.73% | 4 | 0.8 | 593 |
| BLASTOPIA | 50 | 5.93% | 5 | 8.1 | 188 |

* Among 560 TE families with average RNA-seq depth >1.
