# Supplementary material for: Function and Evolution of DNA Methylation in Nasonia vitripennis
Source: PLoS Genet. 2013 Oct 10;9(10):e1003872. doi: 10.1371/journal.pgen.1003872 (PMC3794928; doi:10.1371/journal.pgen.1003872)
Supplement: Table S5 — Summary for unconverted Cs in non-CpG context in WGBS-seq data. (DOC) [file pgen.1003872.s030.doc]

**Table S5. Summary for unconverted Cs in non-CpG context in WGBS-seq data.**

| Coverage class |  | Count of covered non-CpG sites | |  | Base count at covered nonCpG sites | | |
| --- | --- | --- | --- | --- | --- | --- | --- |
|  |  | Count | Percentage |  | T count | C count | % of unconverted Cs |
| coverage >= 1 |  | 59355138 | 83.03% |  | 629658230 | 1157722 | 0.18% |
| coverage >= 2 |  | 53843936 | 75.32% |  | 624216007 | 1145107 | 0.18% |
| coverage >= 5 |  | 38129511 | 53.34% |  | 578129450 | 1072112 | 0.19% |
| coverage >= 10 |  | 21787123 | 30.48% |  | 468849520 | 893610 | 0.19% |
| coverage >= 20 |  | 8564085 | 11.98% |  | 290074828 | 568062 | 0.20% |
