# Supplementary material for: Function and Evolution of DNA Methylation in Nasonia vitripennis
Source: PLoS Genet. 2013 Oct 10;9(10):e1003872. doi: 10.1371/journal.pgen.1003872 (PMC3794928; doi:10.1371/journal.pgen.1003872)
Supplement: Table S6 — Candidate non-CpG methylation sites with >30% unconverted Cs in non-CpG context. (DOC) [file pgen.1003872.s031.doc]

**Table S6. Candidate non-CpG methylation sites with >30% unconverted Cs in non-CpG context.**

| scaffold:position | strand | context | coverage | mC% | in CpG context? | gene | feature | CpG meth status | in mCGCLs? |
| --- | --- | --- | --- | --- | --- | --- | --- | --- | --- |
| SCAFFOLD258:14939 | + | CTA | 41 | 68% | YES | Nasvi2EG025270 | upstream | Methylated | mCpGCL5123 |
| SCAFFOLD31:1729148 | + | CAC | 11 | 64% | NO | Nasvi2EG012741 | intronic | Methylated | mCpGCL3642 |
| SCAFFOLD18:2718330 | - | CTG | 16 | 50% | YES | Nasvi2EG009629 | coding | Methylated | mCpGCL2764 |
| SCAFFOLD217:115567 | - | CAT | 12 | 50% | NO | Nasvi2EG024142 | intronic | Intron methylation | mCpGCL5073 |
| SCAFFOLD104:339451 | - | CAT | 11 | 45% | NO | Nasvi2EG019244 | intronic | Intron methylation | mCpGCL4610 (435bp upsteam) |
| SCAFFOLD18:1866842 | + | CAA | 36 | 44% | YES | Nasvi2EG009539 | coding | Methylated | mCpGCL2735 |
| SCAFFOLD3120:96 | + | CAT | 25 | 44% | YES | - | intergenic | - | - |
| SCAFFOLD384:36991 | - | CAT | 14 | 43% | NO | - | intergenic | - | mCpGCL5230 |
| SCAFFOLD166:49757 | + | CAC | 19 | 42% | NO | Nasvi2EG022369 | intronic | Methylated | mCpGCL4963 |
| SCAFFOLD3:3564053 | - | CAT | 12 | 42% | NO | Nasvi2EG002785 | intronic | Methylated | mCpGCL0736 |
| SCAFFOLD6:1767201 | - | CAT | 12 | 42% | NO | Nasvi2EG004247 | coding | Methylated | mCpGCL1206 |
| SCAFFOLD18:2719111 | + | CCT | 27 | 41% | YES | Nasvi2EG009629 | coding | Methylated | mCpGCL2764 |
| SCAFFOLD217:115710 | + | CAT | 13 | 38% | NO | Nasvi2EG024142 | intronic | Intron methylation | mCpGCL5073 |
| SCAFFOLD104:339570 | - | CAC | 16 | 38% | NO | Nasvi2EG019244 | intronic | Intron methylation | mCpGCL4610 (316bp upsteam) |
| SCAFFOLD11:525892 | - | CTC | 11 | 36% | NO | - | intergenic | - | - |
| SCAFFOLD316:13840 | - | CAA | 11 | 36% | NO | Nasvi2EG026653 | intronic | Methylated | mCpGCL5180 |
| SCAFFOLD109:201166 | - | CAC | 14 | 36% | NO | Nasvi2EG019523 | intronic | Methylated | mCpGCL4632 |
| SCAFFOLD18:1867777 | + | CAC | 17 | 35% | YES | Nasvi2EG009539 | coding | Methylated | mCpGCL2735 |
| SCAFFOLD18:2350316 | + | CTT | 26 | 35% | YES | Nasvi2EG009571 | intronic | Non-methylated | - |
| SCAFFOLD1:8780626 | + | CAC | 12 | 33% | NO | Nasvi2EG001107 | intronic | Methylated | mCpGCL0336 (1.16kb upsteam) |
| SCAFFOLD15:2089425 | + | CTC | 12 | 33% | NO | Nasvi2EG008244 | 3'-UTR | Methylated | mCpGCL2355 |
| SCAFFOLD18:385941 | + | CTC | 15 | 33% | NO | Nasvi2EG009383 | coding | Methylated | mCpGCL2702 |
| SCAFFOLD24:146293 | - | CAT | 12 | 33% | NO | Nasvi2EG011228 | intronic | Methylated | mCpGCL3170 |
| SCAFFOLD35:1496661 | + | CAT | 18 | 33% | NO | Nasvi2EG013324 | 3'-UTR | Methylated | mCpGCL3793 |
| SCAFFOLD501:53481 | - | CAG | 15 | 33% | NO | Nasvi2EG029345 | intronic | Non-methylated | - |
| SCAFFOLD5:2276640 | + | CTC | 19 | 32% | YES | Nasvi2EG003837 | coding | Methylated | mCpGCL1085 |
| SCAFFOLD384:36993 | - | CAC | 13 | 31% | NO | - | intergenic | - | mCpGCL5230 |
| SCAFFOLD2210:2037 | + | CTA | 13 | 31% | NO | Nasvi2EG034914 | intronic | Non-methylated | - |
