# Supplementary material for: Function and Evolution of DNA Methylation in Nasonia vitripennis
Source: PLoS Genet. 2013 Oct 10;9(10):e1003872. doi: 10.1371/journal.pgen.1003872 (PMC3794928; doi:10.1371/journal.pgen.1003872)
Supplement: Table S7 — Logistic regression analysis results for methylation status with gene expression and expression breadth as predictors. (DOC) [file pgen.1003872.s032.doc]

**Table S7. Logistic regression analysis results for methylation status with gene expression and expression breadth as predictors.**

Full model: methylation staus ~ logAdultexp + logCV

| **Model fit statistics** | | | | | | | | | | | | | |
| --- | --- | --- | --- | --- | --- | --- | --- | --- | --- | --- | --- | --- | --- |
| Criterion | | Intercept only | | | | | Intercept & Covariance | | | | | | |
| AIC | | 20265.687 | | | | | 12624.108 | | | | | | |
| SC(BIC) | | 20273.443 | | | | | 12647.377 | | | | | | |
| -2logL | | 20263.687 | | | | | 12618.108 | | | | | | |
| R^2^: 0.3578 Max-rescaled R^2^: 0.5180 | | | | | | | | | | | | | |
| **Testing Global Null Hypothesis: Beta = 0** | | | | | | | | | | | | | |
| Test | | | | Chi-Square | | | | | DF | | Pr > ChiSq | | |
| Likelihood Ratio | | | | 7645.5785 | | | | | 2 | | <.0001 | | |
| Score | | | | 7040.4203 | | | | | 2 | | <.0001 | | |
| Wald | | | | 4291.8616 | | | | | 2 | | <.0001 | | |
| **Association of Predicted Probabilities and Observed Responses** | | | | | | | | | | | | | |
| Percent Concordance | | | | | | 90.2 | | | | Somers’ D | | | 0.805 |
| Percent Discordant | | | | | | 9.7 | | | | Gamma | | | 0.806 |
| Percent Tied | | | | | | 0.1 | | | | Tau-a | | | 0.32 |
| Pairs | | | | | | 59242050 | | | | c | | | 0.903 |
| **Analysis of Maximum Likelihood Estimates** | | | | | | | | | | | | | |
| Parameter | DF | | Estimate | | Standard Error | | | Wald Chi-Square | | | | Pr > ChiSq | |
| Intercept | 1 | | -2.2408 | | 0.0643 | | | 1215.4356 | | | | <.0001 | |
| logAdultexp | 1 | | 2.6781 | | 0.0416 | | | 4141.5313 | | | | <.0001 | |
| logCV | 1 | | -1.6972 | | 0.089 | | | 363.5868 | | | | <.0001 | |
