# Supplementary material for: Function and Evolution of DNA Methylation in Nasonia vitripennis
Source: PLoS Genet. 2013 Oct 10;9(10):e1003872. doi: 10.1371/journal.pgen.1003872 (PMC3794928; doi:10.1371/journal.pgen.1003872)
Supplement: Table S8 — Logistic regression analysis for methylation status with gene expression or expression breadth as single predictor. (DOC) [file pgen.1003872.s033.doc]

**Table S8. Logistic regression analysis for methylation status with gene expression or expression breadth as single predictor.**

Model 1: methylation staus ~ logAdultexp

| **Model fit statistics** | | | | | | | | | | |
| --- | --- | --- | --- | --- | --- | --- | --- | --- | --- | --- |
| Criterion | Intercept only | | | Intercept & Covariance | | | | | | |
| AIC | 20265.687 | | | 13012.978 | | | | | | |
| SC(BIC) | 20273.443 | | | 13028.491 | | | | | | |
| -2logL | 20263.687 | | | 13008.978 | | | | | | |
| R^2^: 0.3431 Max-rescaled R^2^: 0.4967 | | | | | | | | | | |
| **Testing Global Null Hypothesis: Beta = 0** | | | | | | | | | |  |
| Test | | Chi-Square | | | DF | | Pr > ChiSq | | |  |
| Likelihood Ratio | | 7254.7082 | | | 1 | | <.0001 | | |  |
| Score | | 6730.8413 | | | 1 | | <.0001 | | |  |
| Wald | | 4196.6274 | | | 1 | | <.0001 | | |  |
| **Association of Predicted Probabilities and Observed Responses** | | | | | | | | |  | |
| Percent Concordance | | | 89.3 | | | Somers’ D | | 0.788 |  | |
| Percent Discordant | | | 10.5 | | | Gamma | | 0.789 |  | |
| Percent Tied | | | 0.1 | | | Tau-a | | 0.313 |  | |
| Pairs | | | 59242050 | | | c | | 0.894 |  | |

Model 2: methylation staus ~ logCV

| **Model fit statistics** | | | | | | | | | |  |
| --- | --- | --- | --- | --- | --- | --- | --- | --- | --- | --- |
| Criterion | Intercept only | | | Intercept & Covariance | | | | | |  |
| AIC | 20265.687 | | | 20186.969 | | | | | |  |
| SC(BIC) | 20273.443 | | | 20202.481 | | | | | |  |
| -2logL | 20263.687 | | | 20182.969 | | | | | |  |
| R^2^: 0.0047 Max-rescaled R^2^: 0.0068 | | | | | | | | | |  |
| **Testing Global Null Hypothesis: Beta = 0** | | | | | | | | | | |
| Test | | Chi-Square | | | | DF | Pr > ChiSq | | | |
| Likelihood Ratio | | 80.7177 | | | | 1 | <.0001 | | | |
| Score | | 80.7959 | | | | 1 | <.0001 | | | |
| Wald | | 80.4576 | | | | 1 | <.0001 | | | |
| **Association of Predicted Probabilities and Observed Responses** | | | | | | | | |  | |
| Percent Concordance | | | 54.7 | | Somers’ D | | | 0.11 |  | |
| Percent Discordant | | | 43.6 | | Gamma | | | 0.112 |  | |
| Percent Tied | | | 1.7 | | Tau-a | | | 0.044 |  | |
| Pairs | | | 59242050 | | c | | | 0.555 |  | |
