# Supplementary material for: Function and Evolution of DNA Methylation in Nasonia vitripennis
Source: PLoS Genet. 2013 Oct 10;9(10):e1003872. doi: 10.1371/journal.pgen.1003872 (PMC3794928; doi:10.1371/journal.pgen.1003872)
Supplement: Table S9 — Mean/median expression CV for methylated and non-methylated genes in median array expression level categories. (DOC) [file pgen.1003872.s034.doc]

**Table S9. Mean/median expression CV for methylated and non-methylated genes in median array expression level categories.**

| Tiling array median expression | # of genes | mean exp. CV | |  | median exp. CV | |  | mean # exp.stages | |  |
| --- | --- | --- | --- | --- | --- | --- | --- | --- | --- | --- |
|  |  | meth | non-meth |  | meth | non-meth |  | meth | non-meth |  |
| 9-11 | 4642 | 6.44 | 8.57 |  | 6.05 | 7.02 |  | 4.81 | 4.15 |  |
| 11-13 | 3508 | 5.54 | 10.13 |  | 5.25 | 9.18 |  | 5.00 | 4.73 |  |
| >13 | 2101 | 3.56 | 8.45 |  | 3.20 | 5.79 |  | 5.00 | 4.87 |  |
