# Supplementary material for: Function and Evolution of DNA Methylation in Nasonia vitripennis
Source: PLoS Genet. 2013 Oct 10;9(10):e1003872. doi: 10.1371/journal.pgen.1003872 (PMC3794928; doi:10.1371/journal.pgen.1003872)
Supplement: Table S10 — Twenty most significant GO terms enriched amongst all methylated Nasonia genes. (DOC) [file pgen.1003872.s035.doc]

## Table S10: Twenty most significant GO terms enriched amongst all methylated *Nasonia* genes.

| **GO-ID** | **Term** | **Category*** | **P-Value** | **FDR** |
| --- | --- | --- | --- | --- |
| GO:0003735 | structural constituent of ribosome | F | 2.0E-24 | 8.0E-22 |
| GO:0000398 | nuclear mRNA splicing, via spliceosome | P | 8.3E-12 | 1.5E-9 |
| GO:0071011 | precatalytic spliceosome | C | 4.1E-11 | 6.9E-9 |
| GO:0071013 | catalytic step 2 spliceosome | C | 1.0E-10 | 1.6E-8 |
| GO:0005730 | Nucleolus | C | 6.6E-9 | 8.4E-7 |
| GO:0004812 | aminoacyl-tRNA ligase activity | F | 1.4E-8 | 1.6E-6 |
| GO:0006457 | protein folding | P | 3.4E-8 | 3.5E-6 |
| GO:0006281 | DNA repair | P | 1.0E-7 | 9.5E-6 |
| GO:0006413 | translational initiation | P | 2.2E-7 | 1.9E-5 |
| GO:0004842 | ubiquitin-protein ligase activity | F | 2.4E-7 | 2.0E-5 |
| GO:0006418 | tRNA aminoacylation for protein translation | P | 2.6E-7 | 2.1E-5 |
| GO:0003743 | translation initiation factor activity | F | 7.1E-7 | 5.3E-5 |
| GO:0006414 | translational elongation | P | 9.1E-7 | 6.6E-5 |
| GO:0016853 | isomerase activity | F | 2.6E-6 | 1.7E-4 |
| GO:0006511 | ubiquitin-dependent protein catabolic process | P | 4.2E-6 | 2.6E-4 |
| GO:0051082 | unfolded protein binding | F | 8.9E-6 | 5.3E-4 |
| GO:0005743 | mitochondrial inner membrane | C | 1.0E-5 | 6.0E-4 |
| GO:0005524 | ATP binding | F | 1.3E-5 | 7.5E-4 |
| GO:0007067 | Mitosis | P | 1.7E-5 | 9.2E-4 |
| GO:0042254 | ribosome biogenesis | P | 1.8E-5 | 9.9E-4 |

*F=Molecular function C = cellular component P= Biological process
