# Supplementary material for: Function and Evolution of DNA Methylation in Nasonia vitripennis
Source: PLoS Genet. 2013 Oct 10;9(10):e1003872. doi: 10.1371/journal.pgen.1003872 (PMC3794928; doi:10.1371/journal.pgen.1003872)
Supplement: Table S11 — Enriched GO terms amongst methylated genes with median array expression levels 9–11 (low expression). (DOC) [file pgen.1003872.s036.doc]

## Table S11: Enriched GO terms amongst methylated genes with median array expression levels 9-11 (low expression).

| **GO-ID** | **Term** | **Category*** | **P-Value** | **FDR** |
| --- | --- | --- | --- | --- |
| GO:0044267 | cellular protein metabolic process | P | 1.2E-10 | 6.5E-7 |
| GO:0004386 | helicase activity | F | 2.4E-9 | 6.2E-6 |
| GO:0006412 | translation | P | 4.0E-9 | 6.9E-6 |
| GO:0030529 | ribonucleoprotein complex | C | 6.6E-9 | 8.7E-6 |
| GO:0044260 | cellular macromolecule metabolic process | P | 4.1E-8 | 4.3E-5 |
| GO:0019538 | protein metabolic process | P | 7.2E-8 | 6.3E-5 |
| GO:0044237 | cellular metabolic process | P | 8.9E-8 | 6.7E-5 |
| GO:0044424 | intracellular part | C | 1.1E-7 | 6.9E-5 |
| GO:0005622 | intracellular | C | 1.8E-7 | 1.0E-4 |
| GO:0006396 | RNA processing | P | 3.9E-6 | 2.0E-3 |

*F=Molecular function C = cellular component P= Biological process
