# Supplementary material for: Function and Evolution of DNA Methylation in Nasonia vitripennis
Source: PLoS Genet. 2013 Oct 10;9(10):e1003872. doi: 10.1371/journal.pgen.1003872 (PMC3794928; doi:10.1371/journal.pgen.1003872)
Supplement: Table S12 — Enriched GO terms amongst methylated genes with median array expression levels 11–13 (medium expression). (DOC) [file pgen.1003872.s037.doc]

## Table S12: Enriched GO terms amongst methylated genes with median array expression levels 11-13 (medium expression).

| **GO-ID** | **Term** | **Category*** | **P-Value** | **FDR** |
| --- | --- | --- | --- | --- |
| GO:0044424 | intracellular part | C | 1.7E-15 | 9.0E-12 |
| GO:0005622 | intracellular | C | 2.0E-14 | 5.1E-11 |
| GO:0044260 | cellular macromolecule metabolic process | P | 2.0E-13 | 3.5E-10 |
| GO:0043229 | intracellular organelle | C | 3.6E-12 | 3.8E-9 |
| GO:0043226 | organelle | C | 3.6E-12 | 3.8E-9 |
| GO:0043170 | macromolecule metabolic process | P | 4.4E-12 | 3.8E-9 |
| GO:0044446 | intracellular organelle part | C | 8.8E-12 | 6.4E-9 |
| GO:0044422 | organelle part | C | 9.9E-12 | 6.4E-9 |
| GO:0043227 | membrane-bounded organelle | C | 1.7E-11 | 9.5E-9 |
| GO:0043231 | intracellular membrane-bounded organelle | C | 2.1E-11 | 1.1E-8 |

*F=Molecular function C = cellular component P= Biological process
