# Supplementary material for: Function and Evolution of DNA Methylation in Nasonia vitripennis
Source: PLoS Genet. 2013 Oct 10;9(10):e1003872. doi: 10.1371/journal.pgen.1003872 (PMC3794928; doi:10.1371/journal.pgen.1003872)
Supplement: Table S13 — Enriched GO terms amongst methylated genes with median array expression levels 13–15 (high expression). (DOC) [file pgen.1003872.s038.doc]

## Table S13: Enriched GO terms amongst methylated genes with median array expression levels 13-15 (high expression).

| **GO-ID** | **Term** | **Category*** | **P-Value** | **FDR** |
| --- | --- | --- | --- | --- |
| GO:0044424 | intracellular part | C | 3.6E-8 | 1.2E-4 |
| GO:0044260 | cellular macromolecule metabolic process | P | 1.1E-7 | 1.2E-4 |
| GO:0044267 | cellular protein metabolic process | P | 1.2E-7 | 1.2E-4 |
| GO:0005622 | intracellular | C | 1.3E-7 | 1.2E-4 |
| GO:0019538 | protein metabolic process | P | 1.4E-7 | 1.2E-4 |
| GO:0032991 | macromolecular complex | C | 2.4E-7 | 1.7E-4 |
| GO:0043226 | organelle | C | 2.9E-7 | 1.7E-4 |
| GO:0043229 | intracellular organelle | C | 4.1E-7 | 2.1E-4 |
| GO:0043170 | macromolecule metabolic process | P | 5.2E-7 | 2.4E-4 |
| GO:0009059 | macromolecule biosynthetic process | P | 9.5E-7 | 3.3E-4 |

*F=Molecular function C = cellular component P= Biological process
