# Supplementary material for: Function and Evolution of DNA Methylation in Nasonia vitripennis
Source: PLoS Genet. 2013 Oct 10;9(10):e1003872. doi: 10.1371/journal.pgen.1003872 (PMC3794928; doi:10.1371/journal.pgen.1003872)
Supplement: Table S14 — Methylated genes containing two or more methylated CpG clusters (mCGCLs) with differential 5′exon usage. (DOC) [file pgen.1003872.s039.doc]

**Table S14. Methylated genes containing two or more methylated CpG clusters (mCGCLs) with differential 5’exon usage.**

| No. | OGS2 gene ID | Scaffold | number of mCGCLs | list of mCGCLs |
| --- | --- | --- | --- | --- |
| 1 | Nasvi2EG023365 | SCAFFOLD193 | 2 | mCpGCL5026, mCpGCL5027 |
| 2 | Nasvi2EG004924 | SCAFFOLD7 | 2 | mCpGCL1415, mCpGCL1416 |
| 3 | Nasvi2EG000107 | SCAFFOLD1 | 2 | mCpGCL0031, mCpGCL0032 |
| 4 | Nasvi2EG008793 | SCAFFOLD16 | 3 | mCpGCL2504, mCpGCL2505, mCpGCL2506 |
| 5 | Nasvi2EG006266 | SCAFFOLD10 | 4 | mCpGCL1774, mCpGCL1775, mCpGCL1776, mCpGCL1777 |
| 6 | Nasvi2EG014348 | SCAFFOLD42 | 2 | mCpGCL4063, mCpGCL4064 |
| 7 | Nasvi2EG021450 | SCAFFOLD144 | 3 | mCpGCL4892, mCpGCL4893, mCpGCL4894 |
| 8 | Nasvi2EG010422 | SCAFFOLD21 | 2 | mCpGCL2940, mCpGCL2941 |
| 9 | Nasvi2EG009340 | SCAFFOLD18 | 3 | mCpGCL2680, mCpGCL2681, mCpGCL2682 |
| 10 | Nasvi2EG014059 | SCAFFOLD40 | 2 | mCpGCL3991, mCpGCL3992 |
| 11 | Nasvi2EG002051 | SCAFFOLD2 | 3 | mCpGCL0526, mCpGCL0527, mCpGCL0528 |
| 12 | Nasvi2EG018614 | SCAFFOLD94 | 2 | mCpGCL4585, mCpGCL4586 |
| 13 | Nasvi2EG006312 | SCAFFOLD10 | 2 | mCpGCL1781, mCpGCL1782 |
| 14 | Nasvi2EG006410 | SCAFFOLD10 | 3 | mCpGCL1797, mCpGCL1798, mCpGCL1799 |
| 15 | Nasvi2EG011335 | SCAFFOLD24 | 2 | mCpGCL3213, mCpGCL3214 |
| 16 | Nasvi2EG001012 | SCAFFOLD1 | 2 | mCpGCL0282, mCpGCL0283 |
| 17 | Nasvi2EG021063 | SCAFFOLD136 | 2 | mCpGCL4850, mCpGCL4851 |
| 18 | Nasvi2EG028418 | SCAFFOLD418 | 2 | mCpGCL5247, mCpGCL5248 |
| 19 | Nasvi2EG006696 | SCAFFOLD11 | 2 | mCpGCL1898, mCpGCL1899 |
| 20 | Nasvi2EG000104 | SCAFFOLD1 | 2 | mCpGCL0027, mCpGCL0028 |
| 21 | Nasvi2EG003286 | SCAFFOLD4 | 2 | mCpGCL0897, mCpGCL0898 |
| 22 | Nasvi2EG000163 | SCAFFOLD1 | 2 | mCpGCL0058, mCpGCL0059 |
| 23 | Nasvi2EG011268 | SCAFFOLD24 | 2 | mCpGCL3190, mCpGCL3191 |
| 24 | Nasvi2EG004109 | SCAFFOLD6 | 2 | mCpGCL1175, mCpGCL1176 |
| 25 | Nasvi2EG006789 | SCAFFOLD11 | 2 | mCpGCL1940, mCpGCL1941 |
| 26 | Nasvi2EG011276 | SCAFFOLD24 | 2 | mCpGCL3195, mCpGCL3196 |
| 27 | Nasvi2EG015483 | SCAFFOLD51 | 2 | mCpGCL4269, mCpGCL4270 |
| 28 | Nasvi2EG004772 | SCAFFOLD7 | 3 | mCpGCL1361, mCpGCL1362, mCpGCL1363 |
