# Supplementary material for: Function and Evolution of DNA Methylation in Nasonia vitripennis
Source: PLoS Genet. 2013 Oct 10;9(10):e1003872. doi: 10.1371/journal.pgen.1003872 (PMC3794928; doi:10.1371/journal.pgen.1003872)
Supplement: Table S15 — Statistical significance of expression breadth difference between methylated and non-methylated genes in three conservation categories. (DOC) [file pgen.1003872.s040.doc]

**Table S15. Statistical significance of expression breadth difference between methylated and non-methylated genes in three conservation categories.**

|  | **All species** | |  | **Hymenoptera** | |  | ***Nasonia*-only** | |
| --- | --- | --- | --- | --- | --- | --- | --- | --- |
|  | methylated | non-methylated |  | methylated | non-methylated |  | methylated | non-methylated |
| **All species**  methylated | - | *P*< 2.2x10^-16^* |  | *P* = 2.7x10^-5^ | *P* < 2.2x10^-16^ |  | *P*= 8.9x10^-11^ | *P* < 2.2x10^-16^ |
| **All species** non-methylated | - | - |  | *P* = 4.0x10^-11^ | *P* = 4.5x10^-16^ |  | *P*= 0.0028 | *P* < 2.2x10^-16^ |
| **Hymenoptera** methylated | - | - |  | - | *P* < 2.2x10^-16^ |  | *P* = 0.106 | *P* < 2.2x10^-16^ |
| **Hymenoptera**  non-methylated | - | - |  | - | - |  | *P* = 2.2x10^-8^ | *P* = 3.9x10^-5^ |
| **Nasonia only**  methylated | - | - |  | - | - |  | - | *P* = 2.5x10^-11^ |
| **Nasonia only**  non-methylated | - | - |  | - | - |  | - | - |

## *: Expression breadth was measured by number of expressed stages. Mann-Whitney U Test significance was reported here.
