# Supplementary material for: Function and Evolution of DNA Methylation in Nasonia vitripennis
Source: PLoS Genet. 2013 Oct 10;9(10):e1003872. doi: 10.1371/journal.pgen.1003872 (PMC3794928; doi:10.1371/journal.pgen.1003872)
Supplement: Table S16 — Ten most significant enriched GO terms for conserved genes methylated in both Nasonia and Apis. (DOC) [file pgen.1003872.s041.doc]

## **Table S16: Ten most significant enriched GO terms for conserved genes methylated in both Nasonia and Apis.**

| GO-ID | Term | Category* | P-Value | FDR |
| --- | --- | --- | --- | --- |
| GO:0044424 | intracellular part | C | 3.6E-9 | 1.6E-5 |
| GO:0032991 | macromolecular complex | C | 6.6E-9 | 1.6E-5 |
| GO:0043170 | macromolecule metabolic process | P | 6.7E-8 | 1.1E-4 |
| GO:0044260 | cellular macromolecule metabolic process | P | 1.6E-7 | 2.0E-4 |
| GO:0043234 | protein complex | C | 6.6E-7 | 5.6E-4 |
| GO:0044237 | cellular metabolic process | P | 7.0E-7 | 5.6E-4 |
| GO:0005622 | Intracellular | C | 8.3E-7 | 5.7E-4 |
| GO:0044446 | intracellular organelle part | C | 9.0E-7 | 5.6E-4 |
| GO:0044428 | nuclear part | C | 1.1E-6 | 5.9E-4 |
| GO:0008152 | metabolic process | P | 1.2E-6 | 5.9E-4 |

*F=Molecular function C = cellular component P= Biological process
