# Supplementary material for: Function and Evolution of DNA Methylation in Nasonia vitripennis
Source: PLoS Genet. 2013 Oct 10;9(10):e1003872. doi: 10.1371/journal.pgen.1003872 (PMC3794928; doi:10.1371/journal.pgen.1003872)
Supplement: Table S17 — Enriched GO terms amongst Nasonia methylated genes that are conserved but not methylated in Apis. (DOC) [file pgen.1003872.s042.doc]

## Table S17: Enriched GO terms amongst *Nasonia* methylated genes that are conserved but not methylated in Apis.

| **GO-ID** | **Term** | **Category*** | **P-Value** | **FDR** |
| --- | --- | --- | --- | --- |
| GO:0030529 | ribonucleoprotein complex | C | 6.9E-6 | 3.4E-2 |

*F=Molecular function C = cellular component P= Biological process
