# Supplementary material for: Function and Evolution of DNA Methylation in Nasonia vitripennis
Source: PLoS Genet. 2013 Oct 10;9(10):e1003872. doi: 10.1371/journal.pgen.1003872 (PMC3794928; doi:10.1371/journal.pgen.1003872)
Supplement: Table S18 — Duplicated genes in Nasonia and their methylation status. (DOC) [file pgen.1003872.s043.doc]

## Table S18: Duplicated genes in *Nasonia* and their methylation status.

|  | Methylated in both *Nasonia* copies | Methylated in one copy (Loss/Gain) | Non-methylated in both Nasonia copies |
| --- | --- | --- | --- |
| Methylated in *Apis* | 9 | 8 | 3 |
| Non-methylated in *Apis* | 1 | 3 | 9 |
