# Supplementary material for: Function and Evolution of DNA Methylation in Nasonia vitripennis
Source: PLoS Genet. 2013 Oct 10;9(10):e1003872. doi: 10.1371/journal.pgen.1003872 (PMC3794928; doi:10.1371/journal.pgen.1003872)
Supplement: Text S1 — Validation of methylation status estimated from WGBS-seq with single-gene bisulfite sequencing using cloning method. (DOC) [file pgen.1003872.s044.doc]

## Text S1. Validation of methylation status estimated from WGBS-seq with single-gene bisulfite sequencing using cloning method.

To determine whether the methylation percentages estimated from the WGBS-seq data were accurate, we confirmed the methylation status for three randomly chosen methylated genes and three non-methylated genes, using bisulfite sequencing by cloning method (See Materials and Methods). Three out of three non-methylated genes (Nasvi2EG001314, Nasvi2EG000207 and Nasvi2EG006064) were confirmed to be non-methylated in the 5’-coding regions, consistent with the methylation status from the WGBS-seq data (Figures S4-S6). Three out of three methylated genes (Nasvi2EG002725, Nasvi2EG000295 and Nasvi2EG003593) were also verified in adult female samples, and the methylation percentages estimated from the cloning method matched well with the WGBS-seq data at covered CpG sites (Figures S7-S9). These results confirm that the WGBS-seq data analysis provided accurate methylation percentage quantification.
