# Supplementary material for: Function and Evolution of DNA Methylation in Nasonia vitripennis
Source: PLoS Genet. 2013 Oct 10;9(10):e1003872. doi: 10.1371/journal.pgen.1003872 (PMC3794928; doi:10.1371/journal.pgen.1003872)
Supplement: Text S2 — mCpGs are organized in methylated CpG clusters while non-coding and CpG islands are non-methylated. (DOC) [file pgen.1003872.s045.doc]

## Text S2. mCpGs are organized in methylated CpG clusters while non-coding and CpG islands are non-methylated.

Methylated CpGs are clustered in the *Nasonia* genome, and we defined the clustered methylated CpGs with >80% mCpGs and >40% average methylation percentage in the regions as mCpG clusters. To compare the mCpG clusters with the mammalian CpG islands, we ran predictions of CpG islands in the *Nasonia* genome using the same criteria as in the mammalian species (See Materials and Methods) and found 9,265 CpG islands: 36.8% of these occurred outside of gene bodies whereas 63.2% occurred in gene bodies. Although the *Nasonia* CpG islands have high CpG density (1.9 fold of the genome average), they were nearly universally non-methylated (0.15% mCpGs, which is less than the average for intergenic regions of 0.2%). There were 5,440 mCpG clusters in the *Nasonia* genome. They account for 2.2% of the genome or 0.78% of the covered CpGs (Table 1). Consistent with the gene body methylation, we observed that 98.8% mCG clusters are in gene regions. Among the 65 mCG clusters in “intergenic” regions in OGS2, only 23 were not expressed. We found detectable expression levels for the remaining 42 regions in adult female RNA-seq data, indicating that they may represent protein-coding regions not identified in OGS2 or non-coding RNAs. We therefore conclude that CpG islands in *Nasonia* occur in both transcribed and non-transcribed regions, but that methylation in CpG islands is associated with active transcription, and does not occur in untranscribed *cis*-regulatory regions.
