# Supplementary material for: Function and Evolution of DNA Methylation in Nasonia vitripennis
Source: PLoS Genet. 2013 Oct 10;9(10):e1003872. doi: 10.1371/journal.pgen.1003872 (PMC3794928; doi:10.1371/journal.pgen.1003872)
Supplement: Text S3 — DNA methylation and observed/expected CpG ratios. (DOC) [file pgen.1003872.s046.doc]

## Text S3. DNA methylation and observed/expected CpG ratios

In the genome, methylated Cs that have undergone spontaneous deamination will be repaired to Ts, resulting C-to-T point mutations and decreasing the observed/expected CpG ratio (CpG O/E ratio) in methylated regions. Insects that have CpG methylation in the genome display a bimodal distribution of the CpG O/E ratios, and the low and high CpG O/E classes correspond to the methylated and non-methylated genes respectively. Park *et. al.* (2011) found an association between CpG O/E and methylation status by cloning and sequencing 18 genes at selected CpG sites. We computed the CpG O/E ratios for all Nasonia genes and found a bimodal distribution, in which methylated genes perfectly matched the low CpG O/E class, and most non-methylated genes matched the high CpG O/E class (Figure S10A). In addition, the percentage of methylated sites was significantly correlated with CpG O/E ratios (Spearman's ρ = -0.61, *P*-value < 2x10^-16^) (Figure S10B). The GC content is also different for methylated and non-methylated genes. Methylated genes have slightly lower GC percent (mean = 41.1%, standard error = 0.08%) compared to the genome average (41.7%), whereas non-methylated genes have a much higher GC percent (47.8%, standard error = 0.09%) (Figure S10C). The CpG O/E ratio for methylated genes was positively correlated with GC percent (Spearman's ρ = 0.47, *P*-value < 2x10^-16^). Non-methylated genes with GC percent lower than the genome average were also positively correlated with CpG O/E (Spearman's ρ = 0.17, *P*-value < 2.2x10^-16^) just like the methylated genes, but those with higher GC percent were not correlated with CpG O/E (Spearman's ρ = -0.01, *P*-value = 0.36) (Figure S10C).
