# Supplementary material for: Function and Evolution of DNA Methylation in Nasonia vitripennis
Source: PLoS Genet. 2013 Oct 10;9(10):e1003872. doi: 10.1371/journal.pgen.1003872 (PMC3794928; doi:10.1371/journal.pgen.1003872)
Supplement: Text S4 — Quantification and validation of non-CpG methylation in the Nasonia genome. (DOC) [file pgen.1003872.s047.doc]

## Text S4. Quantification and validation of non-CpG methylation in the Nasonia genome.

***-*** ***Quantification of unconverted Cs in non-CpG context.*** Significant levels of non-CpG methylation (methylated cytosines in non-CpG context) have been observed in plants, human ES-cell lines and other insects. To check whether non-CpG methylation is present at a detectable level in the Nasonia genome, we quantified the methylation percentage at non-CpG positions by counting the number of unconverted Cs at non-CpG sites. At 71,488,414 non-CpG positions in the genome, we covered 59,355,138 Cs (83%) with a total of 630,815,952 high quality base calls in the WGBS-seq reads. Among them, 1,157,722 (0.18%) remained unconverted Cs. There were 21,839,978 covered non-CpG sites with read coverage of 10 or more, and the percentage of unconverted Cs was 0.19% (Table S5). Hence, the genome average for unconverted Cs in the non-CpG context is one-eighth of the CpG methylation percentage (1.45%).

***-*** ***Lack of non-CpG methylation at the whole genome level.*** In the literature, the percentage of unconverted Cs was used to estimate the non-CpG methylation percentage. However, not all uncovered non-CpG Cs were due to non-CpG methylation. There are four possible sources of unconverted non-CpG Cs:

(i). DNA methylation at non-CpG sites: these sites are the true positives for non-CpG methylation.

(ii). Unconverted Cs due to the bisulfite conversion efficiency. Not all non-methylated Cs were converted during the bisulfite conversion process. We estimated the bisulfite conversion efficiency to be 99.7% from the non-methylated lambda control DNA (Table S1, see Materials and Methods), thus the average unconverted Cs due to bisulfite conversion efficiency is expected to be 0.3%.

(iii). T-to-C Illumina sequencing errors in WGBS-seq. T-to-C sequencing error at non-CpG Cs is another possible source of observed unconverted non-CpG Cs. We estimated the T-to-C error rate (0.015%) from the lambda control DNA results (Table S1).

(iv). Unconverted Cs in non-CpG sites of the reference genome but in CpG context in the WGBS-seq samples. The individual samples used for WGBS-seq experiment may not have genome sequences identical to the reference genome, which will lead to spurious non-CpG Cs that are in fact in CpG context. First, segregating SNPs in the genome is one reason for the spurious non-CpG sites, but this is not a problem for Nasonia because inbreeding has made it highly homozygous. Another reason is due to sequencing errors in the reference genome, where a non-CpG site is actually a CpG site. Finally, SNPs in paralogous sequences that are not present in the reference genomes will also result in spurious non-CpG sites.

We used the WGBS-seq data to measure the effect of these sources of error, with the percentage from source (ii) and (iii) combined (0.315%) exceeds the percentage of unconverted Cs in the Nasonia genome (0.19%), suggesting no global non-CpG methylation in the Nasonia genome. However, we could not exclude the possibility of non-CpG methylation at a single-gene level, but these cases are likely to be very rare. We will still refer the unconverted non-CpG Cs as potential non-CpG methylation.

***-*** ***Distribution of potential non-CpG methylation percentage is different from CpG methylation.*** As for the CpG methylation, we defined methylated non-CpG sites as sites with >10% methylation and covered with 10 or more reads. Among the 21,839,978 covered non-CpG sites in the genome, only 49,728 (0.22%) were methylated. Compared to the CpG methylation percentage distribution which is skewed toward highly methylated sites (Figure S3), the methylation percentage was much lower at non-CpG sites, with no sites that are 100% methylated and 97% of the methylated sites had a methylation percentage less than 20% (Figure S10). The distribution of the percentage of unconverted Cs at non-CpG sites is consistent with the hypothesis that most of the observed non-CpG Cs were due to bisulfite conversion efficiency or T-to-C sequencing errors.

***-Candidate non-CpG methylation sites and validation.*** To check whether non-CpG methylation was present at particular sites in Nasonia genome, we generated a list of top candidate non-CpG methylation sites, with >30% unconverted Cs and >10X read coverage (Table S5). 28 (0.00013% of all covered non-CpG sites) such candidate sites were found in the genome, and eight of them (4 in top 10) were actually methylated at CpG sites due to unmatched reference genome sequences (Table S6 and Figure S11). 24/28 candidates were located in genic regions. We examined the CpG methylation in the 24 candidate methylated non-CpG harboring genes and 21 (87.5%) were methylated at CpG sites in the coding regions or introns (Table S6). Among the 28 candidate methylated non-CpG sites, 20 were in the middle and 3 were within 1 kb of methylated CpG clusters (mCGCLs). Therefore, the candidate non-CpG methylation sites tend to associate with methylated genes and they were often surrounded by CpG methylation (Table S6).

We selected four candidate non-CpG methylation sites for validation with single gene bisulfite sequencing by the cloning method and only one of them was methylated at the non-CpG site (Figure S12). A candidate non-CpG methylation site (on SCAFFOLD6 at position 1,767,201) was in the CAT context on the minus strand in the coding region of the gene Nasvi2EG004247, with 42% unconverted Cs estimated from the WGBS-seq data (Table S6 and Figure S12). Sixteen percent (3/19) of clones were confirmed to have unconverted Cs, suggesting the presence of non-CpG methylation at this site (Figure S12). The low validation rate and the large methylation percentage difference between WGBS-seq and cloning method indicated that the non-CpG methylations are rare and have more among individual variability compared to CpG methylation.
