# Supplementary material for: Function and Evolution of DNA Methylation in Nasonia vitripennis
Source: PLoS Genet. 2013 Oct 10;9(10):e1003872. doi: 10.1371/journal.pgen.1003872 (PMC3794928; doi:10.1371/journal.pgen.1003872)
Supplement: Text S5 — Logistic regression analysis of the effect of expression and expression breadth on gene methylation status. (DOC) [file pgen.1003872.s048.doc]

## Text S5. Logistic regression analysis of the effect of expression and expression breadth on gene methylation status.

Expression level and expression breadth are also correlated, and highly expressed genes tend to have broader expression pattern across stages. To disentangle the effect sizes of expression level and expression breadth on methylation status, a formal statistical test is needed. We performed logistic regression analysis on the two variables separately as well as the full model (Table S7 and S8). In the model using both predictors, both coefficients are significantly different from 0 (*P* < 0.001, Likelihood ratio test). The expression level is positively correlated with methylation (coefficient = 2.68), whereas the expression CV is negatively correlated with methylation (coefficient = -1.70). Four ordinal measures of association for the two single variable models and the full model were computed (Table S7). The fitted full model has a strong prediction for methylation status measured by a concordance of 90.2 percent (and a Max-rescaled R^2^ of 0.518, Table S6). From the univariate logistic regression analysis, the expression level has a 89.3 percent of concordance (and a Max-rescaled R^2^ of 0.497, Table S8). Therefore, the expression level explains much more variability than the expression CV. This is consistent with what we saw intuitively from the plots (Figure 3A and 3C).
