# Supplementary material for: Function and Evolution of DNA Methylation in Nasonia vitripennis
Source: PLoS Genet. 2013 Oct 10;9(10):e1003872. doi: 10.1371/journal.pgen.1003872 (PMC3794928; doi:10.1371/journal.pgen.1003872)
Supplement: Text S6 — Potential links between DNA methylation and alternative splicing. (DOC) [file pgen.1003872.s049.doc]

## Text S6. Potential links between DNA methylation and alternative splicing.

We examined methylated genes with multiple methylated CpG clusters, to determine whether these are associated with alternative splicing. There are 344 genes that have more than one methylated cluster per gene. We examined the distribution of the mCpG clusters in these genes to determine whether mCpG clusters were associated with differential splicing, and found three classes of genes with potential associations between methylation and alternative splicing: (a) differential 5’ start usage, (b) differential middle exon usage, and (c) splice forms with intron retention.

(i). Alternative 5’ start sites are marked by DNA methylation: A subset of 215 genes have two or more mCpG clusters that are at least 350 bp apart, allowing us to test for associations of these clusters with alternative 5’ exon usage (Figure S24A and Table S14). In RNA-seq data, we detected expression from major spliced forms in the OGS2 gene models, with differential 5’ exon usage among them. For example the gene Nasvi2EG000107 has two alternative start sites, and two mCpG clusters that correspond to them (Figure S24A). Of 215 genes with multiple mCpG clusters, only 28 clearly demonstrated the association between methylation and the alternative 5’ exon usage (Table S14).

(ii). Differential middle exon usages: In a set of examined methylated genes with differential internal exon usage (Figure S24B), we observed that the alternatively spliced exon within the 5’ 1 kbp coding region was methylated (covered with mCpG clusters), whereas exons beyond the 5’ 1 kbp coding region were not methylated. An example is shown in Figure S24B.

(iii). Intron retention: Intron retention is a common form of alternative splicing where particular intron(s) are sometimes retained in the mature mRNA transcripts. In adult RNA-seq data, we found 2,841 introns with an average coverage of 5 or more, of which 1,383 were in methylated genes. Among these methylated genes, 228 have four or more methylated CpG sites in the expressed introns (Figure S24C), suggesting potential association between intron methylation and intron retention. However, methylated genes with high intron methylation (≥10% intron methylation percentage) do not show significantly higher intron RNA-seq read coverage (median coverage = 19.0) than the ones with >10 intron methylation (median coverage = 18.3, *P*-value = 0.898, Mann-Whitney U Test). Therefore, methylation is clearly not required for intron retention.
