# Supplementary material for: Function and Evolution of DNA Methylation in Nasonia vitripennis
Source: PLoS Genet. 2013 Oct 10;9(10):e1003872. doi: 10.1371/journal.pgen.1003872 (PMC3794928; doi:10.1371/journal.pgen.1003872)
Supplement: Text S7 — Calculation of global methylation (mC/C) in Nasonia, Apis and ants. (DOC) [file pgen.1003872.s050.doc]

## Text S7. Calculation of global methylation (mC/C) in Nasonia, Apis and ants.

In this study, we found 1.59% covered CpG (>=10X depth) to be methylated in *Nasonia* genome. To compare *Nasonia* methylation level with honeybee and ants, we calculated the percentage of mCpGs using data from two published papers.

In the honeybee methylome paper (Lyko *et al.* 2010), the percentage of methylated CpGs (mC/C) is not mentioned directly in the main text, but we could calculate it based on the following results in the paper: “More than 90% of the 10,030,209 CpGs in the Apis genome were covered by at least two sequencing reads, allowing for the methylation status of individual sites to be determined with confidence.” “First, of the over 60 million cytosines that exist in the Apis genome, only approximately 70,000 are methylated.” Therefore, the percentage of methylated CpG (mC/C) is 70,000/(10,030,209*90%) = 0.78% in honeybee.

In the ants methylome paper (Bonasio *et al*. 2012), it is stated “We detected cytosine methylation at ~200,000 sites in *Camponotus* and at ~250,000 sites in *Harpegnathos* (Figure 1A), accounting for 0.3% and 0.21% of all cytosines.” “More than 92.5% of all cytosines (Cs) were covered by at least two reads per sample.” From the genome sequences, there are 10,489,493 CpGs in *Camponotus* and 21,685,204 in *Harpegnathos*. So the percentage of methylated CpGs is 200,000/(10,489,493*92.5%) = 2.1% for *Camponotus* and 250,000/(21,685,204 *92.5%) = 1.25% for *Harpegnathos*. The authors in this paper also mentioned “After correcting for partially methylated sites, we determined the abundance of mCs at 0.14%–0.16% in *Camponotus* and 0.11%–0.12% in *Harpegnathos*.” If we corrected for that, the percentage of methylated CpGs is 1.05% in *Camponotus* and 0.68% in *Harpegnathos*.
